# Age-period-cohort analysis of ischemic stroke deaths attributable to physical inactivity in different income regions

**DOI:** 10.1038/s41598-024-57309-2

**Published:** 2024-03-19

**Authors:** Junjiao Liu, Yueyang Liu, Wenjun Ma, Jie Liu, Yan Tong, Cui Wang, Jianzhong Zheng

**Affiliations:** 1 College of Public Health, Shanxi Medical University, Taiyuan, Shanxi China; 2 Second Clinical Medical College, Nanchang University, Nanchang, Jiangxi China

**Keywords:** Diseases, Health care, Risk factors

## Abstract

This study assessed the global and regional burden of IS (ischemic stroke) deaths due to LPA (low physical activity) from 1990 to 2019, analyzed regional, sex, and age differences in ASMR (age-standardized mortality rate), and provided a comprehensive understanding of the impact of age, period, and cohort on low physical activity related ischemic stroke ASMR. We conducted an APC (age-period-cohort) analysis of the global and four World Bank income level regions’ IS mortality data attributed to LPA from 1990 to 2019, using the GBD2019 database, and the results showed that the global net drift of the Ischemic stroke age-standardized mortality attributable to low physical activity was − 1.085%[95% CI: − 1.168, − 1.003].The ASMR drop is most pronounced in the high-income zone, with a net drift of − 2.473% [95% CI: − 2.759, − 2.187] across the four income groups. The influence of age on mortality is increasing in the worldwide old population, while the period and cohort effects are decreasing. We also performed a Joinpoint regression analysis, which revealed that the specific time of considerable drop in ASMR of IS in the global LPA population was 2002–2007, with an APC of -2.628%. The specific period of considerable drop in ASMR in high-income regions with the highest variation was 1999–2007, with an APC = − 4.726%. The global burden of public health deaths caused by LPA is diminishing, with the most notable progress observed in high-income regions. However, in low and lower-middle income areas, the situation continues to deteriorate. Within the global elderly population, the effects of age on mortality is increasing, while the effects of period and cohort are diminishing. These trends vary across income levels, highlighting the necessity for enhanced international collaboration to formulate context-specific public health strategies aimed at enhancing cardiovascular health on a global, regional, and national scale.

## Introduction

The World Health Organization (WHO) has initiated a global Action plan on non-communicable diseases (NCDS) with the objective of diminishing premature mortality resulting from NCDS by 25% by the year 2025^1^. The third Sustainable Development Goal of the United Nations endeavors to reduce untimely deaths caused by non-communicable diseases by one-third by the year 2030^2^. Cardiovascular disease (CVD) stands as the primary cause of morbidity, disability, and mortality on a global scale, thereby leading to significant health disparities^3^. The impact of Stroke as a disease burden should not be underestimated.

Stroke is a prevalent medical condition characterized by a substantial occurrence rate, significant disability rate, elevated mortality rate, and a notable recurrence rate, commonly referred to as the “four high” characteristics. Globally, it is estimated that one in every four individuals will experience a stroke, resulting in a distressing fatality every six seconds and a disabling event every six seconds. Consequently, stroke imposes considerable economic burdens and physical and psychological suffering upon the affected individuals and their families.

Stroke encompasses two main types, namely ischemic stroke and hemorrhagic stroke, with ischemic stroke constituting approximately 85% of all reported cases in epidemiological studies^4^. Consequently, the United Nations has designated IS (ischemic stroke) as a priority target in an effort to lower the burden of non-communicable diseases. The etiology of IS (ischemic stroke) is multifactorial; risk factors for IS include high blood pressure, hyperlipidemia, diabetes, smoking, alcohol use, obesity, and lack of exercise. Of these, lack of exercise is one of the most cost-effective controllable risk factors. Previous research has demonstrated that a lower level of physical activity is linked to a higher risk of stroke^5,6^. A study conducted by the National Health and Nutrition Examination Survey (NHANES) also revealed a link between a lower incidence of stroke and various forms, frequency, and intensities of physical exercise^7^.

In addition, For the following primary reasons, physical inactivity has been identified as the largest worldwide public health issue of the twenty-first century: (1) Among risk factors for increased mortality from NCDS worldwide, physical inactivity ranks fourth^8,9^. It is closely linked to the development of non-communicable diseases as diabetes, cancer, and cardiovascular disease. The body cannot receive enough exercise from insufficient physical activity, which raises the risk of disease^10^. (2) The World Health Organization's Global Status Report on Physical Activity 2022 estimates that 1.4 billion adults globally—or roughly 27.5% of the adult population—do not engage in enough physical activity, a statistic that has essentially stayed constant over time^11,12^. It indicates that a significant portion of the global population leads unhealthy lifestyles. If current trends continue, approximately 500 million people worldwide will experience chronic illnesses like diabetes, obesity, and heart disease by 2030 as a result of insufficient physical activity. This will surely place a strain on public health systems because unhealthy lifestyles result in an excessive use of medical resources^10^. (3) People of all ages, including toddlers, adolescents, adults, and the elderly, struggle with inadequate physical activity. This indicates that it is a global issue that requires the consideration and resolution of the entire community. (4) Physical inactivity has a substantial financial cost; in low and middle income countries, over three-quarters (74%) of chronic illnesses linked to physical inactivity occur^13^.

Globally, LPA (low physical activity) was linked to 7.2% of deaths from all causes and 7.6% of deaths from cardiovascular disease. The proportion of NCDS due to LPA ranged from 1.6% of hypertension to 8.1% of dementia^14^.

Physical activity (PA) and the risk of stroke morbidity or mortality have been found to be inversely correlated in prior case–control and cohort studies^15,16^. Physical activity can lower the risk of stroke by 25–30%, according to the Physical Activity Guidelines Advisory Committee^17^. Furthermore, a nationwide cohort research conducted in South Korea discovered that, after high blood pressure, a lack of moderate to intense physical exercise was the second biggest risk factor for stroke^18^. For the majority of individuals globally, physical inactivity is a major cause of morbidity, early death, and mortality as well as a common risk factor^19,28^. It has become epidemic and is strongly associated with major non-communicable diseases^20,21^, particularly cardiovascular diseases like stroke^22^. While the global age-standardized death rates from IS as a result of LPA (low physical activity) have decreased over the last three decades, they have increased overall for all age groups, which is indicative of the growing burden of population expansion and aging^23,24^. Furthermore, there are regional and national differences in the incidence and mortality of ischemic stroke, particularly in areas with varying income levels. Finding manageable risk factors for mortality and the areas where it is dropping most is crucial for reducing the health gap between high- and low-income nations. Other important steps to take include focusing on more effective preventative methods and health-care management^25^.

Given the above, in order to meet the Sustainable Development Goals by 2030, existing efforts will not be sufficient. Furthermore, regular annual reassessments of ischemic stroke are essential to assessing success in prevention because the condition is effectively avoidable, populations are aging globally, and epidemiological demographic factors are changing annually^4^.

While we can concentrate on a single aspect of trend research, like variations in mortality across time, practical investigations invariably take age, period, and cohort into account. The fundamental premise when age and cohort are disregarded and period changes are examined is that the composition of age and cohort is substantially the same for every observed period, or that age and cohort differences do not significantly affect the observed dependent variable^26^. Since this assumption is frequently untrue in practice, variations in cohort makeup may also introduce cohort effects into the observed changes in mortality across time. Consequently, the age-period-cohort (APC) analysis—which separates potential cohort and age effects—is required to fully comprehend variations in mortality in the period dimension.

Of course, identifying trends is not the only goal of APC analysis. To comprehend the mechanism of trend creation and its influencing components, it is imperative to ascertain the actual age, period, and cohort effects. Age affects are alterations in social, psychological, and physical status brought on by variations in biological age. Period effect is a term used to describe the changes that occur as a period changes. These changes are typically brought about by the immediate impact of external environmental factors, such as significant historical events, shifts in the social and economic environment, and advancements in new technologies^27^. The period effect's consistent dissemination throughout all age groups at the same time is its most remarkable characteristic^27^. That being said, the cohort effect focuses its impact on cohorts of people who were born around the same time. Through the interaction of internal (such as the individual development process) and external (such as major social events) forces, some historical events or social changes that these individuals experienced over time left a gradual imprint on them that affects their level of happiness^28^. The mechanism of action of macro factors is different in time and cohort. If the period trend reflects the transient effects of external factors, then the cohort trend reflects the interaction of external factors with the internal development of individuals, that is, external historical factors act on individuals at different life stages, thus producing differentiation in the population. Therefore, we need to explain the utility of these macro factors in the cohort with a life-course perspective^29^.

In order to comprehend and grasp the temporal trend and significant change points of IS mortality in regions with varying income levels, as well as the influence of age, period, and cohort on IS mortality, this study used the APC and Joinpoint methods to analyze the disease burden of IS mortality attributable to LPA globally and in four different World Bank Income levels regions. It is helpful to clarify the trend and characteristics of IS mortality over time in order to support local governments and health departments in creating effective risk factor prevention strategies and focusing on healthy aging throughout the life cycle in accordance with the particular circumstances of the region in order to raise public health standards generally, lower the burden of IS disease, improve IS mortality, and enhance the quality of life for IS patients^28^.

## Methods

### Data source and definitions

Our data are all sourced from the GBD 2019 free database, provided by the Institute for Health Metrics and Evaluation (IHME), which reports incidence, prevalence, causes of death, mortality and risk factors for 204 countries and territories from 1990 to 2019. The objective is to quantify the relative extent of health loss due to disease, injury and risk factors in specific populations, disaggregated by age, sex and geographical location, over a given period of time^25,30,31^. All the data are publicly accessible in IHME websites, (http://ghdx.healthdata.org/gbd-results-tool)^25,30^. GBD 2019 uses DisMod-MR 2.1(a Bayesian meta-regression tool) as the primary estimation method to ensure consistency among incidence, prevalence, mortality and causes of death across conditions. It promotes comparability and systematically of information, facilitates estimation of outcomes in countries with incomplete data, and used to report burden of disease standardized indicators^30,31^.

In the GBD 2019 database, we selected IS (ischemic stroke) as the cause, LPA (low physical activity) as the risk factor, Deaths as the Measure, and Male, Female and Both as the gender. As for Age We have selected 14 age groups: 25–29, 30–34, 35–39, 40–44, 45–49, 50–54, 55–59, 60–64, 65–69, 70–74, 75–79, 80–84, 85–89 and 90–94. Mortality was calculated from age 25, as there were no IS-related deaths attributable to LPA in younger age groups^28^. Location selected Global and 4 World Bank income levels regions, including the World Bank High Income region, the World Bank Upper Middle Income region, the World Bank Lower Middle Income region and the World Bank Low Income region. Finally, the time frame was chosen from 1990 to 2019.

According to GBD 2019, there are four levels of physical activity based on total MET minutes: inactivity (< 600 MET minutes per week = , low activity (600–3999 MET minutes per week), moderate activity (4000–7999 MET minutes per week), and high activity (≥ 8000 MET minutes per week)^32^. Low physical activity is defined by GBD 2019 as an average weekly physical activity (at work, home, while transportation, and for leisure) of less than 3000 MET-min^28^.

In calculating the ASR (age-standardization rate), we used the 2019 global population composition as the standard population^33^. Taking into account uncertainties due to primary sources, data errors, and data manipulation, the GBD study calculated the uncertainties of all estimates and quantified them with a 95% uncertainty interval (UI)^34^. The UI is calculated based on 1000 drawing levels of the model's posterior distribution, with 95%UI defined as the 2.5 and 97.5 values of the distribution. Both mortality and the corresponding ASR were reported with a 95% uncertainty interval (UI)^35^.

### Assessment methods of IS disease burden attributed to various risk factors

For each risk factor, the GBD2019 study quantifies the proportion of disease burden that could have been prevented if exposure levels were maintained at the minimum risk level, defined as the theoretical minimum risk exposure level (TMREL)^36,37^. The population attributable fraction (PAF) is obtained by comparing the theoretical minimum risk exposure level with the exposure level of specific population on the premise that the exposure level of other risk factors remains unchanged^38^. According to the framework of comparative risk assessment theory, GBD 2019 calculates the attributable disease burden caused by the main risk factors of IS with indicators such as PAF and DALYs of IS disease burden.

The number of IS deaths attributed to LPA can be calculated by multiplying the PAF of LPA by the number of IS deaths. Age-standardized mortality rates for different regions, years and genders were calculated using the world standard population structure for 2000–2025^39^.

### Statistical analysis

#### APC

An age-period-cohort (APC) model was used to analyze trends in IS deaths attributable to LPA globally and in four World Bank Income Levels regions, and the effects of age, period, and cohort on mortality. This approach has been widely used in descriptive epidemiological analysis of various chronic diseases, helping to describe and explain long-term trends in chronic disease mortality over time, as well as to identify disease trends that are related to age-related biological factors, social influences, and technological contributions^28,40–42^. APC analysis can estimate the trend effect of age, period and cohort at the same time, and eliminate the mutual interference factors among them, effectively avoiding the problem of complete collinearity among the three dimensions of age, period and cohort.

In the APC model, the input data were the IS mortality rates attributed to LPA globally and in 4 World Bank Income Levels regions from 1990 to 2019. Age and period intervals in the APC model have to be equal, so we’ve determined six periods with 5-year intervals: 1990–1994, 1995–1999, 2000–2004, 2005–2009, 2010–2014, and 2015–2019. The 14 age groups over 25 that were chosen at 5-year intervals from the GBD 2019 database are referred to as age groups. For the birth cohort, due to different model algorithms, there are two situations. In the age, period and cohort interaction study, the birth cohort is divided into groups with 5-year intervals, consisting of 19 cohort groups from 1900–1904 to 1990–1994; in the age, period and cohort effect study, the birth cohort is divided into groups at 10-year intervals, from 1895–1904 to 1985–1994^28,40^. But this difference in algorithm does not affect the results of the study.

We used the APC model to calculate net drift, local drift, longitudinal age curves, and ratios rate (RR) of IS mortality across periods (period effects) and cohort (cohort effects). The net drift represents the annual percentage change over time in the age-standardized mortality rate of IS, indicating an overall log-linear trend by period and cohort^43^. Local drift represents the annual percentage change over time in age-standardized mortality rates of IS for each age group. The longitudinal age curve represents the expected age-specific rate in the reference cohort adjusted for period effects^43^. Period effects refer to the relative risk of IS mortality in different periods in the study area. PRR (period rate ratios, *p*/*p*_*0*_) is the ratio of age-specific rates in period *p* relative to reference period *p*_*0*_. Cohort effects refer to the relative risk of IS mortality in different birth cohorts in the study area. CRR (cohort rate ratios, *c*/*c*_*0*_) is the ratio of age-specific rates in cohort *c* relative to reference cohort *c*_*0*_. In addition, the reference period of this study is 2000, and the reference age is 50 years old.

We performed our analysis through the National Cancer Institute's Age Cycle Network tool, which uses weighted least squares statistical methods and assumes that counting data follow a Poisson distribution^28^.

#### Joinpoint

A joinpoint regression model was used to o investigate temporal trends in the burden of IS deaths attributable to LPA between 1990 and 2019^28^. Through this method, we can identify the significant change points ((join points) in the time change trend, and obtain the APC and AAPC values^28^. AAPC acts as an aggregate measure of trends in the burden of IS deaths over a fixed time interval and is calculated as a weighted average of annual percentage change (APC)^28^. AAPC values and their corresponding 95% confidence intervals (CI) reflect trends in age-standardized mortality^44^. In addition, the Wald χ^2^ test was given in the joinpoint regression model to assess statistical significance. All statistical tests were double-tailed and statistical significance was defined as P < 0.05. Data analysis was performed using R software version 4.2.3, while the join point regression program version 4.9.1.0 was used to perform the join point regression model and trend analysis^28,44^.

### Ethical statement

The data used in this study are all publicly available data in GBD2019, and the GBD study was reviewed and approved by the University of Washington's Institutional Review Board.

### Ethical committee

The study was compliant with the Guidelines for Accurate and Transparent Health Estimates Reporting, and the University of Washington Institutional Review Board reviewed and approved the waiver of informed consent for GBD 2019.

## Results

### Global and regional trends in the mortality of Ischemic stroke attributable to Low Physical Activity, 1990–2019

**Figure 1:**
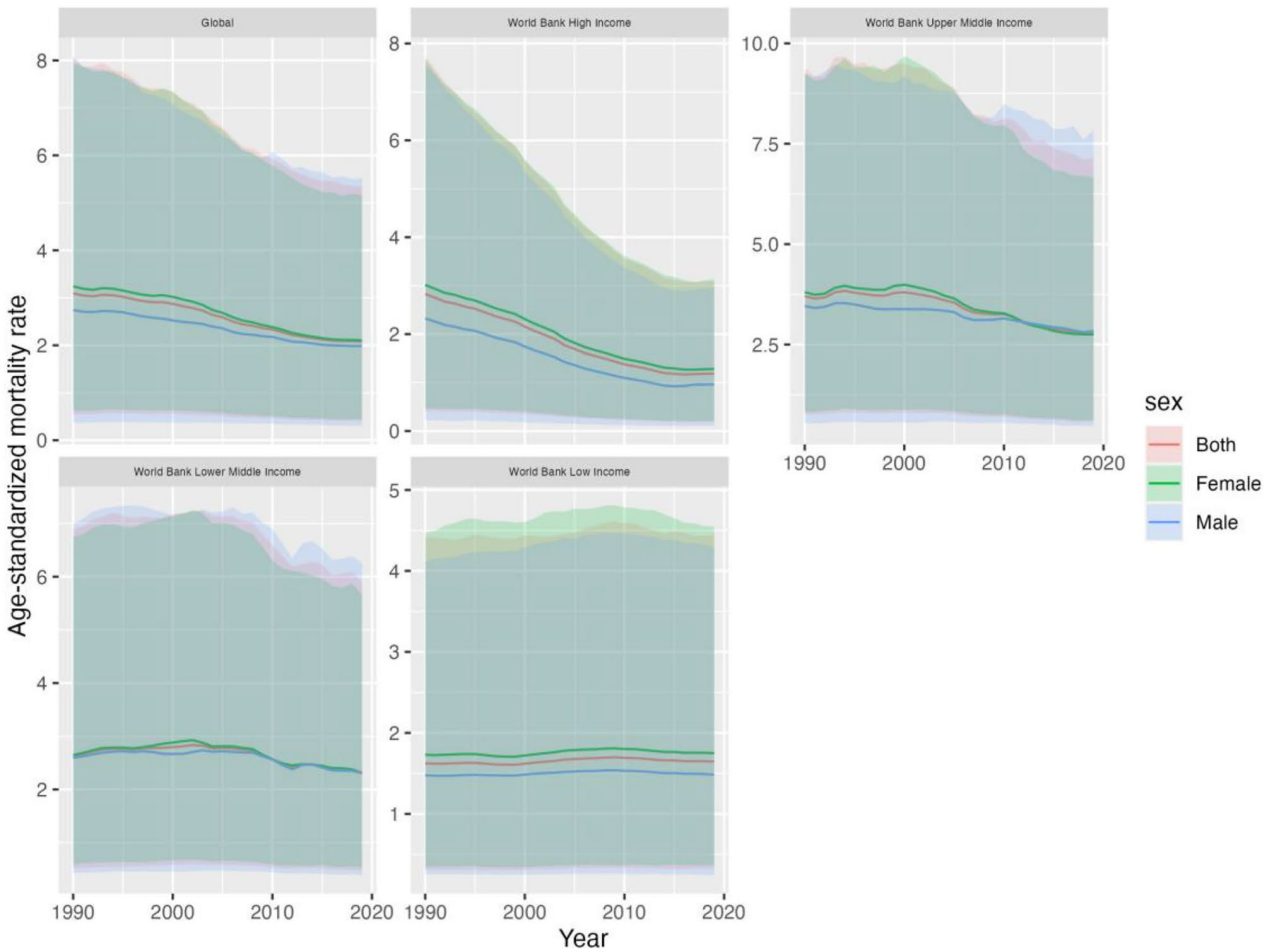
Trends in the mortality from Ischemic Stroke attributable to low physical activity in global and four world bank income levels regions, from 1990 to 2019.

Figure 1, Table 1, Supplementary Tables 1 and 4 presents the mortality from IS (ischemic stroke) attributable to LPA (low physical activity) at the global and four different World Bank income levels. It includes the death rate, number of deaths, number change rate, ASMR (age-standardized mortality rate), ASMR change rate, as well as net drift in mortality estimated by APC (web) tools.

**Table 1:** Trends in mortality of Ischemic stroke attributable to low physical activity across Global and 4 World Bank Income Levels regions, 1990–2019.

Characteristics	1990		2019		1990–2019		
	Deaths number	ASMR	Deaths number	ASMR	Num change	ASR change	Net drift
	95%UI						
Global	87,646 (15,903 to 231,493)	3.095% (0.55 to 8.091)	152,395 (30,078 to 391,946)	2.08% (0.405 to 5.335)	0.739% (0.581 to 1.022)	− 1.606% (− 1.71 to 1.502)	− 1.085% (− 1.168 to 1.003)
Male	31,044 (4354 to 90,733)	2.743% (0.375 to 8.056)	59,841 (9271 to 168,138)	1.986% (0.311 to 5.52)	0.928% (0.682 to 1.357)	− 1.303% (− 1.372 to 1.234)	− 0.854% (− 0.969 to 0.739)
Female	56,602 (11,154 to 140,230)	3.243% (0.638 to 7.94)	92,553 (19,375 to 226,863)	2.102% (0.44 to 5.154)	0.635% (0.467 to 0.932)	− 1.749% (− 1.873 to 1.625)	− 1.320% (− 1.440 to 1.200)
World Bank High Income	35,218 (5211 to 96,888)	2.83% (0.418 to 7.741)	34,831 (5364 to 90,976)	1.182% (0.187 to 3.084)	− 0.011% (− 0.116 to 0.167)	− 3.470% (− 3.646 to 3.294)	− 2.473% (− 2.759 to 2.187)
World Bank Upper Middle Income	35,577 (7046 to 92,288)	3.707% (0.767 to 9.371)	77,055 (15,394 to 199,579)	2.805% (0.576 to 7.145)	0.944% (0.702 to 1.301)	− 1.164% (− 1.338 to 0.99)	− 1.666% (− 1.825 to 1.507)
World Bank Lower Middle Income	15,432 (2972 to 41,191)	2.628% (0.514 to 6.883)	37,092 (7689 to 97,067)	2.31% (0.479 to 5.901)	1.4% (0.992 to 1.89)	− 0.56% (− 0.729 to 0.384)	0.116% (− 0.026 to 0.259)
World Bank Low Income	1368 (255 to 3910)	1.622% (0.309 to 4.414)	3315 (629 to 9394)	1.644% (0.317 to 4.434)	1.277% (1 to 1.589)	0.128% (0.071 to 0.185)	0.262% (− 0.135 to 0.66)

The global number of IS deaths attributable to LPA increased from 87,646 to 152,395 [95%UI: 30,788, 391,946], an increase of 0.739% [95%UI: 0.581, 1.022] between 1990 and 2019, as shown in Fig. 1 and Table 1. In contrast, ASMR decreased from 3.095% [95% UI: 0.55, 8.091] to 2.08% [95% UI: 0.405, 5.335], decreasing by − 1.606% [95% UI: − 1.71, − 1.502].The number of male deaths increased from 31,044 [95%UI: 4354, 90,733] to 59,841 [95%UI: 9271, 168,138] globally, a growth rate of 0.928% [95% UI: 0.682, 1.357], the male ASMR decreased from 2.743% [95% UI: 0.375, 8.056] to 1.986% [95% UI: 0.311, 5.52], and a decrement of − 1.303% [95% UI: 1.372, 1.234]; The number of female deaths increased from 56,602 [95%UI: 11,154, 140,230] to 92,553 [95%UI:19,375, 226,863] with a growth rate of 0.635% [95%UI: 0.467, 0.932], female ASMR decreased from 3.243% [95% UI: 0.638, 7.94] to 2.102% [95% UI: 0.44, 5.154], a decrease rate of − 1.749% [95% UI: − 1.873, − 1.625]. Globally, female experience higher ASMR and a higher death rate than male. The number of IS deaths linked to LPA decreased in high-income regions among the four World Bank income levels regions, from 35,218 [95%UI: 5211, 96,888] in 1990 to 34,831 [95%UI: 5364, 90,976] in 2019, a decrement of − 0.011% [95% UI: − 0.116, 0.167], and ASMR reduced from 2.83% [95% UI: 0.418, 7.741] to 1.182% [95% UI: 0.187, 3.084], a decrease rate of − 3.470% [95% UI: − 3.646, − 3.294]; The number of IS deaths attributable to LPA increased in the upper middle income region from 35,577 [95%UI: 7046, 92,288] in 1990 to 77,055 [95%UI: 15,394, 199,579], growing at a rate of 0.944% [95%UI: 0.702, 1.301], while the number of ASMR deaths f fell by − 1.164% [95% UI: − 1.338, − 0.99] from 3.707% [95%UI: 0.767, 9.371] to 2.805% [95% UI: 0.576, 7.145]; The number of IS deaths attributable to LPA in lower middle income region increased from 15,432 [95%UI: 2972, 41,191] in 1990 to 37,092 [95%UI: 7689, 97,067], a growth rate of 1.4% [95%UI: 0.992–1.89],and ASMR decreased from 2.628% [95%UI: 0.514, 6.883] to 2.31% [95%UI: 0.479, 5.901], the decline rate was − 0.56% [95%UI: − 0.729, − 0.384]; The number of IS deaths attributable to LPA in low income region increased from 1368 [95%UI: 255, 3910] in 1990 to 3315 [95%UI: 629, 9394], a growth rate of 1.277% [95%UI: 1, 1.589], and ASMR increased from 1.622% [95%UI: 0.309, 4.414] to 1.644% [95%UI: 0.317, 4.434], with a growth rate of 0.128% [95%UI: 0.071, 0.185] (Fig. 1, Table1).

Overall, with a rate of change of − 0.011% [95% UI: − 0.116, 0.167], high income region was the only region in the global and among the four world Bank income levels regions to see a negative rise in deaths between 1990 and 2019. Meanwhile, the greatest rate of rise in deaths (1.4% [95%UI: 0.992, 1.89]) was observed in lower middle income areas. Similar to this, the global and the other three areas with the World Bank Income levels exhibited a declining trend in terms of ASMR, with the exception of low income regions (0.128% [95% UI: 0.071, 0.185]), and high income regions saw the biggest reduction in ASMR (− 3.470% [95% UI: − 3.646, − 3.294]).

### APC study of the mortality from Ischemic strokes linked to low physical activity

#### Trends in IS mortality attributable to LPA by age and cohort

**Figure 2:**
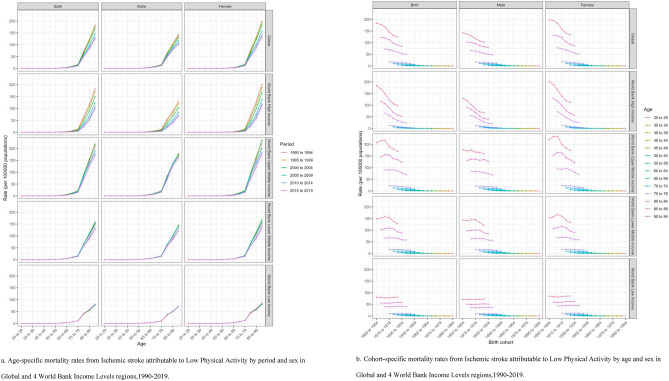
Age-specific (**a**) and Cohort-specific (**b**) mortality rates of Ischemic stroke attributable to Low Physical Activity in Global and 4 World Bank Income Levels regions,1990–2019.

The pattern of the IS death rate rising with age is depicted in Fig. 2a. We may observe that while the death rate from IS has grown somewhat internationally overall for those over 80, it has fallen dramatically when compared to the 1990–1994 period. The pattern of increase is similar in high-income areas. The age group over 85 years old in the upper-middle income area showed a notable growth between 2000 and 2004. The age group over 85 saw a notable increase in the low-income area between 2000 and 2004, albeit at a slower rate than in the upper-middle income area. The IS mortality rate for people over 80 in low-income areas started to exhibit a notable rising trend in all 6 periods, while each period's difference was not very noticeable.

With the exception of low-income regions, Fig. 2b demonstrates that the death rate from IS in the birth cohort of patients of all ages decreased globally and in the other three World Bank income level regions, suggesting a relatively low risk of IS death in these regions’ recently born cohort of all ages. All of these regions' birth cohorts, both male and female, showed differing degrees of reduction. Nonetheless, the birth cohort of patients aged 75–94 years showed an increase in the death rate of IS in low-income areas, suggesting a higher death rate in the most recent birth cohort in these age groups. In low-income areas, birth cohorts for both genders, aged 75–94, indicated a little increase trend. It implies that IS patients between the ages of 75 and 94 have a higher chance of dying in low-income locations, regardless of gender.

#### The age, period, and cohort effects on Ischemic stroke mortality attributable to Low Physical Activity were analyzed by APC (web)

**Figure 3:**
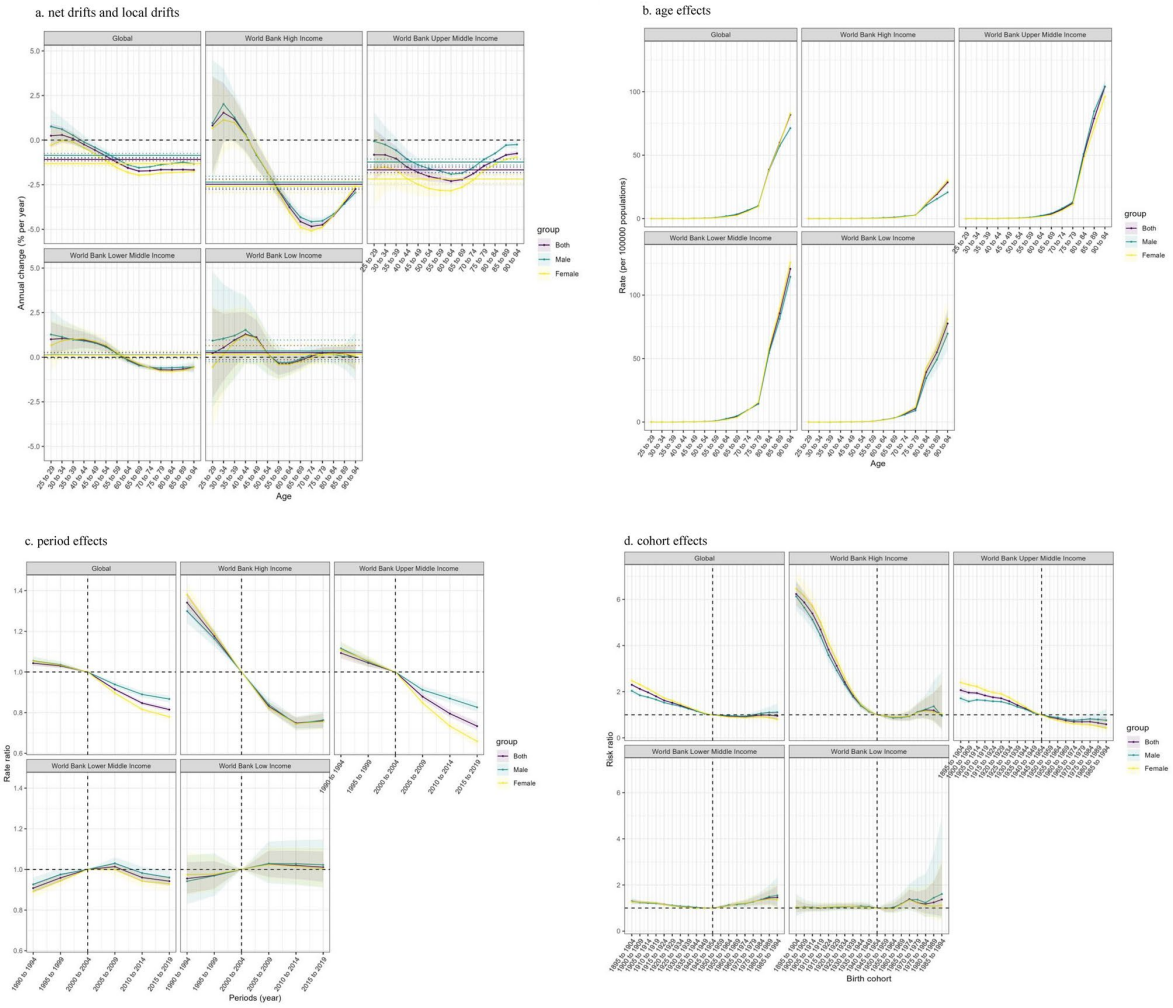
The net drifts and local drifts (**a**), age effects (**b**), period effects (**c**), and cohort effects (**d**) of Ischemic stroke attributable to Low Physical Activity in the Global and 4 World Bank Income Levels regions, 1990–2019.

Figure 3, Supplementary Tables 4 and 5 shows the results of the APC analysis of IS (Ischemic stroke) deaths attributable to LPA (Low Physical Activity) globally and in 4 different World Bank Income levels regions. The main relevant indicators are net drifts, local drifts, age effects, period effects and cohort effects. Net drifts are the analogue of the estimated annual percentage change, or EAPC, of the age-standardized rate (ASR). Compared to EAPC, net drifts provide a more accurate representation of the average annual rate of change since they account for age, period and cohort effects. Local drift estimated the annual percentage change of IS deaths over time specific to age groups in the study area. Age effects are expressed as a longitudinal age curve. It adjusted for period deviations and thus more accurately describes natural variations in IS mortality in the study area.

According to the APC analysis, the global net drift in IS mortality attributable to LPA was − 1.085% [95% CI: − 1.168, − 1.003]. Among them, it was − 0.854% [95% CI: − 0.969, − 0.739] for males and − 1.320% [95% CI: − 1.440, − 1.200] for females, indicating a relatively significant reduction in IS mortality in females globally. For the four different World Bank Income levels regions, the mortality reduction of IS was most significant in high-income regions, with a net drift of − 2.473% [95% CI: − 2.759, − 2.187]. While the increase in IS mortality was relatively significant in low-income areas (0.262% [95% CI: − 0.135, 0.660]), followed by lower-middle-income areas (0.116% [95% CI: − 0.026, 0.259]) (Fig 3a).

Regarding local drift, the global local drift values for every age group of females were less than zero, suggesting a global trend of declining IS mortality among females of all ages related to LPA. Furthermore, we can observe that the drop in IS death rates for both males and females over the age of 70 has slowed globally. Furthermore, we discovered that only the upper-high income regions—of the four World Bank income levels regions—had local drift values for both male and female that were less than 0. This suggests that the IS death rate in these regions declined to varying degrees for all age groups, with the rate of decline slowing down more rapidly from 35 to 64 years old and progressively slowing down beyond 65. Local drift values for the IS death rate between 25 and 54 years old were higher than 0 in low-income areas, suggesting an increasing trend to varying degrees for this age group; local drift values for the IS death rate between 55 and 69 years old were lower than 0, suggesting a decreasing trend to varying degrees for this age group; local drift values for the IS death rate over 70 years old are higher than 0. It demonstrated that, to varying degrees, the death rate of IS over 70 exhibited a growing tendency. In the other two regions, the IS death rate of the low age group increased in different degrees, and the IS death rate of the high age group decreased in varying degrees. The difference lies in the age point where the mortality trend turns from rising to decreasing. In high-income areas, the mortality rate of IS between 25 and 44 years old showed an increasing trend, while that of IS above 45 years old showed a decreasing trend. In the lower-middle income areas, the death rate of IS between 25 and 59 years old showed an increasing trend, and the death rate of IS over 60 years old showed a decreasing trend (Fig. 3a).

Longitudinal age curves show that IS mortality attributable to LPA increases gradually with age in all age groups and all study regions. It shows similar patterns of increase for male and female. There is a remarkable similarity in the longitudinal age curves of the global and 4 different World Bank Income levels regions, namely, the IS mortality over 80 years increases rapidly in low physical activity population compared with other age groups. The difference is that the IS mortality increases peaked at different levels, with the lowest in high-income regions (28.603% [95% CI: 26.484, 30.892]), especially among male in high-income regions (20.748% [95% CI: 19.049, 22.599]), and the highest in lower-middle income regions (120.663% [95% CI: 115.894, 125.627]) (Fig. 3b, Supplementary Table 6).

As shown in Fig. 3c and Supplementary Table 7, the global period RR for both male and female showed a downward trend, from 1.043% [95%CI: 1.030, 1.057] to 0.815% [95%CI: 0.804, 0.827]. Although the global period RR for male, female and the population as a whole differed slightly in 1990 (both male’s and female’s period RR were slightly higher than that of the whole population period RR), the situation crossed in 2000, and then the period RR for female IS declined more rapidly than that of male. The period RR in high-income regions also showed a much larger decline than the global, falling from 1.341% [95%CI: 1.293, 1.391] to 0.759% [95%CI: 0.725, 0.956]. Furthermore, we observe that the period RR in high-income regions stayed relatively steady from 2010 to 2019, with only a slight increase during the period. In the upper-middle income regions, the period RR for both male and female and the overall population showed a decreasing trend, from 1.093% [95%CI: 1.067, 1.120] to 0.734% [95%CI: 0.714, 0.754]. The period RR of the male, female, and overall period RR in the lower-middle income districts, however, exhibited the traits of first growing and then falling, with a small overall change amplitude.

As shown in Fig. 3d and Supplementary Table 8, the cohort RR for both male and female in the global showed a decreasing trend, from 2.298% [95%CI: 2.231, 2.367] in 1895 to 0.953% [95%CI: 0.776, 1.170] in 1994. Although the global cohort RR for female was higher than that for men in 1990, the situation crossed in 1945 and then showed a trend of higher cohort RR for male than for female. The cohort RR in high-income regions has shown a trend of first declining, then rising, and then slightly declining. In general, it declined from 6.233% [95%CI: 5.731, 6.779] in 1895 to a bottom of 0.876% [95%CI: 0.763, 1.005] in 1960, and then rose to 0.999% [95%CI: 0.427, 2.338]. The change pattern of cohort RR in upper-middle income regions is similar to that of the global cohort RR, which intersects in 1945. Before 1945, the cohort RR of female was higher than that of male, while after 1945, the opposite was true, and the cohort RR of male was higher. In lower-middle income regions, the cohort RR changed relatively slightly, slowly declining from 1895 to 1944 and gradually rising after 1945. The cohort RR in low-income areas maintained a relatively stable trend from 1895 to 1944, and showed an oscillating rise after 1945, but the rise was still small.

### Joinpoint

**Figure 4:**
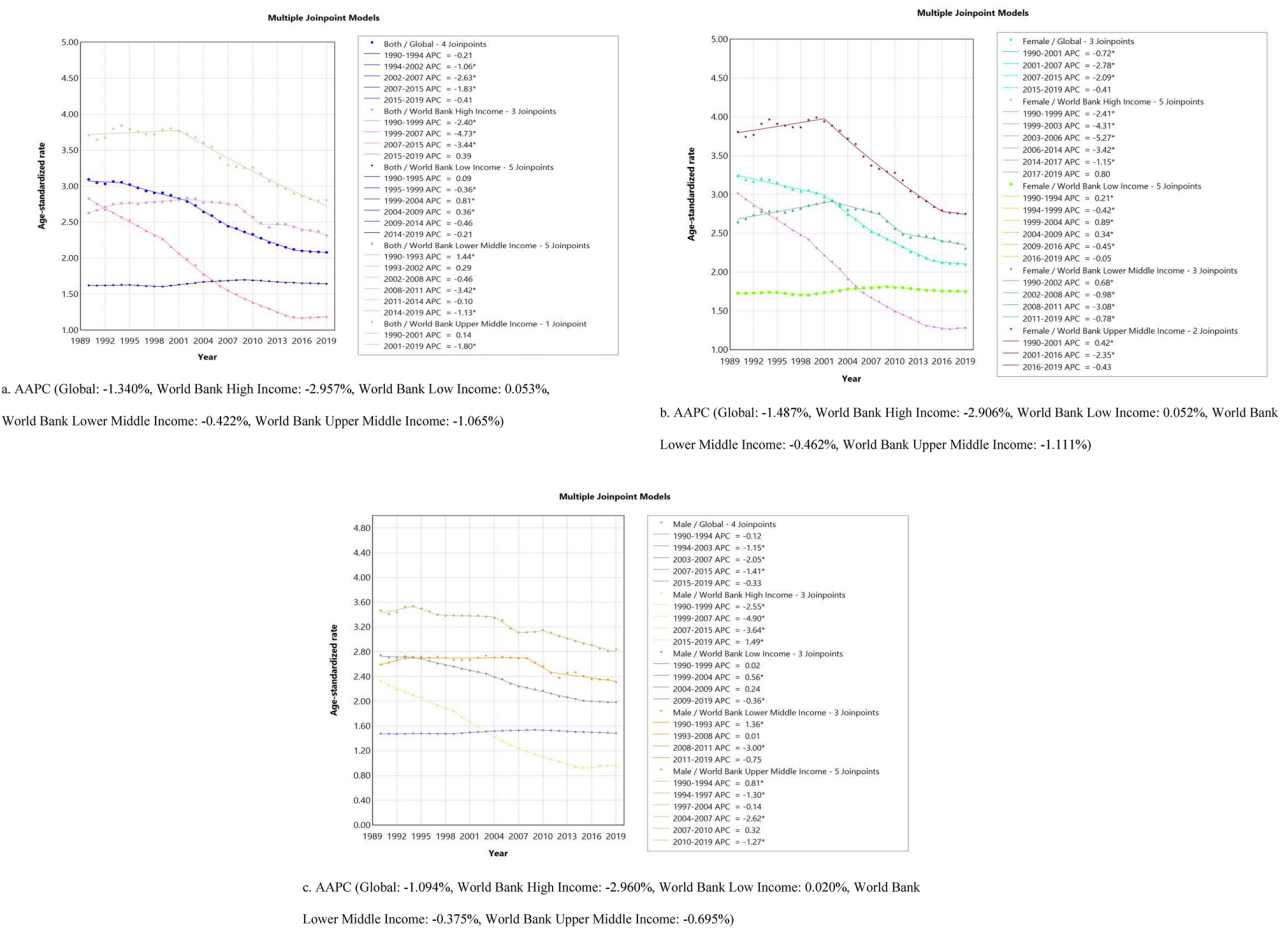
APC and AAPC in in sex-specific mortality of Ischemic stroke attributable to Low Physical Activity across Global and 4 World Bank Income Levels regions, 1990–2019.

Figure 4 (Joinpoint regression model) shows ASMR trends from 1990 to 2019 for IS (Ischemic stroke) attributable to LPA by sex and region. It is evident that, with the exception of low-income regions, death rates are falling globally and in each of the other three World Bank income levels regions for both genders.

In particular, the global IS ASMR attributed to LPA during the last 30 years was divided into 5 segments, 3 of which had a considerable reduction, and the most notable change took place between 2002 and 2007 (APC = − 2.628%). Over the full 1990–2019 period, the overall AAPC was − 1.34%.

The ASMR of IS attributable to LPA in high income regions is divided into four parts across the four World Bank income levels regions, with the most notable decline occurring between 1999 and 2007 (APC = − 4.726%). The region’s total AAPC is -2.957%. Similar to this, there is a notable decrease in the ASMR of IS related to LPA between 2001 and 2019 (APC = − 1.797%) in the upper middle income regions, which is divided into two categories. The region’s total AAPC is − 1.065%. The ASMR of IS attributable to LPA in lower middle income areas was divided into 6 segments, among which the trend of significant increase was from 1990 to 1993 (APC = 1.436), and the trend of most significant decrease was from 2008 to 2011 (APC = − 3.418%). The overall AAPC in the region is − 0.422%. Ultimately, the ASMR of IS related to LPA in low-income areas was divided into six segments, with the largest increase occurring from 1999 to 2004 (APC = 0.812%) and a notable decline from 1995 to 1999 (APC = − 0.356%). The region’s overall AAPC is 0.053% (Fig. 4a, Supplementary Tables 2, 3).

Furthermore, there exist notable disparities in ASMR patterns between genders on a global scale as well as within the four World Bank income level regions. There were four distinct segments to the global ASMR trend of female IS, with the most notable drop occurring between 2001 and 2007 (APC = − 2.785%). The region's total AAPC of ASMR for females is − 1.487%. There were five segments to the ASMR trend of male IS globally, with the most notable reduction occurring between 2003 and 2007 (APC = − 2.051%). For males in the region, the overall AAPC of ASMR is − 1.094%. In high income areas, the ASMR of female IS was divided into 6 segments, the reduction in this segment was greatest between 2003 and 2006 (APC = − 5.267%). The region's total AAPC of ASMR for females is − 2.906%. In contrast to female ASMR, male IS ASMR was divided into 4 segments, with the largest reduction occurring between 1999 and 2007 (APC = − 4.901%). Nonetheless, it displayed an increasing tendency (APC = 1.495%) from 2015 to 2019. For males in the area, the total AAPC of ASMR is − 2.960%. The ASMR of female IS in upper middle income regions was divided into three segments: 1990–2001 showed a significant increase trend (APC = 0.425%), and 2001–2016 showed a significant decrease trend (APC = − 2.355%). In this area, the total AAPC for ASMR in females was − 1.111%. Male IS's ASMR was broken down into six parts, and between 1990 and 1994, there was a noticeable rising tendency (APC = 0.81%). The biggest drop occurred between 2004 and 2007 (APC = − 2.622%). Among this area, the total AAPC for ASMR among males was − 0.695%. The ASMR of female IS in lower middle income areas was separated into four segments: the most significant declining trend occurred between 2008 and 2011 (APC = − 3.085%), while the increasing trend occurred between 1990 and 2002 (APC = 0.678%). In this area, the total AAPC for ASMR in females was − 0.462%. The male IS's ASMR was broken down into four segments, each of which displayed a noteworthy increase trend between 1990 and 1993 (APC = 1.36%) and a substantial decreasing trend between 2008 and 2011 (APC = − 3.002%). In this area, the male's total AAPC for ASMR was -0.375%. The female IS’s ASMR in low-income locations was broken down into six segments, with the rising trend peaking between 1999 and 2004 (APC = 0.892%). The greatest notable reduction occurred between 2009 and 2016 (APC = − 0.454%). Among this area, the overall AAPC for ASMR among females was 0.052%. There were four distinct segments in the ASMR of the male IS, and the data indicated a substantial increase trend from 1999 to 2004 (APC = 0.563%) and a significant decrease trend from 2009 to 2019 (APC = − 0.359%). In this region, the total AAPC for ASMR in males was 0.020% (Fig. 4a, Supplementary Table 3).

## Discussion

This study is the first to examine trends in IS-related mortality among individuals with LPA globally and in four distinct World Bank income levels regions over the previous three decades using an age-period-cohort model and Joinpoint regression analysis based on the GBD2019 database. Over the past three decades, there has been an overall annual increase in the number of IS deaths and crude mortality rates in the global LPA population; also, the IS death rate for females has consistently been higher than that of males. (Supplementary Figs. 1, 2) According to related studies, female tend to be less physically active than male, with an average inactivity rate of 31.7% for female compared to 23.4% for male^10^. The substantial sex difference between ASMR and crude mortality can be explained by this set of data. Figure 1 illustrates that the ASMR of IS caused by LPA is declining annually, and ASMR of females is larger than that of males, which is consistent with the gender disparity previously discussed (Fig. 1). However, The World Health Organization’s Global Status Report on Physical Activity 2022 estimates that 1.4 billion adults globally—or roughly 27.5% of the adult population—do not engage in enough physical activity, a statistic that has essentially stayed constant over time^15,16^. In the context of LPA data remaining largely unchanged and global aging, the mortality rate of IS is still declining. This is most likely due to population expansion, the dramatic improvements in health care brought about by economic development, and the fact that the decline in mortality in high-income regions far outweighs the continued deterioration in low—and middle-income countries^45^. Additionally, four distinct World Bank income level regions have high prevalence of this trait.

The ASMR of IS in the global LPA population generally showed a positive change, and the net drift value was less than 0, according to the results of the APC analysis's net drift and local drift. In contrast, the decline in ASMR was more significantly in women, with a downward trend in women of all ages. Globally, statistics reveal that females tend to be less physically active than males, with an average inactivity rate of 31.7% in females vs. 23.4% in males^10^. The underlying genetic and molecular mechanisms responsible for the positive effects of exercise training in both sexes remain to be fully elucidated^46^^.^ Nevertheless, this suggests a potential sex disparity of IS outcomes attributable to physical activity, warranting further investigation. We discovered that ASMR declined over time in the global LPA populations, which is consistent with the preceding findings, based on the age-specific mortality rates by period. It is important to note that this tendency is especially substantial in the older age group of 80 years and older (Fig. 2a). This may be due to targeted healthcare and preventive measures implemented in some regions to improve the quality of life of older IS groups. Because to a combination of factors including rising lifespan, falling birth rates, and a huge number of individuals reaching old age, the globe is currently witnessing unprecedented “population aging,” the most pervasive and dominant global demographic trend^10^. Given that IS is a prevalent illness among the elderly, it is crucial to take the age distribution of IS-related mortality into account when assessing IS-related death^28^. Accordingly, all regions should actively respond to the global aging trend and actively carry out appropriate physical exercise programs for chronic patients such as IS, especially middle-aged and elderly patients, so as to improve the quality of life of the global middle-aged and elderly IS population and effectively reduce the global burden of IS disease^28^.

We also looked at the ASMR of IS attributed to LPA across 4 different World Bank income levels regions and explored regional differences in IS ASMR across different income levels. Because the degree of economic development and income level of a region largely determine the medical level, medical quality, perfection of the medical security system and the accessibility of medical services in the region^8^. According to our research, high-income areas have had the lowest ASMR in 2019, at 1.182%, compared to the global (2.08%) and other regions with three different income levels: upper-medium (2.805%), lower-medium (2.31%) and low (1.644%) (Table 1). The highest ASMR, however, is seen in upper-middle income areas—second only to high-income areas—rather than low-income ones. Then followed by lower middle income areas. The huge population bases in these two regions, along with the variations in their historical and cultural backgrounds and customs, may be the cause of the higher ASMR in upper-middle income and lower-middle income regions. These nations have higher rates of LPA population, which leads to a comparatively large burden and death rate from IS. The net drift results explain the difference between the upper-middle income region and the lower-middle income region, where ASMR improved over the 30-year period, showing a significant downward trend, while the lower-middle income region saw a slight increase. In low-income areas, ASMR of IS attributed to LPA has been consistently low and has only increased slightly over the 30-year period. However, according to relevant studies, ASMR of all-cause IS has always shown a high trend in low-income areas^47^. This could be because the majority of low-income countries in the region are found in Africa, which makes up just 9% of the world’s population^48^. Because of the limited population base and the underdeveloped economy, the majority of people are forced to work manual labor in order to make ends meet. As a result, there are very few LPA. However, the degree of population aging in low-income areas is relatively low because women's education levels are generally lower there, especially in some African countries, and because local traditional concepts have a strong influence. Women also tend to have higher birth rates after marriage. The fact that low and middle income nations have few health resources and make insufficient investments in healthcare is another crucial factor. The ASMR of IS caused by LPA is lower in low-income communities, which might be partially explained by the aforementioned factors. There is an increasing trend because the net drift outcomes for low-income areas are not encouraging. Increasing the amount of basic healthcare and prevention should be the first line of defense. The second should be the creation and long-term implementation of efficient programs that promote physical activity in low-income communities.

Longitudinal analysis using an APC model provides insight into the effects of age, period, and cohort on IS-related mortality due to LPA, helping to identify causal factors and inform targeted prevention and treatment strategies^28^. The results of the age effects revealed a noteworthy trend: the ASMR of IS rose with age. This tendency is especially notable in the LPA population after the age of 60, increasing gradually from 60 to 79 years of age, and increasing rapidly from 80 years of age. This situation is largely attributed to the influence of aging population, especially in the middle and low income areas, the increase rate of ASMR in the age group over 80 is quite large, reflecting the relatively high degree of population aging in these two areas. Therefore, it is critical to develop effective prevention strategies for elderly patients with IS. The results of the period effects reflect the significant improvement in ASMR in high-income countries over the 30-year period, and combined with the Joinpoint regression model, there is a more favorable improvement in IS-related deaths in high-income regions than in other regions including the global and the 4 regions studied (AAPC: − 2.957%) (Fig. 4a). These findings imply that high income areas have made great efforts to lower the death rate of IS in the LPA population. These efforts have included raising health spending and investment, enhancing the standard of diagnosis and treatment, developing sensible physical activity plans, promoting healthy lifestyles, and implementing other policy preventive and rehabilitation measures. Over the course of the 30-year period, ASMR in lower-middle and low-income areas did not alter considerably (AAPC: − 0.422%, − 0.053%). These two places had the lowest ASMRs in 1990, but throughout the course of the following 30 years, the persistent efforts of high-income and upper-middle income areas further decreased the ASMR of IS in the LPA population in these two areas, making it even lower than that of lower-middle and low-income areas. It does, in part, reflect the pressing global public health issue that low-income and lower-middle areas ought to be actively addressing. Cohort effects show a clear trend that, globally, those born earlier with LPA have higher ASMR. This is especially true in high-income countries, where the higher mortality rate of IS among people with LPA may be due to the limitations of early medical technology, low public awareness, and unhealthy lifestyles. In lower-middle and low-income areas, on the other hand, the opposite is true, with those who were born into LPAs more recently having greater ASMR than those who were born earlier. This could be because low and lower-middle income communities have been experiencing economic growth recently, along with a slow but steady urbanization process that inevitably results in sedentary lifestyles. Another possible reason is that the burden of stroke due to air pollution and environmental risks, tobacco smoke, dietary risks, and hypertension is significantly higher in low and lower-middle income regions, where the impact of LPA is smaller, whereas in high-income regions the impact of LPA is much greater^49^.

## Limitations

This study has certain shortcomings. First, mortality estimates, APC analyses, and joinpoint regression analyses are significantly unclear when limited or poor quality raw data is used for low and lower-middle income regions or small-population countries^28^. Second, it can be challenging to identify whether a single cause of death contributed to an elderly person’s death because they frequently have several co-existing diseases^19^. In these situations, IS is regarded as a co-factor and the cause of death is frequently reported as a direct cause of death distinct from IS. This lowers the death rate linked to IS to some extent^50^. Third, the examination of the IS disease burden attributable to LPA was carried out globally and comprehensively across four distinct income levels areas; it did not delve deeper into the variations among individual countries within each region.

## Conclusion

This study highlights the significance of low physical activity as a modifiable risk factor for IS by illustrating changes in IS-related mortality attributable to LPA over the past three decades for the globe and four World Bank income levels regions. The findings indicate that the burden of IS-related deaths due to LPA is declining globally, with high-income areas seeing the greatest reduction. In some regions, such as low and lower-middle income regions, there is still a trend toward deteriorating. This requires strengthening international cooperation to formulate effective public health interventions and policies, jointly achieve public health goals, and promote the development of public health^8,51^. However, since the population ages faster, more research is needed to comprehend the factors that affect age groups, gender differences, and regional and national variances^8^. To enhance cardiovascular health on a national, regional, and international level, we ought to encourage the creation of more focused public health initiatives.

### Supplementary Information

Supplementary Information.

## Data Availability

The dataset generated for this study can be found in the GBD at http://ghdx.healthdata.org/gbd-results-tool.

## Abbreviations

LPA: Low physical activity
IS: Ischemic stroke
APC Analysis: Age-period-cohort analysis
ASMR: Age-standardized mortality rate
