# Supplementary material for: Age-period-cohort analysis of ischemic stroke deaths attributable to physical inactivity in different income regions
Source: Sci Rep. 2024 Mar 19;14:6547. doi: 10.1038/s41598-024-57309-2 (PMC10951293; doi:10.1038/s41598-024-57309-2)
Supplement: Supplementary file 1 — Supplementary Information. [file 41598_2024_57309_MOESM1_ESM.docx]

**
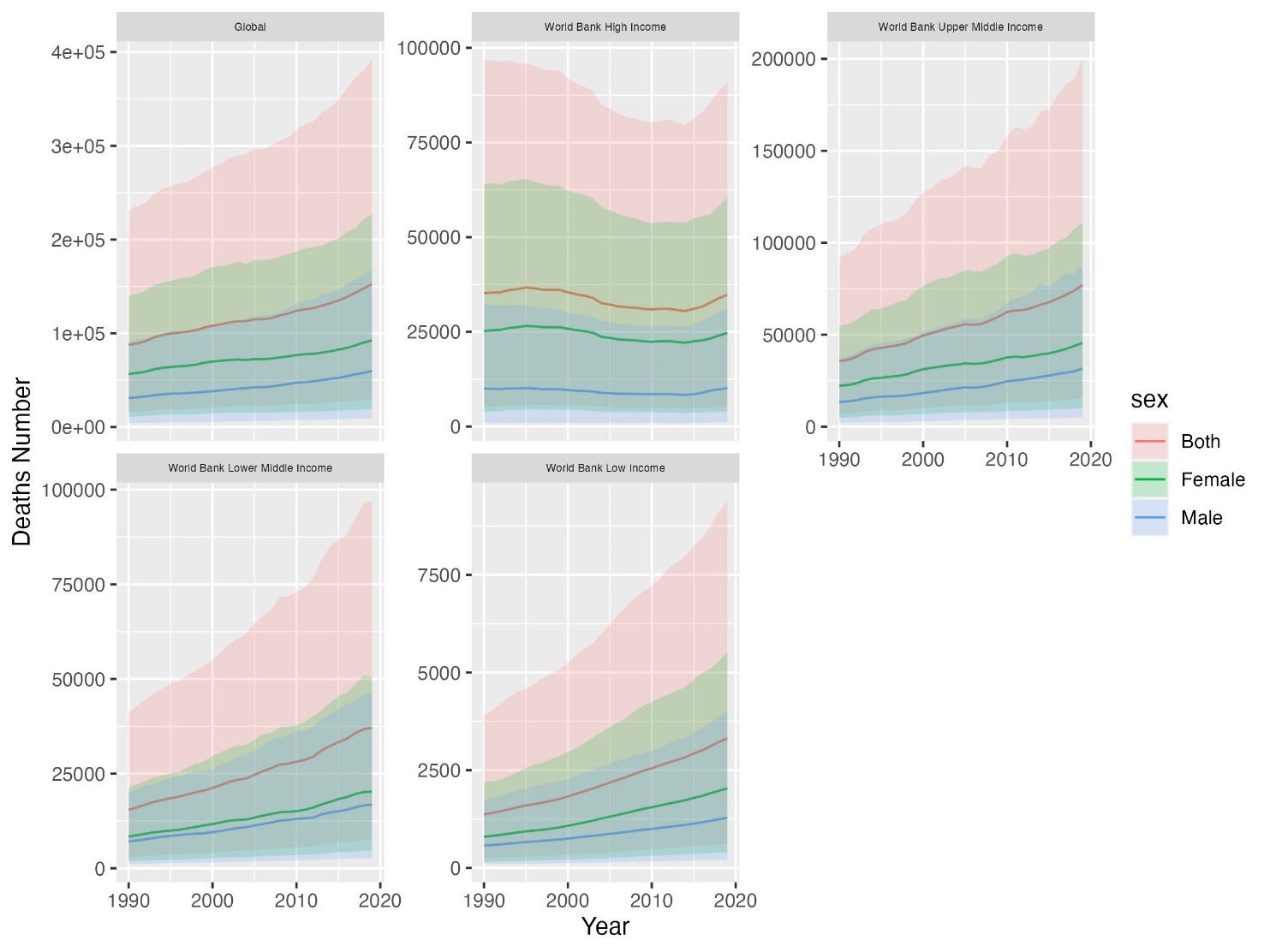
**

**Supplementary Figure 1.** Trends in deaths number from Ischemic Stroke attributable to Low Physical Activity globally and in four World Bank income level regions, 1990-2019

**
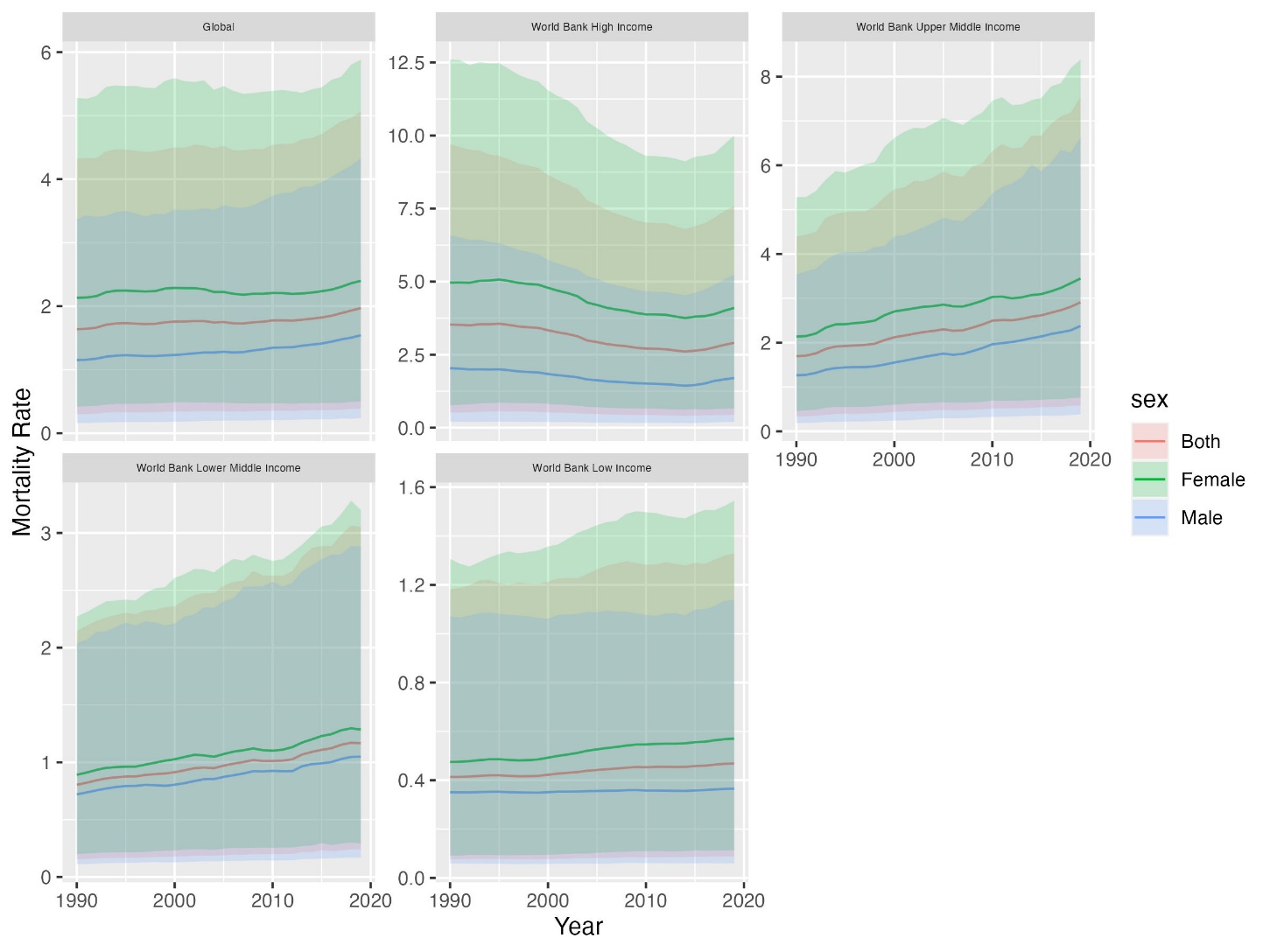
**

**Supplementary Figure 2.** Trends in crude death rate from Ischemic Stroke attributable to Low Physical Activity globally and in four World Bank income level regions, 1990-2019

**Supplementary Table 1. Fitting time trend results of Age-Period-Cohort analysis**

| Period | Rate | CI Lo | CI Hi | location | cause | measure | sex | rei | label | x |
| --- | --- | --- | --- | --- | --- | --- | --- | --- | --- | --- |
| 1,992.5 | 0.4504204 | 0.4385321 | 0.4626311 | Global | Ischemic stroke | Deaths | Both | Low physical activity | 1990 to 1994 | 1 |
| 1,997.5 | 0.4446878 | 0.4328648 | 0.4568336 | Global | Ischemic stroke | Deaths | Both | Low physical activity | 1995 to 1999 | 2 |
| 2,002.5 | 0.4316919 | 0.4198376 | 0.4438809 | Global | Ischemic stroke | Deaths | Both | Low physical activity | 2000 to 2004 | 3 |
| 2,007.5 | 0.3948427 | 0.3834423 | 0.4065821 | Global | Ischemic stroke | Deaths | Both | Low physical activity | 2005 to 2009 | 4 |
| 2,012.5 | 0.3657004 | 0.3544900 | 0.3772653 | Global | Ischemic stroke | Deaths | Both | Low physical activity | 2010 to 2014 | 5 |
| 2,017.5 | 0.3519098 | 0.3404336 | 0.3637729 | Global | Ischemic stroke | Deaths | Both | Low physical activity | 2015 to 2019 | 6 |
| 1,992.5 | 0.3323851 | 0.3025429 | 0.3651708 | World Bank High Income | Ischemic stroke | Deaths | Both | Low physical activity | 1990 to 1994 | 1 |
| 1,997.5 | 0.2915147 | 0.2650142 | 0.3206653 | World Bank High Income | Ischemic stroke | Deaths | Both | Low physical activity | 1995 to 1999 | 2 |
| 2,002.5 | 0.2478315 | 0.2244877 | 0.2736028 | World Bank High Income | Ischemic stroke | Deaths | Both | Low physical activity | 2000 to 2004 | 3 |
| 2,007.5 | 0.2060477 | 0.1856075 | 0.2287389 | World Bank High Income | Ischemic stroke | Deaths | Both | Low physical activity | 2005 to 2009 | 4 |
| 2,012.5 | 0.1855858 | 0.1660180 | 0.2074600 | World Bank High Income | Ischemic stroke | Deaths | Both | Low physical activity | 2010 to 2014 | 5 |
| 2,017.5 | 0.1882175 | 0.1670702 | 0.2120415 | World Bank High Income | Ischemic stroke | Deaths | Both | Low physical activity | 2015 to 2019 | 6 |
| 1,992.5 | 0.5669838 | 0.5401348 | 0.5951674 | World Bank Upper Middle Income | Ischemic stroke | Deaths | Both | Low physical activity | 1990 to 1994 | 1 |
| 1,997.5 | 0.5420117 | 0.5158945 | 0.5694511 | World Bank Upper Middle Income | Ischemic stroke | Deaths | Both | Low physical activity | 1995 to 1999 | 2 |
| 2,002.5 | 0.5186850 | 0.4926395 | 0.5461075 | World Bank Upper Middle Income | Ischemic stroke | Deaths | Both | Low physical activity | 2000 to 2004 | 3 |
| 2,007.5 | 0.4558099 | 0.4315004 | 0.4814890 | World Bank Upper Middle Income | Ischemic stroke | Deaths | Both | Low physical activity | 2005 to 2009 | 4 |
| 2,012.5 | 0.4126036 | 0.3890186 | 0.4376184 | World Bank Upper Middle Income | Ischemic stroke | Deaths | Both | Low physical activity | 2010 to 2014 | 5 |
| 2,017.5 | 0.3806004 | 0.3572607 | 0.4054648 | World Bank Upper Middle Income | Ischemic stroke | Deaths | Both | Low physical activity | 2015 to 2019 | 6 |
| 1,992.5 | 0.4021224 | 0.3832828 | 0.4218880 | World Bank Lower Middle Income | Ischemic stroke | Deaths | Both | Low physical activity | 1990 to 1994 | 1 |
| 1,997.5 | 0.4244843 | 0.4047320 | 0.4452007 | World Bank Lower Middle Income | Ischemic stroke | Deaths | Both | Low physical activity | 1995 to 1999 | 2 |
| 2,002.5 | 0.4428746 | 0.4218751 | 0.4649194 | World Bank Lower Middle Income | Ischemic stroke | Deaths | Both | Low physical activity | 2000 to 2004 | 3 |
| 2,007.5 | 0.4488528 | 0.4267949 | 0.4720508 | World Bank Lower Middle Income | Ischemic stroke | Deaths | Both | Low physical activity | 2005 to 2009 | 4 |
| 2,012.5 | 0.4252454 | 0.4033339 | 0.4483473 | World Bank Lower Middle Income | Ischemic stroke | Deaths | Both | Low physical activity | 2010 to 2014 | 5 |
| 2,017.5 | 0.4172502 | 0.3946263 | 0.4411711 | World Bank Lower Middle Income | Ischemic stroke | Deaths | Both | Low physical activity | 2015 to 2019 | 6 |
| 1,992.5 | 0.3367083 | 0.2925380 | 0.3875478 | World Bank Low Income | Ischemic stroke | Deaths | Both | Low physical activity | 1990 to 1994 | 1 |
| 1,997.5 | 0.3421381 | 0.2976829 | 0.3932322 | World Bank Low Income | Ischemic stroke | Deaths | Both | Low physical activity | 1995 to 1999 | 2 |
| 2,002.5 | 0.3525419 | 0.3061627 | 0.4059470 | World Bank Low Income | Ischemic stroke | Deaths | Both | Low physical activity | 2000 to 2004 | 3 |
| 2,007.5 | 0.3613769 | 0.3126327 | 0.4177210 | World Bank Low Income | Ischemic stroke | Deaths | Both | Low physical activity | 2005 to 2009 | 4 |
| 2,012.5 | 0.3595621 | 0.3093753 | 0.4178901 | World Bank Low Income | Ischemic stroke | Deaths | Both | Low physical activity | 2010 to 2014 | 5 |
| 2,017.5 | 0.3563803 | 0.3049374 | 0.4165018 | World Bank Low Income | Ischemic stroke | Deaths | Both | Low physical activity | 2015 to 2019 | 6 |
| 1,992.5 | 0.5182373 | 0.5001204 | 0.5370104 | Global | Ischemic stroke | Deaths | Male | Low physical activity | 1990 to 1994 | 1 |
| 1,997.5 | 0.5088440 | 0.4908464 | 0.5275015 | Global | Ischemic stroke | Deaths | Male | Low physical activity | 1995 to 1999 | 2 |
| 2,002.5 | 0.4911663 | 0.4731327 | 0.5098873 | Global | Ischemic stroke | Deaths | Male | Low physical activity | 2000 to 2004 | 3 |
| 2,007.5 | 0.4610836 | 0.4432070 | 0.4796813 | Global | Ischemic stroke | Deaths | Male | Low physical activity | 2005 to 2009 | 4 |
| 2,012.5 | 0.4370420 | 0.4189954 | 0.4558659 | Global | Ischemic stroke | Deaths | Male | Low physical activity | 2010 to 2014 | 5 |
| 2,017.5 | 0.4258587 | 0.4071402 | 0.4454379 | Global | Ischemic stroke | Deaths | Male | Low physical activity | 2015 to 2019 | 6 |
| 1,992.5 | 0.4086677 | 0.3700165 | 0.4513564 | World Bank High Income | Ischemic stroke | Deaths | Male | Low physical activity | 1990 to 1994 | 1 |
| 1,997.5 | 0.3662080 | 0.3308553 | 0.4053381 | World Bank High Income | Ischemic stroke | Deaths | Male | Low physical activity | 1995 to 1999 | 2 |
| 2,002.5 | 0.3145052 | 0.2826921 | 0.3498983 | World Bank High Income | Ischemic stroke | Deaths | Male | Low physical activity | 2000 to 2004 | 3 |
| 2,007.5 | 0.2641670 | 0.2357225 | 0.2960438 | World Bank High Income | Ischemic stroke | Deaths | Male | Low physical activity | 2005 to 2009 | 4 |
| 2,012.5 | 0.2343981 | 0.2073530 | 0.2649707 | World Bank High Income | Ischemic stroke | Deaths | Male | Low physical activity | 2010 to 2014 | 5 |
| 2,017.5 | 0.2401817 | 0.2105707 | 0.2739566 | World Bank High Income | Ischemic stroke | Deaths | Male | Low physical activity | 2015 to 2019 | 6 |
| 1,992.5 | 0.6549273 | 0.6232618 | 0.6882016 | World Bank Upper Middle Income | Ischemic stroke | Deaths | Male | Low physical activity | 1990 to 1994 | 1 |
| 1,997.5 | 0.6167562 | 0.5863382 | 0.6487522 | World Bank Upper Middle Income | Ischemic stroke | Deaths | Male | Low physical activity | 1995 to 1999 | 2 |
| 2,002.5 | 0.5866925 | 0.5564661 | 0.6185609 | World Bank Upper Middle Income | Ischemic stroke | Deaths | Male | Low physical activity | 2000 to 2004 | 3 |
| 2,007.5 | 0.5350759 | 0.5057283 | 0.5661265 | World Bank Upper Middle Income | Ischemic stroke | Deaths | Male | Low physical activity | 2005 to 2009 | 4 |
| 2,012.5 | 0.5102899 | 0.4802503 | 0.5422086 | World Bank Upper Middle Income | Ischemic stroke | Deaths | Male | Low physical activity | 2010 to 2014 | 5 |
| 2,017.5 | 0.4847944 | 0.4541910 | 0.5174598 | World Bank Upper Middle Income | Ischemic stroke | Deaths | Male | Low physical activity | 2015 to 2019 | 6 |
| 1,992.5 | 0.4631743 | 0.4346424 | 0.4935792 | World Bank Lower Middle Income | Ischemic stroke | Deaths | Male | Low physical activity | 1990 to 1994 | 1 |
| 1,997.5 | 0.4876746 | 0.4576947 | 0.5196183 | World Bank Lower Middle Income | Ischemic stroke | Deaths | Male | Low physical activity | 1995 to 1999 | 2 |
| 2,002.5 | 0.5000664 | 0.4685455 | 0.5337079 | World Bank Lower Middle Income | Ischemic stroke | Deaths | Male | Low physical activity | 2000 to 2004 | 3 |
| 2,007.5 | 0.5150521 | 0.4812313 | 0.5512498 | World Bank Lower Middle Income | Ischemic stroke | Deaths | Male | Low physical activity | 2005 to 2009 | 4 |
| 2,012.5 | 0.4913432 | 0.4573459 | 0.5278679 | World Bank Lower Middle Income | Ischemic stroke | Deaths | Male | Low physical activity | 2010 to 2014 | 5 |
| 2,017.5 | 0.4799565 | 0.4449164 | 0.5177562 | World Bank Lower Middle Income | Ischemic stroke | Deaths | Male | Low physical activity | 2015 to 2019 | 6 |
| 1,992.5 | 0.3229409 | 0.2624985 | 0.3973006 | World Bank Low Income | Ischemic stroke | Deaths | Male | Low physical activity | 1990 to 1994 | 1 |
| 1,997.5 | 0.3320841 | 0.2702781 | 0.4080238 | World Bank Low Income | Ischemic stroke | Deaths | Male | Low physical activity | 1995 to 1999 | 2 |
| 2,002.5 | 0.3428489 | 0.2778134 | 0.4231090 | World Bank Low Income | Ischemic stroke | Deaths | Male | Low physical activity | 2000 to 2004 | 3 |
| 2,007.5 | 0.3528418 | 0.2838399 | 0.4386183 | World Bank Low Income | Ischemic stroke | Deaths | Male | Low physical activity | 2005 to 2009 | 4 |
| 2,012.5 | 0.3524951 | 0.2809194 | 0.4423077 | World Bank Low Income | Ischemic stroke | Deaths | Male | Low physical activity | 2010 to 2014 | 5 |
| 2,017.5 | 0.3504996 | 0.2768442 | 0.4437513 | World Bank Low Income | Ischemic stroke | Deaths | Male | Low physical activity | 2015 to 2019 | 6 |
| 1,992.5 | 0.3827738 | 0.3674031 | 0.3987875 | Global | Ischemic stroke | Deaths | Female | Low physical activity | 1990 to 1994 | 1 |
| 1,997.5 | 0.3755539 | 0.3604159 | 0.3913278 | Global | Ischemic stroke | Deaths | Female | Low physical activity | 1995 to 1999 | 2 |
| 2,002.5 | 0.3633065 | 0.3482690 | 0.3789933 | Global | Ischemic stroke | Deaths | Female | Low physical activity | 2000 to 2004 | 3 |
| 2,007.5 | 0.3256620 | 0.3115770 | 0.3403838 | Global | Ischemic stroke | Deaths | Female | Low physical activity | 2005 to 2009 | 4 |
| 2,012.5 | 0.2965590 | 0.2830161 | 0.3107499 | Global | Ischemic stroke | Deaths | Female | Low physical activity | 2010 to 2014 | 5 |
| 2,017.5 | 0.2831481 | 0.2694391 | 0.2975546 | Global | Ischemic stroke | Deaths | Female | Low physical activity | 2015 to 2019 | 6 |
| 1,992.5 | 0.2515090 | 0.2218471 | 0.2851368 | World Bank High Income | Ischemic stroke | Deaths | Female | Low physical activity | 1990 to 1994 | 1 |
| 1,997.5 | 0.2168289 | 0.1911180 | 0.2459986 | World Bank High Income | Ischemic stroke | Deaths | Female | Low physical activity | 1995 to 1999 | 2 |
| 2,002.5 | 0.1823032 | 0.1601510 | 0.2075196 | World Bank High Income | Ischemic stroke | Deaths | Female | Low physical activity | 2000 to 2004 | 3 |
| 2,007.5 | 0.1502455 | 0.1312532 | 0.1719861 | World Bank High Income | Ischemic stroke | Deaths | Female | Low physical activity | 2005 to 2009 | 4 |
| 2,012.5 | 0.1359787 | 0.1179160 | 0.1568083 | World Bank High Income | Ischemic stroke | Deaths | Female | Low physical activity | 2010 to 2014 | 5 |
| 2,017.5 | 0.1378570 | 0.1185089 | 0.1603641 | World Bank High Income | Ischemic stroke | Deaths | Female | Low physical activity | 2015 to 2019 | 6 |
| 1,992.5 | 0.4823423 | 0.4458847 | 0.5217809 | World Bank Upper Middle Income | Ischemic stroke | Deaths | Female | Low physical activity | 1990 to 1994 | 1 |
| 1,997.5 | 0.4586886 | 0.4234653 | 0.4968416 | World Bank Upper Middle Income | Ischemic stroke | Deaths | Female | Low physical activity | 1995 to 1999 | 2 |
| 2,002.5 | 0.4348627 | 0.4001747 | 0.4725577 | World Bank Upper Middle Income | Ischemic stroke | Deaths | Female | Low physical activity | 2000 to 2004 | 3 |
| 2,007.5 | 0.3687587 | 0.3376454 | 0.4027390 | World Bank Upper Middle Income | Ischemic stroke | Deaths | Female | Low physical activity | 2005 to 2009 | 4 |
| 2,012.5 | 0.3192309 | 0.2904688 | 0.3508409 | World Bank Upper Middle Income | Ischemic stroke | Deaths | Female | Low physical activity | 2010 to 2014 | 5 |
| 2,017.5 | 0.2864803 | 0.2588501 | 0.3170598 | World Bank Upper Middle Income | Ischemic stroke | Deaths | Female | Low physical activity | 2015 to 2019 | 6 |
| 1,992.5 | 0.3384125 | 0.3144318 | 0.3642221 | World Bank Lower Middle Income | Ischemic stroke | Deaths | Female | Low physical activity | 1990 to 1994 | 1 |
| 1,997.5 | 0.3581100 | 0.3330122 | 0.3850994 | World Bank Lower Middle Income | Ischemic stroke | Deaths | Female | Low physical activity | 1995 to 1999 | 2 |
| 2,002.5 | 0.3794400 | 0.3525290 | 0.4084052 | World Bank Lower Middle Income | Ischemic stroke | Deaths | Female | Low physical activity | 2000 to 2004 | 3 |
| 2,007.5 | 0.3796543 | 0.3519379 | 0.4095536 | World Bank Lower Middle Income | Ischemic stroke | Deaths | Female | Low physical activity | 2005 to 2009 | 4 |
| 2,012.5 | 0.3576742 | 0.3304875 | 0.3870975 | World Bank Lower Middle Income | Ischemic stroke | Deaths | Female | Low physical activity | 2010 to 2014 | 5 |
| 2,017.5 | 0.3523949 | 0.3243532 | 0.3828609 | World Bank Lower Middle Income | Ischemic stroke | Deaths | Female | Low physical activity | 2015 to 2019 | 6 |
| 1,992.5 | 0.3530118 | 0.2913515 | 0.4277215 | World Bank Low Income | Ischemic stroke | Deaths | Female | Low physical activity | 1990 to 1994 | 1 |
| 1,997.5 | 0.3539348 | 0.2929157 | 0.4276653 | World Bank Low Income | Ischemic stroke | Deaths | Female | Low physical activity | 1995 to 1999 | 2 |
| 2,002.5 | 0.3623492 | 0.2994082 | 0.4385214 | World Bank Low Income | Ischemic stroke | Deaths | Female | Low physical activity | 2000 to 2004 | 3 |
| 2,007.5 | 0.3710245 | 0.3052985 | 0.4509003 | World Bank Low Income | Ischemic stroke | Deaths | Female | Low physical activity | 2005 to 2009 | 4 |
| 2,012.5 | 0.3678060 | 0.3006603 | 0.4499473 | World Bank Low Income | Ischemic stroke | Deaths | Female | Low physical activity | 2010 to 2014 | 5 |
| 2,017.5 | 0.3635863 | 0.2951168 | 0.4479415 | World Bank Low Income | Ischemic stroke | Deaths | Female | Low physical activity | 2015 to 2019 | 6 |

**Supplementary Table 2.APC results of Joinpoint regression analysis**

| sex | location | Model | Segment | Segment Start | Segment End | APC | APC 95% LCL | APC 95% UCL | APC Significant |
| --- | --- | --- | --- | --- | --- | --- | --- | --- | --- |
| Both | Global | 4 | 0 | 1990 | 1994 | -0.2104 | -0.5774 | 0.4852 | 0 |
| Both | Global | 4 | 1 | 1994 | 2002 | -1.0597 | -1.3085 | -0.91 | 1 |
| Both | Global | 4 | 2 | 2002 | 2007 | -2.6276 | -3.1659 | -2.2992 | 1 |
| Both | Global | 4 | 3 | 2007 | 2015 | -1.8339 | -2.054 | -1.4877 | 1 |
| Both | Global | 4 | 4 | 2015 | 2019 | -0.4053 | -0.8219 | 0.4182 | 0 |
| Female | Global | 3 | 0 | 1990 | 2001 | -0.7215 | -0.8517 | -0.5886 | 1 |
| Female | Global | 3 | 1 | 2001 | 2007 | -2.7848 | -3.369 | -2.4307 | 1 |
| Female | Global | 3 | 2 | 2007 | 2015 | -2.0869 | -2.3577 | -1.629 | 1 |
| Female | Global | 3 | 3 | 2015 | 2019 | -0.4136 | -0.9111 | 0.5112 | 0 |
| Male | Global | 4 | 0 | 1990 | 1994 | -0.1247 | -0.4469 | 0.3854 | 0 |
| Male | Global | 4 | 1 | 1994 | 2003 | -1.1536 | -1.2804 | -1.017 | 1 |
| Male | Global | 4 | 2 | 2003 | 2007 | -2.0506 | -2.4013 | -1.7197 | 1 |
| Male | Global | 4 | 3 | 2007 | 2015 | -1.41 | -1.558 | -1.1498 | 1 |
| Male | Global | 4 | 4 | 2015 | 2019 | -0.3276 | -0.6496 | 0.2351 | 0 |
| Both | World Bank High Income | 3 | 0 | 1990 | 1999 | -2.3975 | -2.5609 | -2.2263 | 1 |
| Both | World Bank High Income | 3 | 1 | 1999 | 2007 | -4.7258 | -5.0057 | -4.5305 | 1 |
| Both | World Bank High Income | 3 | 2 | 2007 | 2015 | -3.4429 | -3.6368 | -3.2005 | 1 |
| Both | World Bank High Income | 3 | 3 | 2015 | 2019 | 0.3908 | -0.0371 | 0.8156 | 0 |
| Female | World Bank High Income | 5 | 0 | 1990 | 1999 | -2.4067 | -2.7157 | -2.1833 | 1 |
| Female | World Bank High Income | 5 | 1 | 1999 | 2003 | -4.3081 | -4.6567 | -2.1144 | 1 |
| Female | World Bank High Income | 5 | 2 | 2003 | 2006 | -5.2672 | -5.6042 | -3.7243 | 1 |
| Female | World Bank High Income | 5 | 3 | 2006 | 2014 | -3.4225 | -5.0934 | -3.1772 | 1 |
| Female | World Bank High Income | 5 | 4 | 2014 | 2017 | -1.1491 | -3.422 | -0.8315 | 1 |
| Female | World Bank High Income | 5 | 5 | 2017 | 2019 | 0.7966 | -0.2469 | 1.4779 | 0 |
| Male | World Bank High Income | 3 | 0 | 1990 | 1999 | -2.5492 | -2.7492 | -2.3443 | 1 |
| Male | World Bank High Income | 3 | 1 | 1999 | 2007 | -4.9008 | -5.2979 | -4.6612 | 1 |
| Male | World Bank High Income | 3 | 2 | 2007 | 2015 | -3.6364 | -3.8541 | -3.3225 | 1 |
| Male | World Bank High Income | 3 | 3 | 2015 | 2019 | 1.495 | 1.0098 | 1.9518 | 1 |
| Both | World Bank Low Income | 5 | 0 | 1990 | 1995 | 0.091 | -0.0106 | 0.2485 | 0 |
| Both | World Bank Low Income | 5 | 1 | 1995 | 1999 | -0.3562 | -0.6077 | -0.1921 | 1 |
| Both | World Bank Low Income | 5 | 2 | 1999 | 2004 | 0.8124 | 0.6506 | 1.0736 | 1 |
| Both | World Bank Low Income | 5 | 3 | 2004 | 2009 | 0.3626 | 0.2071 | 0.5655 | 1 |
| Both | World Bank Low Income | 5 | 4 | 2009 | 2014 | -0.4593 | -0.6499 | 0.1278 | 0 |
| Both | World Bank Low Income | 5 | 5 | 2014 | 2019 | -0.2116 | -0.3347 | 0.0478 | 0 |
| Female | World Bank Low Income | 5 | 0 | 1990 | 1994 | 0.2076 | 0.0725 | 0.3729 | 1 |
| Female | World Bank Low Income | 5 | 1 | 1994 | 1999 | -0.4231 | -0.5526 | -0.3225 | 1 |
| Female | World Bank Low Income | 5 | 2 | 1999 | 2004 | 0.8924 | 0.7908 | 1.0295 | 1 |
| Female | World Bank Low Income | 5 | 3 | 2004 | 2009 | 0.3411 | 0.1855 | 0.4501 | 1 |
| Female | World Bank Low Income | 5 | 4 | 2009 | 2016 | -0.4542 | -0.6219 | -0.3899 | 1 |
| Female | World Bank Low Income | 5 | 5 | 2016 | 2019 | -0.053 | -0.2638 | 0.2278 | 0 |
| Male | World Bank Low Income | 3 | 0 | 1990 | 1999 | 0.0215 | -0.0514 | 0.0772 | 0 |
| Male | World Bank Low Income | 3 | 1 | 1999 | 2004 | 0.5627 | 0.3967 | 0.8072 | 1 |
| Male | World Bank Low Income | 3 | 2 | 2004 | 2009 | 0.2378 | -0.1216 | 0.3777 | 0 |
| Male | World Bank Low Income | 3 | 3 | 2009 | 2019 | -0.3589 | -0.4151 | -0.3102 | 1 |
| Both | World Bank Lower Middle Income | 5 | 0 | 1990 | 1993 | 1.4357 | 0.8696 | 2.5367 | 1 |
| Both | World Bank Lower Middle Income | 5 | 1 | 1993 | 2002 | 0.2942 | -0.0205 | 0.4496 | 0 |
| Both | World Bank Lower Middle Income | 5 | 2 | 2002 | 2008 | -0.462 | -0.7833 | 0.4553 | 0 |
| Both | World Bank Lower Middle Income | 5 | 3 | 2008 | 2011 | -3.4177 | -3.8431 | -0.4802 | 1 |
| Both | World Bank Lower Middle Income | 5 | 4 | 2011 | 2014 | -0.1027 | -3.0118 | 0.3703 | 0 |
| Both | World Bank Lower Middle Income | 5 | 5 | 2014 | 2019 | -1.1285 | -1.8199 | -0.7687 | 1 |
| Female | World Bank Lower Middle Income | 3 | 0 | 1990 | 2002 | 0.6784 | 0.5366 | 0.8549 | 1 |
| Female | World Bank Lower Middle Income | 3 | 1 | 2002 | 2008 | -0.9791 | -1.3467 | -0.3588 | 1 |
| Female | World Bank Lower Middle Income | 3 | 2 | 2008 | 2011 | -3.085 | -3.5803 | -2.0346 | 1 |
| Female | World Bank Lower Middle Income | 3 | 3 | 2011 | 2019 | -0.7761 | -1.005 | -0.3768 | 1 |
| Male | World Bank Lower Middle Income | 3 | 0 | 1990 | 1993 | 1.3599 | 0.1805 | 3.2426 | 1 |
| Male | World Bank Lower Middle Income | 3 | 1 | 1993 | 2008 | 0.0125 | -2.1681 | 0.1293 | 0 |
| Male | World Bank Lower Middle Income | 3 | 2 | 2008 | 2011 | -3.0016 | -3.4915 | -1.1138 | 1 |
| Male | World Bank Lower Middle Income | 3 | 3 | 2011 | 2019 | -0.7453 | -1.1121 | 0.0185 | 0 |
| Both | World Bank Upper Middle Income | 1 | 0 | 1990 | 2001 | 0.1431 | -0.1561 | 0.5129 | 0 |
| Both | World Bank Upper Middle Income | 1 | 1 | 2001 | 2019 | -1.7965 | -1.9621 | -1.6533 | 1 |
| Female | World Bank Upper Middle Income | 2 | 0 | 1990 | 2001 | 0.4246 | 0.1284 | 0.7251 | 1 |
| Female | World Bank Upper Middle Income | 2 | 1 | 2001 | 2016 | -2.3549 | -2.818 | -2.1373 | 1 |
| Female | World Bank Upper Middle Income | 2 | 2 | 2016 | 2019 | -0.4326 | -2.086 | 1.3987 | 0 |
| Male | World Bank Upper Middle Income | 5 | 0 | 1990 | 1994 | 0.8103 | 0.4207 | 1.4953 | 1 |
| Male | World Bank Upper Middle Income | 5 | 1 | 1994 | 1997 | -1.3049 | -1.6949 | -0.6214 | 1 |
| Male | World Bank Upper Middle Income | 5 | 2 | 1997 | 2004 | -0.1404 | -0.2911 | 0.3763 | 0 |
| Male | World Bank Upper Middle Income | 5 | 3 | 2004 | 2007 | -2.6223 | -3.0551 | -1.8932 | 1 |
| Male | World Bank Upper Middle Income | 5 | 4 | 2007 | 2010 | 0.3205 | -0.3388 | 0.7287 | 0 |
| Male | World Bank Upper Middle Income | 5 | 5 | 2010 | 2019 | -1.2726 | -1.4627 | -1.1436 | 1 |

**Supplementary Table 3. AAPC results of Joinpoint regression analysis**

| sex | location | Joinpoint Model | AAPC Index | Start Obs | End Obs | AAPC | AAPC C.I. Low | AAPC C.I. High | Statistically Significant (0=No 1=Yes) |
| --- | --- | --- | --- | --- | --- | --- | --- | --- | --- |
| Both | Global | 4 | Full Range | 1990 | 2019 | -1.3396 | -1.3819 | -1.2897 | 1 |
| Female | Global | 3 | Full Range | 1990 | 2019 | -1.4868 | -1.5349 | -1.4418 | 1 |
| Male | Global | 4 | Full Range | 1990 | 2019 | -1.094 | -1.1283 | -1.0557 | 1 |
| Both | World Bank High Income | 3 | Full Range | 1990 | 2019 | -2.9567 | -3.0024 | -2.9105 | 1 |
| Female | World Bank High Income | 5 | Full Range | 1990 | 2019 | -2.9057 | -2.9565 | -2.8682 | 1 |
| Male | World Bank High Income | 3 | Full Range | 1990 | 2019 | -2.9601 | -3.0141 | -2.9071 | 1 |
| Both | World Bank Low Income | 5 | Full Range | 1990 | 2019 | 0.0525 | 0.0362 | 0.0709 | 1 |
| Female | World Bank Low Income | 5 | Full Range | 1990 | 2019 | 0.052 | 0.0358 | 0.0678 | 1 |
| Male | World Bank Low Income | 3 | Full Range | 1990 | 2019 | 0.0204 | 0.0065 | 0.0333 | 1 |
| Both | World Bank Lower Middle Income | 5 | Full Range | 1990 | 2019 | -0.4223 | -0.4766 | -0.3651 | 1 |
| Female | World Bank Lower Middle Income | 3 | Full Range | 1990 | 2019 | -0.4618 | -0.5128 | -0.4038 | 1 |
| Male | World Bank Lower Middle Income | 3 | Full Range | 1990 | 2019 | -0.3747 | -0.4682 | -0.2686 | 1 |
| Both | World Bank Upper Middle Income | 1 | Full Range | 1990 | 2019 | -1.0652 | -1.149 | -0.9707 | 1 |
| Female | World Bank Upper Middle Income | 2 | Full Range | 1990 | 2019 | -1.1105 | -1.235 | -1.0189 | 1 |
| Male | World Bank Upper Middle Income | 5 | Full Range | 1990 | 2019 | -0.6953 | -0.7389 | -0.6501 | 1 |

**Supplementary Table 4. Net Drift results of Age-Period-Cohort analysis**

| Net Drift (%/year) | CILo | CIHi | location | cause | measure | sex | rei |
| --- | --- | --- | --- | --- | --- | --- | --- |
| -1.0854552 | -1.16790846 | -1.0029331 | Global | Ischemic stroke | Deaths | Both | Low physical activity |
| -2.4733793 | -2.75910875 | -2.1868102 | World Bank High Income | Ischemic stroke | Deaths | Both | Low physical activity |
| -1.6662620 | -1.82518279 | -1.5070840 | World Bank Upper Middle Income | Ischemic stroke | Deaths | Both | Low physical activity |
| 0.1163136 | -0.02594593 | 0.2587756 | World Bank Lower Middle Income | Ischemic stroke | Deaths | Both | Low physical activity |
| 0.2618720 | -0.13489993 | 0.6602204 | World Bank Low Income | Ischemic stroke | Deaths | Both | Low physical activity |
| -0.8541413 | -0.96894805 | -0.7392014 | Global | Ischemic stroke | Deaths | Male | Low physical activity |
| -2.3549668 | -2.68277935 | -2.0260499 | World Bank High Income | Ischemic stroke | Deaths | Male | Low physical activity |
| -1.2292810 | -1.39755457 | -1.0607202 | World Bank Upper Middle Income | Ischemic stroke | Deaths | Male | Low physical activity |
| 0.1314985 | -0.06582297 | 0.3292097 | World Bank Lower Middle Income | Ischemic stroke | Deaths | Male | Low physical activity |
| 0.3532666 | -0.25799722 | 0.9682766 | World Bank Low Income | Ischemic stroke | Deaths | Male | Low physical activity |
| -1.3199120 | -1.43963068 | -1.2000479 | Global | Ischemic stroke | Deaths | Female | Low physical activity |
| -2.5940679 | -2.93922272 | -2.2476856 | World Bank High Income | Ischemic stroke | Deaths | Female | Low physical activity |
| -2.1799900 | -2.42701071 | -1.9323439 | World Bank Upper Middle Income | Ischemic stroke | Deaths | Female | Low physical activity |
| 0.1139768 | -0.09239660 | 0.3207766 | World Bank Lower Middle Income | Ischemic stroke | Deaths | Female | Low physical activity |
| 0.1638859 | -0.35783785 | 0.6883414 | World Bank Low Income | Ischemic stroke | Deaths | Female | Low physical activity |

**Supplementary Table 5. Local Drift results of Age-Period-Cohort analysis**

| Age | Mean Percent Change per Calendar Year | CILo | CIHi | location | cause | measure | sex | rei | label | x |
| --- | --- | --- | --- | --- | --- | --- | --- | --- | --- | --- |
| 27.5 | 0.244126767 | -0.420326209 | 0.9130133565 | Global | Ischemic stroke | Deaths | Both | Low physical activity | 25 to 29 | 1 |
| 32.5 | 0.289255453 | -0.163626666 | 0.7441919564 | Global | Ischemic stroke | Deaths | Both | Low physical activity | 30 to 34 | 2 |
| 37.5 | 0.089006471 | -0.253626456 | 0.4328163561 | Global | Ischemic stroke | Deaths | Both | Low physical activity | 35 to 39 | 3 |
| 42.5 | -0.242564627 | -0.508378771 | 0.0239596983 | Global | Ischemic stroke | Deaths | Both | Low physical activity | 40 to 44 | 4 |
| 47.5 | -0.559445840 | -0.762968814 | -0.3555054660 | Global | Ischemic stroke | Deaths | Both | Low physical activity | 45 to 49 | 5 |
| 52.5 | -0.892679542 | -1.048333598 | -0.7367806379 | Global | Ischemic stroke | Deaths | Both | Low physical activity | 50 to 54 | 6 |
| 57.5 | -1.250652007 | -1.370120699 | -1.1310386053 | Global | Ischemic stroke | Deaths | Both | Low physical activity | 55 to 59 | 7 |
| 62.5 | -1.557265929 | -1.644681053 | -1.4697731134 | Global | Ischemic stroke | Deaths | Both | Low physical activity | 60 to 64 | 8 |
| 67.5 | -1.737342912 | -1.806032178 | -1.6686055973 | Global | Ischemic stroke | Deaths | Both | Low physical activity | 65 to 69 | 9 |
| 72.5 | -1.717106720 | -1.773499763 | -1.6606813006 | Global | Ischemic stroke | Deaths | Both | Low physical activity | 70 to 74 | 10 |
| 77.5 | -1.655470689 | -1.702020490 | -1.6088988437 | Global | Ischemic stroke | Deaths | Both | Low physical activity | 75 to 79 | 11 |
| 82.5 | -1.658730569 | -1.696126005 | -1.6213209067 | Global | Ischemic stroke | Deaths | Both | Low physical activity | 80 to 84 | 12 |
| 87.5 | -1.652487566 | -1.694921931 | -1.6100348843 | Global | Ischemic stroke | Deaths | Both | Low physical activity | 85 to 89 | 13 |
| 92.5 | -1.671103888 | -1.742440844 | -1.5997151386 | Global | Ischemic stroke | Deaths | Both | Low physical activity | 90 to 94 | 14 |
| 27.5 | 0.818792485 | -1.846024074 | 3.5559570794 | World Bank High Income | Ischemic stroke | Deaths | Both | Low physical activity | 25 to 29 | 1 |
| 32.5 | 1.533819988 | -0.106835283 | 3.2014215454 | World Bank High Income | Ischemic stroke | Deaths | Both | Low physical activity | 30 to 34 | 2 |
| 37.5 | 1.172202284 | -0.001717110 | 2.3599027807 | World Bank High Income | Ischemic stroke | Deaths | Both | Low physical activity | 35 to 39 | 3 |
| 42.5 | 0.301788405 | -0.611812398 | 1.2237872523 | World Bank High Income | Ischemic stroke | Deaths | Both | Low physical activity | 40 to 44 | 4 |
| 47.5 | -0.839830128 | -1.562678006 | -0.1116742122 | World Bank High Income | Ischemic stroke | Deaths | Both | Low physical activity | 45 to 49 | 5 |
| 52.5 | -1.805800394 | -2.376289135 | -1.2319778578 | World Bank High Income | Ischemic stroke | Deaths | Both | Low physical activity | 50 to 54 | 6 |
| 57.5 | -2.828727359 | -3.271551926 | -2.3838755331 | World Bank High Income | Ischemic stroke | Deaths | Both | Low physical activity | 55 to 59 | 7 |
| 62.5 | -3.765925775 | -4.092434851 | -3.4383051275 | World Bank High Income | Ischemic stroke | Deaths | Both | Low physical activity | 60 to 64 | 8 |
| 67.5 | -4.569295771 | -4.815947111 | -4.3220052812 | World Bank High Income | Ischemic stroke | Deaths | Both | Low physical activity | 65 to 69 | 9 |
| 72.5 | -4.840092846 | -5.025372944 | -4.6544512962 | World Bank High Income | Ischemic stroke | Deaths | Both | Low physical activity | 70 to 74 | 10 |
| 77.5 | -4.748070138 | -4.884899594 | -4.6110438437 | World Bank High Income | Ischemic stroke | Deaths | Both | Low physical activity | 75 to 79 | 11 |
| 82.5 | -4.247595731 | -4.337137059 | -4.1579705914 | World Bank High Income | Ischemic stroke | Deaths | Both | Low physical activity | 80 to 84 | 12 |
| 87.5 | -3.528358221 | -3.609969456 | -3.4466778880 | World Bank High Income | Ischemic stroke | Deaths | Both | Low physical activity | 85 to 89 | 13 |
| 92.5 | -2.749643450 | -2.871639376 | -2.6274942945 | World Bank High Income | Ischemic stroke | Deaths | Both | Low physical activity | 90 to 94 | 14 |
| 27.5 | -0.819245897 | -2.263022321 | 0.6458580781 | World Bank Upper Middle Income | Ischemic stroke | Deaths | Both | Low physical activity | 25 to 29 | 1 |
| 32.5 | -0.826383391 | -1.758605672 | 0.1146848383 | World Bank Upper Middle Income | Ischemic stroke | Deaths | Both | Low physical activity | 30 to 34 | 2 |
| 37.5 | -1.035028340 | -1.715986641 | -0.3493520360 | World Bank Upper Middle Income | Ischemic stroke | Deaths | Both | Low physical activity | 35 to 39 | 3 |
| 42.5 | -1.510506718 | -2.016489364 | -1.0019111989 | World Bank Upper Middle Income | Ischemic stroke | Deaths | Both | Low physical activity | 40 to 44 | 4 |
| 47.5 | -1.814524640 | -2.188600264 | -1.4390183792 | World Bank Upper Middle Income | Ischemic stroke | Deaths | Both | Low physical activity | 45 to 49 | 5 |
| 52.5 | -2.038803063 | -2.320932724 | -1.7558585180 | World Bank Upper Middle Income | Ischemic stroke | Deaths | Both | Low physical activity | 50 to 54 | 6 |
| 57.5 | -2.164129229 | -2.381138551 | -1.9466374904 | World Bank Upper Middle Income | Ischemic stroke | Deaths | Both | Low physical activity | 55 to 59 | 7 |
| 62.5 | -2.300434329 | -2.458241125 | -2.1423722259 | World Bank Upper Middle Income | Ischemic stroke | Deaths | Both | Low physical activity | 60 to 64 | 8 |
| 67.5 | -2.209767392 | -2.333581880 | -2.0857959411 | World Bank Upper Middle Income | Ischemic stroke | Deaths | Both | Low physical activity | 65 to 69 | 9 |
| 72.5 | -1.893315545 | -1.996388864 | -1.7901338208 | World Bank Upper Middle Income | Ischemic stroke | Deaths | Both | Low physical activity | 70 to 74 | 10 |
| 77.5 | -1.440533588 | -1.526363217 | -1.3546291495 | World Bank Upper Middle Income | Ischemic stroke | Deaths | Both | Low physical activity | 75 to 79 | 11 |
| 82.5 | -1.148871317 | -1.220594474 | -1.0770960830 | World Bank Upper Middle Income | Ischemic stroke | Deaths | Both | Low physical activity | 80 to 84 | 12 |
| 87.5 | -0.832071207 | -0.920605422 | -0.7434578803 | World Bank Upper Middle Income | Ischemic stroke | Deaths | Both | Low physical activity | 85 to 89 | 13 |
| 92.5 | -0.747152203 | -0.911724650 | -0.5823064221 | World Bank Upper Middle Income | Ischemic stroke | Deaths | Both | Low physical activity | 90 to 94 | 14 |
| 27.5 | 1.005251168 | 0.026029610 | 1.9940589799 | World Bank Lower Middle Income | Ischemic stroke | Deaths | Both | Low physical activity | 25 to 29 | 1 |
| 32.5 | 1.052246412 | 0.343748176 | 1.7657471499 | World Bank Lower Middle Income | Ischemic stroke | Deaths | Both | Low physical activity | 30 to 34 | 2 |
| 37.5 | 1.019524783 | 0.457612073 | 1.5845805698 | World Bank Lower Middle Income | Ischemic stroke | Deaths | Both | Low physical activity | 35 to 39 | 3 |
| 42.5 | 0.968752724 | 0.513984299 | 1.4255787170 | World Bank Lower Middle Income | Ischemic stroke | Deaths | Both | Low physical activity | 40 to 44 | 4 |
| 47.5 | 0.825297199 | 0.468461695 | 1.1834000823 | World Bank Lower Middle Income | Ischemic stroke | Deaths | Both | Low physical activity | 45 to 49 | 5 |
| 52.5 | 0.613276706 | 0.340330246 | 0.8869656366 | World Bank Lower Middle Income | Ischemic stroke | Deaths | Both | Low physical activity | 50 to 54 | 6 |
| 57.5 | 0.213300113 | 0.005574687 | 0.4214570142 | World Bank Lower Middle Income | Ischemic stroke | Deaths | Both | Low physical activity | 55 to 59 | 7 |
| 62.5 | -0.153168136 | -0.306629330 | 0.0005292856 | World Bank Lower Middle Income | Ischemic stroke | Deaths | Both | Low physical activity | 60 to 64 | 8 |
| 67.5 | -0.426250226 | -0.550467107 | -0.3018781915 | World Bank Lower Middle Income | Ischemic stroke | Deaths | Both | Low physical activity | 65 to 69 | 9 |
| 72.5 | -0.585026580 | -0.690661652 | -0.4792791435 | World Bank Lower Middle Income | Ischemic stroke | Deaths | Both | Low physical activity | 70 to 74 | 10 |
| 77.5 | -0.704120398 | -0.797958932 | -0.6101930989 | World Bank Lower Middle Income | Ischemic stroke | Deaths | Both | Low physical activity | 75 to 79 | 11 |
| 82.5 | -0.713853206 | -0.799083432 | -0.6285497538 | World Bank Lower Middle Income | Ischemic stroke | Deaths | Both | Low physical activity | 80 to 84 | 12 |
| 87.5 | -0.666624886 | -0.776795959 | -0.5563314852 | World Bank Lower Middle Income | Ischemic stroke | Deaths | Both | Low physical activity | 85 to 89 | 13 |
| 92.5 | -0.568737527 | -0.769671064 | -0.3673971144 | World Bank Lower Middle Income | Ischemic stroke | Deaths | Both | Low physical activity | 90 to 94 | 14 |
| 27.5 | 0.208309326 | -2.282351269 | 2.7624527223 | World Bank Low Income | Ischemic stroke | Deaths | Both | Low physical activity | 25 to 29 | 1 |
| 32.5 | 0.543722544 | -1.310560003 | 2.4328453314 | World Bank Low Income | Ischemic stroke | Deaths | Both | Low physical activity | 30 to 34 | 2 |
| 37.5 | 0.967847831 | -0.526276520 | 2.4844143655 | World Bank Low Income | Ischemic stroke | Deaths | Both | Low physical activity | 35 to 39 | 3 |
| 42.5 | 1.289214227 | 0.089410720 | 2.5034001580 | World Bank Low Income | Ischemic stroke | Deaths | Both | Low physical activity | 40 to 44 | 4 |
| 47.5 | 1.119574712 | 0.156535041 | 2.0918743431 | World Bank Low Income | Ischemic stroke | Deaths | Both | Low physical activity | 45 to 49 | 5 |
| 52.5 | 0.207258797 | -0.578245320 | 0.9989689669 | World Bank Low Income | Ischemic stroke | Deaths | Both | Low physical activity | 50 to 54 | 6 |
| 57.5 | -0.359586312 | -0.993818059 | 0.2787083132 | World Bank Low Income | Ischemic stroke | Deaths | Both | Low physical activity | 55 to 59 | 7 |
| 62.5 | -0.354857261 | -0.840750952 | 0.1334173748 | World Bank Low Income | Ischemic stroke | Deaths | Both | Low physical activity | 60 to 64 | 8 |
| 67.5 | -0.178371544 | -0.578784707 | 0.2236542588 | World Bank Low Income | Ischemic stroke | Deaths | Both | Low physical activity | 65 to 69 | 9 |
| 72.5 | 0.060847314 | -0.281103885 | 0.4039711162 | World Bank Low Income | Ischemic stroke | Deaths | Both | Low physical activity | 70 to 74 | 10 |
| 77.5 | 0.232833304 | -0.081231462 | 0.5478852395 | World Bank Low Income | Ischemic stroke | Deaths | Both | Low physical activity | 75 to 79 | 11 |
| 82.5 | 0.262178158 | -0.042858758 | 0.5681459478 | World Bank Low Income | Ischemic stroke | Deaths | Both | Low physical activity | 80 to 84 | 12 |
| 87.5 | 0.176785827 | -0.241548686 | 0.5968746152 | World Bank Low Income | Ischemic stroke | Deaths | Both | Low physical activity | 85 to 89 | 13 |
| 92.5 | 0.006012478 | -0.734773838 | 0.7523270583 | World Bank Low Income | Ischemic stroke | Deaths | Both | Low physical activity | 90 to 94 | 14 |
| 27.5 | 0.763955816 | -0.189977418 | 1.7270062562 | Global | Ischemic stroke | Deaths | Male | Low physical activity | 25 to 29 | 1 |
| 32.5 | 0.605699104 | -0.044942124 | 1.2605755764 | Global | Ischemic stroke | Deaths | Male | Low physical activity | 30 to 34 | 2 |
| 37.5 | 0.268999933 | -0.216718060 | 0.7570822695 | Global | Ischemic stroke | Deaths | Male | Low physical activity | 35 to 39 | 3 |
| 42.5 | -0.109740465 | -0.478109816 | 0.2599923649 | Global | Ischemic stroke | Deaths | Male | Low physical activity | 40 to 44 | 4 |
| 47.5 | -0.410355800 | -0.684101934 | -0.1358551356 | Global | Ischemic stroke | Deaths | Male | Low physical activity | 45 to 49 | 5 |
| 52.5 | -0.721808934 | -0.928219384 | -0.5149684396 | Global | Ischemic stroke | Deaths | Male | Low physical activity | 50 to 54 | 6 |
| 57.5 | -1.058551817 | -1.216469552 | -0.9003816302 | Global | Ischemic stroke | Deaths | Male | Low physical activity | 55 to 59 | 7 |
| 62.5 | -1.386189979 | -1.503215240 | -1.2690256781 | Global | Ischemic stroke | Deaths | Male | Low physical activity | 60 to 64 | 8 |
| 67.5 | -1.549053075 | -1.643888940 | -1.4541257692 | Global | Ischemic stroke | Deaths | Male | Low physical activity | 65 to 69 | 9 |
| 72.5 | -1.496031624 | -1.577502822 | -1.4144929864 | Global | Ischemic stroke | Deaths | Male | Low physical activity | 70 to 74 | 10 |
| 77.5 | -1.374922086 | -1.446185153 | -1.3036074901 | Global | Ischemic stroke | Deaths | Male | Low physical activity | 75 to 79 | 11 |
| 82.5 | -1.313604191 | -1.376220688 | -1.2509479397 | Global | Ischemic stroke | Deaths | Male | Low physical activity | 80 to 84 | 12 |
| 87.5 | -1.233953559 | -1.313409571 | -1.1544335755 | Global | Ischemic stroke | Deaths | Male | Low physical activity | 85 to 89 | 13 |
| 92.5 | -1.326124416 | -1.472253408 | -1.1797786974 | Global | Ischemic stroke | Deaths | Male | Low physical activity | 90 to 94 | 14 |
| 27.5 | 0.952769333 | -2.457883374 | 4.4826787506 | World Bank High Income | Ischemic stroke | Deaths | Male | Low physical activity | 25 to 29 | 1 |
| 32.5 | 2.022320778 | 0.066374214 | 4.0164992353 | World Bank High Income | Ischemic stroke | Deaths | Male | Low physical activity | 30 to 34 | 2 |
| 37.5 | 1.265740856 | -0.107653791 | 2.6580179600 | World Bank High Income | Ischemic stroke | Deaths | Male | Low physical activity | 35 to 39 | 3 |
| 42.5 | 0.328624725 | -0.700751136 | 1.3686715101 | World Bank High Income | Ischemic stroke | Deaths | Male | Low physical activity | 40 to 44 | 4 |
| 47.5 | -0.839510885 | -1.622078963 | -0.0507177026 | World Bank High Income | Ischemic stroke | Deaths | Male | Low physical activity | 45 to 49 | 5 |
| 52.5 | -1.801644684 | -2.407175938 | -1.1923563086 | World Bank High Income | Ischemic stroke | Deaths | Male | Low physical activity | 50 to 54 | 6 |
| 57.5 | -2.764457989 | -3.232565146 | -2.2940863897 | World Bank High Income | Ischemic stroke | Deaths | Male | Low physical activity | 55 to 59 | 7 |
| 62.5 | -3.605027012 | -3.953723872 | -3.2550642057 | World Bank High Income | Ischemic stroke | Deaths | Male | Low physical activity | 60 to 64 | 8 |
| 67.5 | -4.323127293 | -4.594807642 | -4.0506732952 | World Bank High Income | Ischemic stroke | Deaths | Male | Low physical activity | 65 to 69 | 9 |
| 72.5 | -4.577907931 | -4.794092433 | -4.3612325384 | World Bank High Income | Ischemic stroke | Deaths | Male | Low physical activity | 70 to 74 | 10 |
| 77.5 | -4.525900017 | -4.696872696 | -4.3546206155 | World Bank High Income | Ischemic stroke | Deaths | Male | Low physical activity | 75 to 79 | 11 |
| 82.5 | -4.154150264 | -4.280979459 | -4.0271530180 | World Bank High Income | Ischemic stroke | Deaths | Male | Low physical activity | 80 to 84 | 12 |
| 87.5 | -3.566604969 | -3.702235777 | -3.4307831317 | World Bank High Income | Ischemic stroke | Deaths | Male | Low physical activity | 85 to 89 | 13 |
| 92.5 | -2.938432940 | -3.162255111 | -2.7140934465 | World Bank High Income | Ischemic stroke | Deaths | Male | Low physical activity | 90 to 94 | 14 |
| 27.5 | -0.065197567 | -1.666795550 | 1.5624863769 | World Bank Upper Middle Income | Ischemic stroke | Deaths | Male | Low physical activity | 25 to 29 | 1 |
| 32.5 | -0.251225663 | -1.263902826 | 0.7718379239 | World Bank Upper Middle Income | Ischemic stroke | Deaths | Male | Low physical activity | 30 to 34 | 2 |
| 37.5 | -0.569358850 | -1.293942819 | 0.1605441624 | World Bank Upper Middle Income | Ischemic stroke | Deaths | Male | Low physical activity | 35 to 39 | 3 |
| 42.5 | -1.068712491 | -1.594176599 | -0.5404425275 | World Bank Upper Middle Income | Ischemic stroke | Deaths | Male | Low physical activity | 40 to 44 | 4 |
| 47.5 | -1.385978439 | -1.766041519 | -1.0044449105 | World Bank Upper Middle Income | Ischemic stroke | Deaths | Male | Low physical activity | 45 to 49 | 5 |
| 52.5 | -1.603216062 | -1.888069843 | -1.3175352498 | World Bank Upper Middle Income | Ischemic stroke | Deaths | Male | Low physical activity | 50 to 54 | 6 |
| 57.5 | -1.726849476 | -1.946374225 | -1.5068332496 | World Bank Upper Middle Income | Ischemic stroke | Deaths | Male | Low physical activity | 55 to 59 | 7 |
| 62.5 | -1.910226917 | -2.072619014 | -1.7475655278 | World Bank Upper Middle Income | Ischemic stroke | Deaths | Male | Low physical activity | 60 to 64 | 8 |
| 67.5 | -1.850489868 | -1.982245624 | -1.7185570056 | World Bank Upper Middle Income | Ischemic stroke | Deaths | Male | Low physical activity | 65 to 69 | 9 |
| 72.5 | -1.538843268 | -1.653305062 | -1.4242482558 | World Bank Upper Middle Income | Ischemic stroke | Deaths | Male | Low physical activity | 70 to 74 | 10 |
| 77.5 | -1.081343943 | -1.182373018 | -0.9802115791 | World Bank Upper Middle Income | Ischemic stroke | Deaths | Male | Low physical activity | 75 to 79 | 11 |
| 82.5 | -0.745531350 | -0.837806730 | -0.6531701020 | World Bank Upper Middle Income | Ischemic stroke | Deaths | Male | Low physical activity | 80 to 84 | 12 |
| 87.5 | -0.282399144 | -0.408169136 | -0.1564703229 | World Bank Upper Middle Income | Ischemic stroke | Deaths | Male | Low physical activity | 85 to 89 | 13 |
| 92.5 | -0.248867440 | -0.502367150 | 0.0052781355 | World Bank Upper Middle Income | Ischemic stroke | Deaths | Male | Low physical activity | 90 to 94 | 14 |
| 27.5 | 1.275662589 | -0.098475602 | 2.6687019513 | World Bank Lower Middle Income | Ischemic stroke | Deaths | Male | Low physical activity | 25 to 29 | 1 |
| 32.5 | 1.146203829 | 0.138521998 | 2.1640258393 | World Bank Lower Middle Income | Ischemic stroke | Deaths | Male | Low physical activity | 30 to 34 | 2 |
| 37.5 | 0.982555535 | 0.182658123 | 1.7888396397 | World Bank Lower Middle Income | Ischemic stroke | Deaths | Male | Low physical activity | 35 to 39 | 3 |
| 42.5 | 0.916037100 | 0.277401717 | 1.5587397513 | World Bank Lower Middle Income | Ischemic stroke | Deaths | Male | Low physical activity | 40 to 44 | 4 |
| 47.5 | 0.795950115 | 0.310019737 | 1.2842344792 | World Bank Lower Middle Income | Ischemic stroke | Deaths | Male | Low physical activity | 45 to 49 | 5 |
| 52.5 | 0.571102158 | 0.206532936 | 0.9369977482 | World Bank Lower Middle Income | Ischemic stroke | Deaths | Male | Low physical activity | 50 to 54 | 6 |
| 57.5 | 0.203498084 | -0.071687854 | 0.4794418384 | World Bank Lower Middle Income | Ischemic stroke | Deaths | Male | Low physical activity | 55 to 59 | 7 |
| 62.5 | -0.193068074 | -0.397771434 | 0.0120559952 | World Bank Lower Middle Income | Ischemic stroke | Deaths | Male | Low physical activity | 60 to 64 | 8 |
| 67.5 | -0.459858845 | -0.629212062 | -0.2902170071 | World Bank Lower Middle Income | Ischemic stroke | Deaths | Male | Low physical activity | 65 to 69 | 9 |
| 72.5 | -0.567313961 | -0.716905296 | -0.4174972349 | World Bank Lower Middle Income | Ischemic stroke | Deaths | Male | Low physical activity | 70 to 74 | 10 |
| 77.5 | -0.601445653 | -0.739949159 | -0.4627488839 | World Bank Lower Middle Income | Ischemic stroke | Deaths | Male | Low physical activity | 75 to 79 | 11 |
| 82.5 | -0.588285039 | -0.719210792 | -0.4571866276 | World Bank Lower Middle Income | Ischemic stroke | Deaths | Male | Low physical activity | 80 to 84 | 12 |
| 87.5 | -0.558390965 | -0.732149234 | -0.3843285488 | World Bank Lower Middle Income | Ischemic stroke | Deaths | Male | Low physical activity | 85 to 89 | 13 |
| 92.5 | -0.558144685 | -0.881634283 | -0.2335993246 | World Bank Lower Middle Income | Ischemic stroke | Deaths | Male | Low physical activity | 90 to 94 | 14 |
| 27.5 | 0.929097503 | -2.817050001 | 4.8196491547 | World Bank Low Income | Ischemic stroke | Deaths | Male | Low physical activity | 25 to 29 | 1 |
| 32.5 | 1.049882193 | -1.911989774 | 4.1011910394 | World Bank Low Income | Ischemic stroke | Deaths | Male | Low physical activity | 30 to 34 | 2 |
| 37.5 | 1.213099273 | -1.216217194 | 3.7021581230 | World Bank Low Income | Ischemic stroke | Deaths | Male | Low physical activity | 35 to 39 | 3 |
| 42.5 | 1.531881206 | -0.348792824 | 3.4480483810 | World Bank Low Income | Ischemic stroke | Deaths | Male | Low physical activity | 40 to 44 | 4 |
| 47.5 | 1.036591635 | -0.424868829 | 2.5195018984 | World Bank Low Income | Ischemic stroke | Deaths | Male | Low physical activity | 45 to 49 | 5 |
| 52.5 | 0.189767331 | -0.969150636 | 1.3622476468 | World Bank Low Income | Ischemic stroke | Deaths | Male | Low physical activity | 50 to 54 | 6 |
| 57.5 | -0.296237100 | -1.212694856 | 0.6287227077 | World Bank Low Income | Ischemic stroke | Deaths | Male | Low physical activity | 55 to 59 | 7 |
| 62.5 | -0.294050721 | -0.991573038 | 0.4083856973 | World Bank Low Income | Ischemic stroke | Deaths | Male | Low physical activity | 60 to 64 | 8 |
| 67.5 | -0.104465468 | -0.690100824 | 0.4846234094 | World Bank Low Income | Ischemic stroke | Deaths | Male | Low physical activity | 65 to 69 | 9 |
| 72.5 | 0.154199756 | -0.363444510 | 0.6745333519 | World Bank Low Income | Ischemic stroke | Deaths | Male | Low physical activity | 70 to 74 | 10 |
| 77.5 | 0.259898978 | -0.232390794 | 0.7546178871 | World Bank Low Income | Ischemic stroke | Deaths | Male | Low physical activity | 75 to 79 | 11 |
| 82.5 | 0.162963165 | -0.331334524 | 0.6597122773 | World Bank Low Income | Ischemic stroke | Deaths | Male | Low physical activity | 80 to 84 | 12 |
| 87.5 | 0.029711749 | -0.668601681 | 0.7329344189 | World Bank Low Income | Ischemic stroke | Deaths | Male | Low physical activity | 85 to 89 | 13 |
| 92.5 | 0.042098967 | -1.291619007 | 1.3938377373 | World Bank Low Income | Ischemic stroke | Deaths | Male | Low physical activity | 90 to 94 | 14 |
| 27.5 | -0.285046014 | -1.210574797 | 0.6491537734 | Global | Ischemic stroke | Deaths | Female | Low physical activity | 25 to 29 | 1 |
| 32.5 | -0.024821623 | -0.656090151 | 0.6104582213 | Global | Ischemic stroke | Deaths | Female | Low physical activity | 30 to 34 | 2 |
| 37.5 | -0.107410834 | -0.591200019 | 0.3787327909 | Global | Ischemic stroke | Deaths | Female | Low physical activity | 35 to 39 | 3 |
| 42.5 | -0.427528905 | -0.812338247 | -0.0412266522 | Global | Ischemic stroke | Deaths | Female | Low physical activity | 40 to 44 | 4 |
| 47.5 | -0.778758342 | -1.083463879 | -0.4731141803 | Global | Ischemic stroke | Deaths | Female | Low physical activity | 45 to 49 | 5 |
| 52.5 | -1.155369687 | -1.392697623 | -0.9174705498 | Global | Ischemic stroke | Deaths | Female | Low physical activity | 50 to 54 | 6 |
| 57.5 | -1.548649240 | -1.731875611 | -1.3650812321 | Global | Ischemic stroke | Deaths | Female | Low physical activity | 55 to 59 | 7 |
| 62.5 | -1.814809449 | -1.947036389 | -1.6824041975 | Global | Ischemic stroke | Deaths | Female | Low physical activity | 60 to 64 | 8 |
| 67.5 | -1.969699293 | -2.070384603 | -1.8689104650 | Global | Ischemic stroke | Deaths | Female | Low physical activity | 65 to 69 | 9 |
| 72.5 | -1.928803141 | -2.007909299 | -1.8496331232 | Global | Ischemic stroke | Deaths | Female | Low physical activity | 70 to 74 | 10 |
| 77.5 | -1.845542282 | -1.907952937 | -1.7830919176 | Global | Ischemic stroke | Deaths | Female | Low physical activity | 75 to 79 | 11 |
| 82.5 | -1.818866857 | -1.866129428 | -1.7715815229 | Global | Ischemic stroke | Deaths | Female | Low physical activity | 80 to 84 | 12 |
| 87.5 | -1.799688080 | -1.850258522 | -1.7490915815 | Global | Ischemic stroke | Deaths | Female | Low physical activity | 85 to 89 | 13 |
| 92.5 | -1.763553016 | -1.845832686 | -1.6812043723 | Global | Ischemic stroke | Deaths | Female | Low physical activity | 90 to 94 | 14 |
| 27.5 | 0.671600141 | -2.179498582 | 3.6057976402 | World Bank High Income | Ischemic stroke | Deaths | Female | Low physical activity | 25 to 29 | 1 |
| 32.5 | 1.126403322 | -0.709248075 | 2.9959915774 | World Bank High Income | Ischemic stroke | Deaths | Female | Low physical activity | 30 to 34 | 2 |
| 37.5 | 0.976761548 | -0.381219088 | 2.3532538686 | World Bank High Income | Ischemic stroke | Deaths | Female | Low physical activity | 35 to 39 | 3 |
| 42.5 | 0.267493637 | -0.839884963 | 1.3872389766 | World Bank High Income | Ischemic stroke | Deaths | Female | Low physical activity | 40 to 44 | 4 |
| 47.5 | -0.783534886 | -1.702614685 | 0.1441383022 | World Bank High Income | Ischemic stroke | Deaths | Female | Low physical activity | 45 to 49 | 5 |
| 52.5 | -1.777525015 | -2.524091418 | -1.0252406720 | World Bank High Income | Ischemic stroke | Deaths | Female | Low physical activity | 50 to 54 | 6 |
| 57.5 | -2.949100813 | -3.531917110 | -2.3627634040 | World Bank High Income | Ischemic stroke | Deaths | Female | Low physical activity | 55 to 59 | 7 |
| 62.5 | -4.049120248 | -4.474111277 | -3.6222384502 | World Bank High Income | Ischemic stroke | Deaths | Female | Low physical activity | 60 to 64 | 8 |
| 67.5 | -4.890094870 | -5.200955895 | -4.5782144826 | World Bank High Income | Ischemic stroke | Deaths | Female | Low physical activity | 65 to 69 | 9 |
| 72.5 | -5.087828361 | -5.307390047 | -4.8677575825 | World Bank High Income | Ischemic stroke | Deaths | Female | Low physical activity | 70 to 74 | 10 |
| 77.5 | -4.881697917 | -5.034809028 | -4.7283399479 | World Bank High Income | Ischemic stroke | Deaths | Female | Low physical activity | 75 to 79 | 11 |
| 82.5 | -4.229574985 | -4.321694967 | -4.1373663083 | World Bank High Income | Ischemic stroke | Deaths | Female | Low physical activity | 80 to 84 | 12 |
| 87.5 | -3.420722023 | -3.498882392 | -3.3424983494 | World Bank High Income | Ischemic stroke | Deaths | Female | Low physical activity | 85 to 89 | 13 |
| 92.5 | -2.584497096 | -2.697824070 | -2.4710381312 | World Bank High Income | Ischemic stroke | Deaths | Female | Low physical activity | 90 to 94 | 14 |
| 27.5 | -1.607467819 | -3.718635174 | 0.5499912322 | World Bank Upper Middle Income | Ischemic stroke | Deaths | Female | Low physical activity | 25 to 29 | 1 |
| 32.5 | -1.502845432 | -2.896207270 | -0.0894899649 | World Bank Upper Middle Income | Ischemic stroke | Deaths | Female | Low physical activity | 30 to 34 | 2 |
| 37.5 | -1.684329814 | -2.727415998 | -0.6300582706 | World Bank Upper Middle Income | Ischemic stroke | Deaths | Female | Low physical activity | 35 to 39 | 3 |
| 42.5 | -2.166845065 | -2.962654551 | -1.3645090945 | World Bank Upper Middle Income | Ischemic stroke | Deaths | Female | Low physical activity | 40 to 44 | 4 |
| 47.5 | -2.485289638 | -3.090444071 | -1.8763563010 | World Bank Upper Middle Income | Ischemic stroke | Deaths | Female | Low physical activity | 45 to 49 | 5 |
| 52.5 | -2.715957944 | -3.176794461 | -2.2529280440 | World Bank Upper Middle Income | Ischemic stroke | Deaths | Female | Low physical activity | 50 to 54 | 6 |
| 57.5 | -2.818229169 | -3.172502993 | -2.4626591224 | World Bank Upper Middle Income | Ischemic stroke | Deaths | Female | Low physical activity | 55 to 59 | 7 |
| 62.5 | -2.839497938 | -3.092124246 | -2.5862130661 | World Bank Upper Middle Income | Ischemic stroke | Deaths | Female | Low physical activity | 60 to 64 | 8 |
| 67.5 | -2.642151511 | -2.833601329 | -2.4503244748 | World Bank Upper Middle Income | Ischemic stroke | Deaths | Female | Low physical activity | 65 to 69 | 9 |
| 72.5 | -2.241552899 | -2.394578314 | -2.0882875719 | World Bank Upper Middle Income | Ischemic stroke | Deaths | Female | Low physical activity | 70 to 74 | 10 |
| 77.5 | -1.696640901 | -1.818298239 | -1.5748328166 | World Bank Upper Middle Income | Ischemic stroke | Deaths | Female | Low physical activity | 75 to 79 | 11 |
| 82.5 | -1.351691147 | -1.447397910 | -1.2558914416 | World Bank Upper Middle Income | Ischemic stroke | Deaths | Female | Low physical activity | 80 to 84 | 12 |
| 87.5 | -1.071915671 | -1.183767664 | -0.9599370719 | World Bank Upper Middle Income | Ischemic stroke | Deaths | Female | Low physical activity | 85 to 89 | 13 |
| 92.5 | -0.963686604 | -1.165150516 | -0.7618120310 | World Bank Upper Middle Income | Ischemic stroke | Deaths | Female | Low physical activity | 90 to 94 | 14 |
| 27.5 | 0.665455818 | -0.724568577 | 2.0749429123 | World Bank Lower Middle Income | Ischemic stroke | Deaths | Female | Low physical activity | 25 to 29 | 1 |
| 32.5 | 0.925153006 | -0.069378335 | 1.9295821398 | World Bank Lower Middle Income | Ischemic stroke | Deaths | Female | Low physical activity | 30 to 34 | 2 |
| 37.5 | 1.010869503 | 0.220148748 | 1.8078289172 | World Bank Lower Middle Income | Ischemic stroke | Deaths | Female | Low physical activity | 35 to 39 | 3 |
| 42.5 | 1.029327722 | 0.380462226 | 1.6823875258 | World Bank Lower Middle Income | Ischemic stroke | Deaths | Female | Low physical activity | 40 to 44 | 4 |
| 47.5 | 0.877495325 | 0.350969120 | 1.4067841323 | World Bank Lower Middle Income | Ischemic stroke | Deaths | Female | Low physical activity | 45 to 49 | 5 |
| 52.5 | 0.689644866 | 0.277405121 | 1.1035793262 | World Bank Lower Middle Income | Ischemic stroke | Deaths | Female | Low physical activity | 50 to 54 | 6 |
| 57.5 | 0.244934738 | -0.072236556 | 0.5631127352 | World Bank Lower Middle Income | Ischemic stroke | Deaths | Female | Low physical activity | 55 to 59 | 7 |
| 62.5 | -0.090401549 | -0.322813146 | 0.1425519481 | World Bank Lower Middle Income | Ischemic stroke | Deaths | Female | Low physical activity | 60 to 64 | 8 |
| 67.5 | -0.374525717 | -0.558068542 | -0.1906441219 | World Bank Lower Middle Income | Ischemic stroke | Deaths | Female | Low physical activity | 65 to 69 | 9 |
| 72.5 | -0.585651048 | -0.735618165 | -0.4354573633 | World Bank Lower Middle Income | Ischemic stroke | Deaths | Female | Low physical activity | 70 to 74 | 10 |
| 77.5 | -0.776865776 | -0.905126321 | -0.6484392205 | World Bank Lower Middle Income | Ischemic stroke | Deaths | Female | Low physical activity | 75 to 79 | 11 |
| 82.5 | -0.803683272 | -0.916202010 | -0.6910367597 | World Bank Lower Middle Income | Ischemic stroke | Deaths | Female | Low physical activity | 80 to 84 | 12 |
| 87.5 | -0.745355215 | -0.887878941 | -0.6026265397 | World Bank Lower Middle Income | Ischemic stroke | Deaths | Female | Low physical activity | 85 to 89 | 13 |
| 92.5 | -0.573497752 | -0.830252469 | -0.3160782860 | World Bank Lower Middle Income | Ischemic stroke | Deaths | Female | Low physical activity | 90 to 94 | 14 |
| 27.5 | -0.550445939 | -3.855088415 | 2.8677819759 | World Bank Low Income | Ischemic stroke | Deaths | Female | Low physical activity | 25 to 29 | 1 |
| 32.5 | 0.159061915 | -2.204375051 | 2.5796163068 | World Bank Low Income | Ischemic stroke | Deaths | Female | Low physical activity | 30 to 34 | 2 |
| 37.5 | 0.853897430 | -1.029847041 | 2.7734960774 | World Bank Low Income | Ischemic stroke | Deaths | Female | Low physical activity | 35 to 39 | 3 |
| 42.5 | 1.239340903 | -0.303817078 | 2.8063848191 | World Bank Low Income | Ischemic stroke | Deaths | Female | Low physical activity | 40 to 44 | 4 |
| 47.5 | 1.048823561 | -0.226800882 | 2.3407571699 | World Bank Low Income | Ischemic stroke | Deaths | Female | Low physical activity | 45 to 49 | 5 |
| 52.5 | 0.178082979 | -0.889816619 | 1.2574890581 | World Bank Low Income | Ischemic stroke | Deaths | Female | Low physical activity | 50 to 54 | 6 |
| 57.5 | -0.408651203 | -1.287860298 | 0.4783888293 | World Bank Low Income | Ischemic stroke | Deaths | Female | Low physical activity | 55 to 59 | 7 |
| 62.5 | -0.404029747 | -1.081729269 | 0.2783127661 | World Bank Low Income | Ischemic stroke | Deaths | Female | Low physical activity | 60 to 64 | 8 |
| 67.5 | -0.254080503 | -0.803825469 | 0.2987111475 | World Bank Low Income | Ischemic stroke | Deaths | Female | Low physical activity | 65 to 69 | 9 |
| 72.5 | -0.059972172 | -0.516296426 | 0.3984452064 | World Bank Low Income | Ischemic stroke | Deaths | Female | Low physical activity | 70 to 74 | 10 |
| 77.5 | 0.138808615 | -0.269943190 | 0.5492357226 | World Bank Low Income | Ischemic stroke | Deaths | Female | Low physical activity | 75 to 79 | 11 |
| 82.5 | 0.259928532 | -0.127841568 | 0.6492042147 | World Bank Low Income | Ischemic stroke | Deaths | Female | Low physical activity | 80 to 84 | 12 |
| 87.5 | 0.222790493 | -0.299187331 | 0.7475011015 | World Bank Low Income | Ischemic stroke | Deaths | Female | Low physical activity | 85 to 89 | 13 |
| 92.5 | 0.054519458 | -0.843075812 | 0.9602400033 | World Bank Low Income | Ischemic stroke | Deaths | Female | Low physical activity | 90 to 94 | 14 |

**Supplementary Table 6. Longitudinal Age curve results of Age-Period-Cohort analysis**

| Age | Rate | CI Lo | CI Hi | location | cause | measure | sex | rei | label | x |
| --- | --- | --- | --- | --- | --- | --- | --- | --- | --- | --- |
| 27.5 | 0.021016059 | 0.018953954 | 0.02330251 | Global | Ischemic stroke | Deaths | Both | Low physical activity | 25 to 29 | 1 |
| 32.5 | 0.032569924 | 0.030004089 | 0.03535518 | Global | Ischemic stroke | Deaths | Both | Low physical activity | 30 to 34 | 2 |
| 37.5 | 0.056769250 | 0.053227572 | 0.06054659 | Global | Ischemic stroke | Deaths | Both | Low physical activity | 35 to 39 | 3 |
| 42.5 | 0.096759979 | 0.091971702 | 0.10179754 | Global | Ischemic stroke | Deaths | Both | Low physical activity | 40 to 44 | 4 |
| 47.5 | 0.191973146 | 0.184705943 | 0.19952628 | Global | Ischemic stroke | Deaths | Both | Low physical activity | 45 to 49 | 5 |
| 52.5 | 0.368683628 | 0.357733820 | 0.37996860 | Global | Ischemic stroke | Deaths | Both | Low physical activity | 50 to 54 | 6 |
| 57.5 | 0.647030464 | 0.631169520 | 0.66328998 | Global | Ischemic stroke | Deaths | Both | Low physical activity | 55 to 59 | 7 |
| 62.5 | 1.669841321 | 1.637738429 | 1.70257349 | Global | Ischemic stroke | Deaths | Both | Low physical activity | 60 to 64 | 8 |
| 67.5 | 2.980076450 | 2.926657574 | 3.03447036 | Global | Ischemic stroke | Deaths | Both | Low physical activity | 65 to 69 | 9 |
| 72.5 | 6.133316842 | 6.011081011 | 6.25803835 | Global | Ischemic stroke | Deaths | Both | Low physical activity | 70 to 74 | 10 |
| 77.5 | 9.836912290 | 9.639111655 | 10.03877192 | Global | Ischemic stroke | Deaths | Both | Low physical activity | 75 to 79 | 11 |
| 82.5 | 38.547808246 | 37.787369688 | 39.32354998 | Global | Ischemic stroke | Deaths | Both | Low physical activity | 80 to 84 | 12 |
| 87.5 | 59.869356272 | 58.664942091 | 61.09849755 | Global | Ischemic stroke | Deaths | Both | Low physical activity | 85 to 89 | 13 |
| 92.5 | 81.897091237 | 80.180348749 | 83.65059092 | Global | Ischemic stroke | Deaths | Both | Low physical activity | 90 to 94 | 14 |
| 27.5 | 0.011589150 | 0.007799560 | 0.01721999 | World Bank High Income | Ischemic stroke | Deaths | Both | Low physical activity | 25 to 29 | 1 |
| 32.5 | 0.020179992 | 0.014984618 | 0.02717668 | World Bank High Income | Ischemic stroke | Deaths | Both | Low physical activity | 30 to 34 | 2 |
| 37.5 | 0.038753132 | 0.030928074 | 0.04855800 | World Bank High Income | Ischemic stroke | Deaths | Both | Low physical activity | 35 to 39 | 3 |
| 42.5 | 0.061875196 | 0.051884485 | 0.07378969 | World Bank High Income | Ischemic stroke | Deaths | Both | Low physical activity | 40 to 44 | 4 |
| 47.5 | 0.110260496 | 0.096247669 | 0.12631347 | World Bank High Income | Ischemic stroke | Deaths | Both | Low physical activity | 45 to 49 | 5 |
| 52.5 | 0.174659485 | 0.156385364 | 0.19506899 | World Bank High Income | Ischemic stroke | Deaths | Both | Low physical activity | 50 to 54 | 6 |
| 57.5 | 0.257799969 | 0.234770398 | 0.28308860 | World Bank High Income | Ischemic stroke | Deaths | Both | Low physical activity | 55 to 59 | 7 |
| 62.5 | 0.544068198 | 0.503485788 | 0.58792167 | World Bank High Income | Ischemic stroke | Deaths | Both | Low physical activity | 60 to 64 | 8 |
| 67.5 | 0.836964522 | 0.777634707 | 0.90082092 | World Bank High Income | Ischemic stroke | Deaths | Both | Low physical activity | 65 to 69 | 9 |
| 72.5 | 1.710630742 | 1.582504923 | 1.84913013 | World Bank High Income | Ischemic stroke | Deaths | Both | Low physical activity | 70 to 74 | 10 |
| 77.5 | 2.732595219 | 2.528917866 | 2.95267661 | World Bank High Income | Ischemic stroke | Deaths | Both | Low physical activity | 75 to 79 | 11 |
| 82.5 | 11.681095750 | 10.824501337 | 12.60547656 | World Bank High Income | Ischemic stroke | Deaths | Both | Low physical activity | 80 to 84 | 12 |
| 87.5 | 19.316291845 | 17.895694070 | 20.84965965 | World Bank High Income | Ischemic stroke | Deaths | Both | Low physical activity | 85 to 89 | 13 |
| 92.5 | 28.603335240 | 26.483997534 | 30.89226941 | World Bank High Income | Ischemic stroke | Deaths | Both | Low physical activity | 90 to 94 | 14 |
| 27.5 | 0.029326864 | 0.023904020 | 0.03597993 | World Bank Upper Middle Income | Ischemic stroke | Deaths | Both | Low physical activity | 25 to 29 | 1 |
| 32.5 | 0.046374568 | 0.039620501 | 0.05427999 | World Bank Upper Middle Income | Ischemic stroke | Deaths | Both | Low physical activity | 30 to 34 | 2 |
| 37.5 | 0.079761674 | 0.070751817 | 0.08991889 | World Bank Upper Middle Income | Ischemic stroke | Deaths | Both | Low physical activity | 35 to 39 | 3 |
| 42.5 | 0.135749438 | 0.123815304 | 0.14883386 | World Bank Upper Middle Income | Ischemic stroke | Deaths | Both | Low physical activity | 40 to 44 | 4 |
| 47.5 | 0.265642161 | 0.247994401 | 0.28454577 | World Bank Upper Middle Income | Ischemic stroke | Deaths | Both | Low physical activity | 45 to 49 | 5 |
| 52.5 | 0.476345132 | 0.451323323 | 0.50275418 | World Bank Upper Middle Income | Ischemic stroke | Deaths | Both | Low physical activity | 50 to 54 | 6 |
| 57.5 | 0.826527106 | 0.790677414 | 0.86400224 | World Bank Upper Middle Income | Ischemic stroke | Deaths | Both | Low physical activity | 55 to 59 | 7 |
| 62.5 | 1.963600820 | 1.895265615 | 2.03439990 | World Bank Upper Middle Income | Ischemic stroke | Deaths | Both | Low physical activity | 60 to 64 | 8 |
| 67.5 | 3.568802085 | 3.453500545 | 3.68795318 | World Bank Upper Middle Income | Ischemic stroke | Deaths | Both | Low physical activity | 65 to 69 | 9 |
| 72.5 | 7.297981360 | 7.036831430 | 7.56882305 | World Bank Upper Middle Income | Ischemic stroke | Deaths | Both | Low physical activity | 70 to 74 | 10 |
| 77.5 | 12.184407972 | 11.745804338 | 12.63938964 | World Bank Upper Middle Income | Ischemic stroke | Deaths | Both | Low physical activity | 75 to 79 | 11 |
| 82.5 | 49.336490686 | 47.592731838 | 51.14413943 | World Bank Upper Middle Income | Ischemic stroke | Deaths | Both | Low physical activity | 80 to 84 | 12 |
| 87.5 | 78.897962709 | 76.040481929 | 81.86282309 | World Bank Upper Middle Income | Ischemic stroke | Deaths | Both | Low physical activity | 85 to 89 | 13 |
| 92.5 | 103.935609531 | 99.932601991 | 108.09896584 | World Bank Upper Middle Income | Ischemic stroke | Deaths | Both | Low physical activity | 90 to 94 | 14 |
| 27.5 | 0.017600250 | 0.014936609 | 0.02073890 | World Bank Lower Middle Income | Ischemic stroke | Deaths | Both | Low physical activity | 25 to 29 | 1 |
| 32.5 | 0.026042590 | 0.022727177 | 0.02984165 | World Bank Lower Middle Income | Ischemic stroke | Deaths | Both | Low physical activity | 30 to 34 | 2 |
| 37.5 | 0.044895497 | 0.040169830 | 0.05017710 | World Bank Lower Middle Income | Ischemic stroke | Deaths | Both | Low physical activity | 35 to 39 | 3 |
| 42.5 | 0.077142167 | 0.070441793 | 0.08447988 | World Bank Lower Middle Income | Ischemic stroke | Deaths | Both | Low physical activity | 40 to 44 | 4 |
| 47.5 | 0.164621631 | 0.153500472 | 0.17654852 | World Bank Lower Middle Income | Ischemic stroke | Deaths | Both | Low physical activity | 45 to 49 | 5 |
| 52.5 | 0.375390442 | 0.355896639 | 0.39595199 | World Bank Lower Middle Income | Ischemic stroke | Deaths | Both | Low physical activity | 50 to 54 | 6 |
| 57.5 | 0.717033306 | 0.686545594 | 0.74887490 | World Bank Lower Middle Income | Ischemic stroke | Deaths | Both | Low physical activity | 55 to 59 | 7 |
| 62.5 | 2.237831129 | 2.166620355 | 2.31138240 | World Bank Lower Middle Income | Ischemic stroke | Deaths | Both | Low physical activity | 60 to 64 | 8 |
| 67.5 | 4.206197001 | 4.080975340 | 4.33526099 | World Bank Lower Middle Income | Ischemic stroke | Deaths | Both | Low physical activity | 65 to 69 | 9 |
| 72.5 | 9.172382028 | 8.861710087 | 9.49394544 | World Bank Lower Middle Income | Ischemic stroke | Deaths | Both | Low physical activity | 70 to 74 | 10 |
| 77.5 | 14.812517555 | 14.297700857 | 15.34587124 | World Bank Lower Middle Income | Ischemic stroke | Deaths | Both | Low physical activity | 75 to 79 | 11 |
| 82.5 | 55.928559387 | 54.009041249 | 57.91629851 | World Bank Lower Middle Income | Ischemic stroke | Deaths | Both | Low physical activity | 80 to 84 | 12 |
| 87.5 | 85.608733091 | 82.541807982 | 88.78961293 | World Bank Lower Middle Income | Ischemic stroke | Deaths | Both | Low physical activity | 85 to 89 | 13 |
| 92.5 | 120.662632759 | 115.894354408 | 125.62709391 | World Bank Lower Middle Income | Ischemic stroke | Deaths | Both | Low physical activity | 90 to 94 | 14 |
| 27.5 | 0.014551952 | 0.009332497 | 0.02269053 | World Bank Low Income | Ischemic stroke | Deaths | Both | Low physical activity | 25 to 29 | 1 |
| 32.5 | 0.025022990 | 0.017423449 | 0.03593720 | World Bank Low Income | Ischemic stroke | Deaths | Both | Low physical activity | 30 to 34 | 2 |
| 37.5 | 0.042495913 | 0.031436152 | 0.05744668 | World Bank Low Income | Ischemic stroke | Deaths | Both | Low physical activity | 35 to 39 | 3 |
| 42.5 | 0.083467521 | 0.065538476 | 0.10630133 | World Bank Low Income | Ischemic stroke | Deaths | Both | Low physical activity | 40 to 44 | 4 |
| 47.5 | 0.149838425 | 0.122547503 | 0.18320694 | World Bank Low Income | Ischemic stroke | Deaths | Both | Low physical activity | 45 to 49 | 5 |
| 52.5 | 0.318592738 | 0.272002278 | 0.37316354 | World Bank Low Income | Ischemic stroke | Deaths | Both | Low physical activity | 50 to 54 | 6 |
| 57.5 | 0.571844770 | 0.500314966 | 0.65360116 | World Bank Low Income | Ischemic stroke | Deaths | Both | Low physical activity | 55 to 59 | 7 |
| 62.5 | 1.678392405 | 1.512988760 | 1.86187838 | World Bank Low Income | Ischemic stroke | Deaths | Both | Low physical activity | 60 to 64 | 8 |
| 67.5 | 3.039489238 | 2.756393095 | 3.35166085 | World Bank Low Income | Ischemic stroke | Deaths | Both | Low physical activity | 65 to 69 | 9 |
| 72.5 | 6.466971828 | 5.789591802 | 7.22360506 | World Bank Low Income | Ischemic stroke | Deaths | Both | Low physical activity | 70 to 74 | 10 |
| 77.5 | 10.500976782 | 9.370137075 | 11.76829245 | World Bank Low Income | Ischemic stroke | Deaths | Both | Low physical activity | 75 to 79 | 11 |
| 82.5 | 39.298637802 | 35.117682776 | 43.97735873 | World Bank Low Income | Ischemic stroke | Deaths | Both | Low physical activity | 80 to 84 | 12 |
| 87.5 | 54.937196862 | 48.765407452 | 61.89009293 | World Bank Low Income | Ischemic stroke | Deaths | Both | Low physical activity | 85 to 89 | 13 |
| 92.5 | 77.622572324 | 67.855886755 | 88.79500398 | World Bank Low Income | Ischemic stroke | Deaths | Both | Low physical activity | 90 to 94 | 14 |
| 27.5 | 0.018144408 | 0.015597253 | 0.02110753 | Global | Ischemic stroke | Deaths | Male | Low physical activity | 25 to 29 | 1 |
| 32.5 | 0.029058998 | 0.025816498 | 0.03270875 | Global | Ischemic stroke | Deaths | Male | Low physical activity | 30 to 34 | 2 |
| 37.5 | 0.053415665 | 0.048782590 | 0.05848876 | Global | Ischemic stroke | Deaths | Male | Low physical activity | 35 to 39 | 3 |
| 42.5 | 0.096891267 | 0.090365846 | 0.10388790 | Global | Ischemic stroke | Deaths | Male | Low physical activity | 40 to 44 | 4 |
| 47.5 | 0.215880880 | 0.205120428 | 0.22720582 | Global | Ischemic stroke | Deaths | Male | Low physical activity | 45 to 49 | 5 |
| 52.5 | 0.426815827 | 0.410236244 | 0.44406547 | Global | Ischemic stroke | Deaths | Male | Low physical activity | 50 to 54 | 6 |
| 57.5 | 0.759771391 | 0.735332546 | 0.78502246 | Global | Ischemic stroke | Deaths | Male | Low physical activity | 55 to 59 | 7 |
| 62.5 | 1.965021378 | 1.915128433 | 2.01621413 | Global | Ischemic stroke | Deaths | Male | Low physical activity | 60 to 64 | 8 |
| 67.5 | 3.454668418 | 3.372237613 | 3.53911416 | Global | Ischemic stroke | Deaths | Male | Low physical activity | 65 to 69 | 9 |
| 72.5 | 6.574949432 | 6.398492636 | 6.75627253 | Global | Ischemic stroke | Deaths | Male | Low physical activity | 70 to 74 | 10 |
| 77.5 | 10.089901351 | 9.814287803 | 10.37325492 | Global | Ischemic stroke | Deaths | Male | Low physical activity | 75 to 79 | 11 |
| 82.5 | 37.816301023 | 36.804577206 | 38.85583619 | Global | Ischemic stroke | Deaths | Male | Low physical activity | 80 to 84 | 12 |
| 87.5 | 57.280758258 | 55.693358154 | 58.91340324 | Global | Ischemic stroke | Deaths | Male | Low physical activity | 85 to 89 | 13 |
| 92.5 | 71.276140572 | 69.103342671 | 73.51725718 | Global | Ischemic stroke | Deaths | Male | Low physical activity | 90 to 94 | 14 |
| 27.5 | 0.009416887 | 0.005739907 | 0.01544934 | World Bank High Income | Ischemic stroke | Deaths | Male | Low physical activity | 25 to 29 | 1 |
| 32.5 | 0.017287029 | 0.012054379 | 0.02479110 | World Bank High Income | Ischemic stroke | Deaths | Male | Low physical activity | 30 to 34 | 2 |
| 37.5 | 0.038030429 | 0.029364701 | 0.04925347 | World Bank High Income | Ischemic stroke | Deaths | Male | Low physical activity | 35 to 39 | 3 |
| 42.5 | 0.064705348 | 0.053178490 | 0.07873074 | World Bank High Income | Ischemic stroke | Deaths | Male | Low physical activity | 40 to 44 | 4 |
| 47.5 | 0.133784054 | 0.115812944 | 0.15454381 | World Bank High Income | Ischemic stroke | Deaths | Male | Low physical activity | 45 to 49 | 5 |
| 52.5 | 0.222354922 | 0.198073295 | 0.24961321 | World Bank High Income | Ischemic stroke | Deaths | Male | Low physical activity | 50 to 54 | 6 |
| 57.5 | 0.335255270 | 0.304016162 | 0.36970435 | World Bank High Income | Ischemic stroke | Deaths | Male | Low physical activity | 55 to 59 | 7 |
| 62.5 | 0.690914265 | 0.636333408 | 0.75017674 | World Bank High Income | Ischemic stroke | Deaths | Male | Low physical activity | 60 to 64 | 8 |
| 67.5 | 1.041275304 | 0.962775334 | 1.12617578 | World Bank High Income | Ischemic stroke | Deaths | Male | Low physical activity | 65 to 69 | 9 |
| 72.5 | 1.892461204 | 1.739824697 | 2.05848866 | World Bank High Income | Ischemic stroke | Deaths | Male | Low physical activity | 70 to 74 | 10 |
| 77.5 | 2.761405845 | 2.538459659 | 3.00393280 | World Bank High Income | Ischemic stroke | Deaths | Male | Low physical activity | 75 to 79 | 11 |
| 82.5 | 10.382765135 | 9.561872150 | 11.27413232 | World Bank High Income | Ischemic stroke | Deaths | Male | Low physical activity | 80 to 84 | 12 |
| 87.5 | 15.577793756 | 14.334326615 | 16.92912857 | World Bank High Income | Ischemic stroke | Deaths | Male | Low physical activity | 85 to 89 | 13 |
| 92.5 | 20.748087737 | 19.049024274 | 22.59869789 | World Bank High Income | Ischemic stroke | Deaths | Male | Low physical activity | 90 to 94 | 14 |
| 27.5 | 0.022056695 | 0.017438277 | 0.02789827 | World Bank Upper Middle Income | Ischemic stroke | Deaths | Male | Low physical activity | 25 to 29 | 1 |
| 32.5 | 0.039949259 | 0.033617748 | 0.04747324 | World Bank Upper Middle Income | Ischemic stroke | Deaths | Male | Low physical activity | 30 to 34 | 2 |
| 37.5 | 0.074957341 | 0.065980516 | 0.08515549 | World Bank Upper Middle Income | Ischemic stroke | Deaths | Male | Low physical activity | 35 to 39 | 3 |
| 42.5 | 0.139776261 | 0.127079638 | 0.15374141 | World Bank Upper Middle Income | Ischemic stroke | Deaths | Male | Low physical activity | 40 to 44 | 4 |
| 47.5 | 0.299672654 | 0.279533967 | 0.32126221 | World Bank Upper Middle Income | Ischemic stroke | Deaths | Male | Low physical activity | 45 to 49 | 5 |
| 52.5 | 0.549457469 | 0.520310726 | 0.58023695 | World Bank Upper Middle Income | Ischemic stroke | Deaths | Male | Low physical activity | 50 to 54 | 6 |
| 57.5 | 0.965459185 | 0.922950795 | 1.00992539 | World Bank Upper Middle Income | Ischemic stroke | Deaths | Male | Low physical activity | 55 to 59 | 7 |
| 62.5 | 2.320770925 | 2.238677717 | 2.40587452 | World Bank Upper Middle Income | Ischemic stroke | Deaths | Male | Low physical activity | 60 to 64 | 8 |
| 67.5 | 4.174616260 | 4.036613745 | 4.31733676 | World Bank Upper Middle Income | Ischemic stroke | Deaths | Male | Low physical activity | 65 to 69 | 9 |
| 72.5 | 8.088345495 | 7.788331900 | 8.39991589 | World Bank Upper Middle Income | Ischemic stroke | Deaths | Male | Low physical activity | 70 to 74 | 10 |
| 77.5 | 13.085185158 | 12.592837287 | 13.59678258 | World Bank Upper Middle Income | Ischemic stroke | Deaths | Male | Low physical activity | 75 to 79 | 11 |
| 82.5 | 50.924287245 | 49.043271806 | 52.87744752 | World Bank Upper Middle Income | Ischemic stroke | Deaths | Male | Low physical activity | 80 to 84 | 12 |
| 87.5 | 84.586861647 | 81.335228165 | 87.96848948 | World Bank Upper Middle Income | Ischemic stroke | Deaths | Male | Low physical activity | 85 to 89 | 13 |
| 92.5 | 104.152170483 | 99.583986971 | 108.92990878 | World Bank Upper Middle Income | Ischemic stroke | Deaths | Male | Low physical activity | 90 to 94 | 14 |
| 27.5 | 0.017598913 | 0.013946784 | 0.02220739 | World Bank Lower Middle Income | Ischemic stroke | Deaths | Male | Low physical activity | 25 to 29 | 1 |
| 32.5 | 0.024874439 | 0.020496536 | 0.03018743 | World Bank Lower Middle Income | Ischemic stroke | Deaths | Male | Low physical activity | 30 to 34 | 2 |
| 37.5 | 0.042986561 | 0.036730064 | 0.05030877 | World Bank Lower Middle Income | Ischemic stroke | Deaths | Male | Low physical activity | 35 to 39 | 3 |
| 42.5 | 0.073410729 | 0.064612670 | 0.08340679 | World Bank Lower Middle Income | Ischemic stroke | Deaths | Male | Low physical activity | 40 to 44 | 4 |
| 47.5 | 0.180360957 | 0.164272510 | 0.19802507 | World Bank Lower Middle Income | Ischemic stroke | Deaths | Male | Low physical activity | 45 to 49 | 5 |
| 52.5 | 0.428928611 | 0.399927409 | 0.46003287 | World Bank Lower Middle Income | Ischemic stroke | Deaths | Male | Low physical activity | 50 to 54 | 6 |
| 57.5 | 0.830119670 | 0.783991104 | 0.87896235 | World Bank Lower Middle Income | Ischemic stroke | Deaths | Male | Low physical activity | 55 to 59 | 7 |
| 62.5 | 2.581716075 | 2.473188065 | 2.69500649 | World Bank Lower Middle Income | Ischemic stroke | Deaths | Male | Low physical activity | 60 to 64 | 8 |
| 67.5 | 4.744408424 | 4.556022856 | 4.94058349 | World Bank Lower Middle Income | Ischemic stroke | Deaths | Male | Low physical activity | 65 to 69 | 9 |
| 72.5 | 9.311431695 | 8.887517198 | 9.75556596 | World Bank Lower Middle Income | Ischemic stroke | Deaths | Male | Low physical activity | 70 to 74 | 10 |
| 77.5 | 14.212327422 | 13.542779745 | 14.91497717 | World Bank Lower Middle Income | Ischemic stroke | Deaths | Male | Low physical activity | 75 to 79 | 11 |
| 82.5 | 54.229743876 | 51.719409846 | 56.86192340 | World Bank Lower Middle Income | Ischemic stroke | Deaths | Male | Low physical activity | 80 to 84 | 12 |
| 87.5 | 81.200037035 | 77.224193391 | 85.38057473 | World Bank Lower Middle Income | Ischemic stroke | Deaths | Male | Low physical activity | 85 to 89 | 13 |
| 92.5 | 114.439541198 | 108.052607478 | 121.20400327 | World Bank Lower Middle Income | Ischemic stroke | Deaths | Male | Low physical activity | 90 to 94 | 14 |
| 27.5 | 0.012910856 | 0.006530970 | 0.02552304 | World Bank Low Income | Ischemic stroke | Deaths | Male | Low physical activity | 25 to 29 | 1 |
| 32.5 | 0.017432731 | 0.009618595 | 0.03159506 | World Bank Low Income | Ischemic stroke | Deaths | Male | Low physical activity | 30 to 34 | 2 |
| 37.5 | 0.029568778 | 0.018048333 | 0.04844285 | World Bank Low Income | Ischemic stroke | Deaths | Male | Low physical activity | 35 to 39 | 3 |
| 42.5 | 0.062835604 | 0.042842755 | 0.09215825 | World Bank Low Income | Ischemic stroke | Deaths | Male | Low physical activity | 40 to 44 | 4 |
| 47.5 | 0.137413916 | 0.101954112 | 0.18520670 | World Bank Low Income | Ischemic stroke | Deaths | Male | Low physical activity | 45 to 49 | 5 |
| 52.5 | 0.303528107 | 0.240912903 | 0.38241751 | World Bank Low Income | Ischemic stroke | Deaths | Male | Low physical activity | 50 to 54 | 6 |
| 57.5 | 0.569160064 | 0.469910108 | 0.68937265 | World Bank Low Income | Ischemic stroke | Deaths | Male | Low physical activity | 55 to 59 | 7 |
| 62.5 | 1.778549352 | 1.535273087 | 2.06037468 | World Bank Low Income | Ischemic stroke | Deaths | Male | Low physical activity | 60 to 64 | 8 |
| 67.5 | 3.133253608 | 2.724420662 | 3.60343698 | World Bank Low Income | Ischemic stroke | Deaths | Male | Low physical activity | 65 to 69 | 9 |
| 72.5 | 5.854195916 | 4.985642495 | 6.87406084 | World Bank Low Income | Ischemic stroke | Deaths | Male | Low physical activity | 70 to 74 | 10 |
| 77.5 | 9.055970846 | 7.662951083 | 10.70222256 | World Bank Low Income | Ischemic stroke | Deaths | Male | Low physical activity | 75 to 79 | 11 |
| 82.5 | 34.441644281 | 29.229165852 | 40.58367135 | World Bank Low Income | Ischemic stroke | Deaths | Male | Low physical activity | 80 to 84 | 12 |
| 87.5 | 49.185723592 | 41.217474593 | 58.69441127 | World Bank Low Income | Ischemic stroke | Deaths | Male | Low physical activity | 85 to 89 | 13 |
| 92.5 | 69.608476832 | 56.463508496 | 85.81365515 | World Bank Low Income | Ischemic stroke | Deaths | Male | Low physical activity | 90 to 94 | 14 |
| 27.5 | 0.024574538 | 0.021322237 | 0.02832292 | Global | Ischemic stroke | Deaths | Female | Low physical activity | 25 to 29 | 1 |
| 32.5 | 0.036736713 | 0.032752167 | 0.04120601 | Global | Ischemic stroke | Deaths | Female | Low physical activity | 30 to 34 | 2 |
| 37.5 | 0.060989556 | 0.055634730 | 0.06685978 | Global | Ischemic stroke | Deaths | Female | Low physical activity | 35 to 39 | 3 |
| 42.5 | 0.097633334 | 0.090655664 | 0.10514807 | Global | Ischemic stroke | Deaths | Female | Low physical activity | 40 to 44 | 4 |
| 47.5 | 0.167844356 | 0.158247319 | 0.17802341 | Global | Ischemic stroke | Deaths | Female | Low physical activity | 45 to 49 | 5 |
| 52.5 | 0.309606408 | 0.295536764 | 0.32434587 | Global | Ischemic stroke | Deaths | Female | Low physical activity | 50 to 54 | 6 |
| 57.5 | 0.534167689 | 0.514173887 | 0.55493896 | Global | Ischemic stroke | Deaths | Female | Low physical activity | 55 to 59 | 7 |
| 62.5 | 1.381209296 | 1.340908611 | 1.42272121 | Global | Ischemic stroke | Deaths | Female | Low physical activity | 60 to 64 | 8 |
| 67.5 | 2.533784511 | 2.465456930 | 2.60400572 | Global | Ischemic stroke | Deaths | Female | Low physical activity | 65 to 69 | 9 |
| 72.5 | 5.691578627 | 5.522864747 | 5.86544642 | Global | Ischemic stroke | Deaths | Female | Low physical activity | 70 to 74 | 10 |
| 77.5 | 9.476370461 | 9.194506074 | 9.76687561 | Global | Ischemic stroke | Deaths | Female | Low physical activity | 75 to 79 | 11 |
| 82.5 | 38.095509946 | 36.980499347 | 39.24413958 | Global | Ischemic stroke | Deaths | Female | Low physical activity | 80 to 84 | 12 |
| 87.5 | 59.369068138 | 57.609578013 | 61.18229595 | Global | Ischemic stroke | Deaths | Female | Low physical activity | 85 to 89 | 13 |
| 92.5 | 82.763350684 | 80.251998469 | 85.35329147 | Global | Ischemic stroke | Deaths | Female | Low physical activity | 90 to 94 | 14 |
| 27.5 | 0.013917866 | 0.008983893 | 0.02156159 | World Bank High Income | Ischemic stroke | Deaths | Female | Low physical activity | 25 to 29 | 1 |
| 32.5 | 0.023616595 | 0.016875697 | 0.03305010 | World Bank High Income | Ischemic stroke | Deaths | Female | Low physical activity | 30 to 34 | 2 |
| 37.5 | 0.040070487 | 0.030683139 | 0.05232985 | World Bank High Income | Ischemic stroke | Deaths | Female | Low physical activity | 35 to 39 | 3 |
| 42.5 | 0.058982692 | 0.047502215 | 0.07323780 | World Bank High Income | Ischemic stroke | Deaths | Female | Low physical activity | 40 to 44 | 4 |
| 47.5 | 0.085782856 | 0.071794026 | 0.10249736 | World Bank High Income | Ischemic stroke | Deaths | Female | Low physical activity | 45 to 49 | 5 |
| 52.5 | 0.126209657 | 0.108857903 | 0.14632725 | World Bank High Income | Ischemic stroke | Deaths | Female | Low physical activity | 50 to 54 | 6 |
| 57.5 | 0.181139653 | 0.159765977 | 0.20537273 | World Bank High Income | Ischemic stroke | Deaths | Female | Low physical activity | 55 to 59 | 7 |
| 62.5 | 0.404398365 | 0.365373811 | 0.44759102 | World Bank High Income | Ischemic stroke | Deaths | Female | Low physical activity | 60 to 64 | 8 |
| 67.5 | 0.653489442 | 0.593830984 | 0.71914141 | World Bank High Income | Ischemic stroke | Deaths | Female | Low physical activity | 65 to 69 | 9 |
| 72.5 | 1.532475539 | 1.386286319 | 1.69408097 | World Bank High Income | Ischemic stroke | Deaths | Female | Low physical activity | 70 to 74 | 10 |
| 77.5 | 2.625062478 | 2.376290117 | 2.89987867 | World Bank High Income | Ischemic stroke | Deaths | Female | Low physical activity | 75 to 79 | 11 |
| 82.5 | 11.927065645 | 10.809674981 | 13.15996042 | World Bank High Income | Ischemic stroke | Deaths | Female | Low physical activity | 80 to 84 | 12 |
| 87.5 | 20.139910054 | 18.250816826 | 22.22453827 | World Bank High Income | Ischemic stroke | Deaths | Female | Low physical activity | 85 to 89 | 13 |
| 92.5 | 30.092965730 | 27.260282592 | 33.21999995 | World Bank High Income | Ischemic stroke | Deaths | Female | Low physical activity | 90 to 94 | 14 |
| 27.5 | 0.040664956 | 0.030349326 | 0.05448683 | World Bank Upper Middle Income | Ischemic stroke | Deaths | Female | Low physical activity | 25 to 29 | 1 |
| 32.5 | 0.056137748 | 0.044374956 | 0.07101859 | World Bank Upper Middle Income | Ischemic stroke | Deaths | Female | Low physical activity | 30 to 34 | 2 |
| 37.5 | 0.088300839 | 0.073458942 | 0.10614145 | World Bank Upper Middle Income | Ischemic stroke | Deaths | Female | Low physical activity | 35 to 39 | 3 |
| 42.5 | 0.134378218 | 0.116179003 | 0.15542830 | World Bank Upper Middle Income | Ischemic stroke | Deaths | Female | Low physical activity | 40 to 44 | 4 |
| 47.5 | 0.232634686 | 0.208027617 | 0.26015246 | World Bank Upper Middle Income | Ischemic stroke | Deaths | Female | Low physical activity | 45 to 49 | 5 |
| 52.5 | 0.403656912 | 0.369685682 | 0.44074983 | World Bank Upper Middle Income | Ischemic stroke | Deaths | Female | Low physical activity | 50 to 54 | 6 |
| 57.5 | 0.686945841 | 0.639375321 | 0.73805568 | World Bank Upper Middle Income | Ischemic stroke | Deaths | Female | Low physical activity | 55 to 59 | 7 |
| 62.5 | 1.612567317 | 1.522862456 | 1.70755628 | World Bank Upper Middle Income | Ischemic stroke | Deaths | Female | Low physical activity | 60 to 64 | 8 |
| 67.5 | 2.991069866 | 2.837649316 | 3.15278526 | World Bank Upper Middle Income | Ischemic stroke | Deaths | Female | Low physical activity | 65 to 69 | 9 |
| 72.5 | 6.495190949 | 6.131151883 | 6.88084495 | World Bank Upper Middle Income | Ischemic stroke | Deaths | Female | Low physical activity | 70 to 74 | 10 |
| 77.5 | 11.137457956 | 10.512861815 | 11.79916296 | World Bank Upper Middle Income | Ischemic stroke | Deaths | Female | Low physical activity | 75 to 79 | 11 |
| 82.5 | 46.129828504 | 43.584126871 | 48.82422181 | World Bank Upper Middle Income | Ischemic stroke | Deaths | Female | Low physical activity | 80 to 84 | 12 |
| 87.5 | 71.813884454 | 67.785560890 | 76.08160105 | World Bank Upper Middle Income | Ischemic stroke | Deaths | Female | Low physical activity | 85 to 89 | 13 |
| 92.5 | 96.160543175 | 90.566886272 | 102.09967952 | World Bank Upper Middle Income | Ischemic stroke | Deaths | Female | Low physical activity | 90 to 94 | 14 |
| 27.5 | 0.017795463 | 0.014124813 | 0.02242001 | World Bank Lower Middle Income | Ischemic stroke | Deaths | Female | Low physical activity | 25 to 29 | 1 |
| 32.5 | 0.026980000 | 0.022248570 | 0.03271763 | World Bank Lower Middle Income | Ischemic stroke | Deaths | Female | Low physical activity | 30 to 34 | 2 |
| 37.5 | 0.046740435 | 0.039911406 | 0.05473794 | World Bank Lower Middle Income | Ischemic stroke | Deaths | Female | Low physical activity | 35 to 39 | 3 |
| 42.5 | 0.080666734 | 0.070837114 | 0.09186035 | World Bank Lower Middle Income | Ischemic stroke | Deaths | Female | Low physical activity | 40 to 44 | 4 |
| 47.5 | 0.147882479 | 0.133066066 | 0.16434864 | World Bank Lower Middle Income | Ischemic stroke | Deaths | Female | Low physical activity | 45 to 49 | 5 |
| 52.5 | 0.319353980 | 0.294119206 | 0.34675384 | World Bank Lower Middle Income | Ischemic stroke | Deaths | Female | Low physical activity | 50 to 54 | 6 |
| 57.5 | 0.601101676 | 0.562183875 | 0.64271360 | World Bank Lower Middle Income | Ischemic stroke | Deaths | Female | Low physical activity | 55 to 59 | 7 |
| 62.5 | 1.899276945 | 1.808141744 | 1.99500560 | World Bank Lower Middle Income | Ischemic stroke | Deaths | Female | Low physical activity | 60 to 64 | 8 |
| 67.5 | 3.698429261 | 3.534267297 | 3.87021633 | World Bank Lower Middle Income | Ischemic stroke | Deaths | Female | Low physical activity | 65 to 69 | 9 |
| 72.5 | 9.090420070 | 8.634982170 | 9.56987929 | World Bank Lower Middle Income | Ischemic stroke | Deaths | Female | Low physical activity | 70 to 74 | 10 |
| 77.5 | 15.420404630 | 14.632312494 | 16.25094318 | World Bank Lower Middle Income | Ischemic stroke | Deaths | Female | Low physical activity | 75 to 79 | 11 |
| 82.5 | 57.682352440 | 54.758291673 | 60.76255634 | World Bank Lower Middle Income | Ischemic stroke | Deaths | Female | Low physical activity | 80 to 84 | 12 |
| 87.5 | 89.534629537 | 84.843675992 | 94.48494296 | World Bank Lower Middle Income | Ischemic stroke | Deaths | Female | Low physical activity | 85 to 89 | 13 |
| 92.5 | 125.763613208 | 118.671080583 | 133.28004034 | World Bank Lower Middle Income | Ischemic stroke | Deaths | Female | Low physical activity | 90 to 94 | 14 |
| 27.5 | 0.017151245 | 0.009669072 | 0.03042332 | World Bank Low Income | Ischemic stroke | Deaths | Female | Low physical activity | 25 to 29 | 1 |
| 32.5 | 0.032840703 | 0.020759587 | 0.05195247 | World Bank Low Income | Ischemic stroke | Deaths | Female | Low physical activity | 30 to 34 | 2 |
| 37.5 | 0.055276697 | 0.037691924 | 0.08106546 | World Bank Low Income | Ischemic stroke | Deaths | Female | Low physical activity | 35 to 39 | 3 |
| 42.5 | 0.100508607 | 0.073278267 | 0.13785779 | World Bank Low Income | Ischemic stroke | Deaths | Female | Low physical activity | 40 to 44 | 4 |
| 47.5 | 0.166105766 | 0.126709511 | 0.21775102 | World Bank Low Income | Ischemic stroke | Deaths | Female | Low physical activity | 45 to 49 | 5 |
| 52.5 | 0.334444940 | 0.269190787 | 0.41551726 | World Bank Low Income | Ischemic stroke | Deaths | Female | Low physical activity | 50 to 54 | 6 |
| 57.5 | 0.567762451 | 0.470849694 | 0.68462230 | World Bank Low Income | Ischemic stroke | Deaths | Female | Low physical activity | 55 to 59 | 7 |
| 62.5 | 1.588301780 | 1.371939068 | 1.83878614 | World Bank Low Income | Ischemic stroke | Deaths | Female | Low physical activity | 60 to 64 | 8 |
| 67.5 | 2.953487319 | 2.575853274 | 3.38648456 | World Bank Low Income | Ischemic stroke | Deaths | Female | Low physical activity | 65 to 69 | 9 |
| 72.5 | 7.000947599 | 6.006671637 | 8.15980467 | World Bank Low Income | Ischemic stroke | Deaths | Female | Low physical activity | 70 to 74 | 10 |
| 77.5 | 11.613552750 | 9.926854410 | 13.58684251 | World Bank Low Income | Ischemic stroke | Deaths | Female | Low physical activity | 75 to 79 | 11 |
| 82.5 | 42.588320765 | 36.457515235 | 49.75010101 | World Bank Low Income | Ischemic stroke | Deaths | Female | Low physical activity | 80 to 84 | 12 |
| 87.5 | 58.120291943 | 49.372096055 | 68.41857254 | World Bank Low Income | Ischemic stroke | Deaths | Female | Low physical activity | 85 to 89 | 13 |
| 92.5 | 80.970412809 | 67.660886237 | 96.89804723 | World Bank Low Income | Ischemic stroke | Deaths | Female | Low physical activity | 90 to 94 | 14 |

**Supplementary Table 7. Period RR results of Age-Period-Cohort analysis**

| Period | Rate Ratio | CI Lo | CI Hi | location | cause | measure | sex | rei | label | x |
| --- | --- | --- | --- | --- | --- | --- | --- | --- | --- | --- |
| 1,992.5 | 1.0433840 | 1.0301399 | 1.0567984 | Global | Ischemic stroke | Deaths | Both | Low physical activity | 1990 to 1994 | 1 |
| 1,997.5 | 1.0301044 | 1.0200704 | 1.0402371 | Global | Ischemic stroke | Deaths | Both | Low physical activity | 1995 to 1999 | 2 |
| 2,002.5 | 1.0000000 | 1.0000000 | 1.0000000 | Global | Ischemic stroke | Deaths | Both | Low physical activity | 2000 to 2004 | 3 |
| 2,007.5 | 0.9146400 | 0.9060687 | 0.9232923 | Global | Ischemic stroke | Deaths | Both | Low physical activity | 2005 to 2009 | 4 |
| 2,012.5 | 0.8471328 | 0.8374698 | 0.8569073 | Global | Ischemic stroke | Deaths | Both | Low physical activity | 2010 to 2014 | 5 |
| 2,017.5 | 0.8151874 | 0.8038885 | 0.8266452 | Global | Ischemic stroke | Deaths | Both | Low physical activity | 2015 to 2019 | 6 |
| 1,992.5 | 1.3411736 | 1.2934978 | 1.3906067 | World Bank High Income | Ischemic stroke | Deaths | Both | Low physical activity | 1990 to 1994 | 1 |
| 1,997.5 | 1.1762617 | 1.1480416 | 1.2051755 | World Bank High Income | Ischemic stroke | Deaths | Both | Low physical activity | 1995 to 1999 | 2 |
| 2,002.5 | 1.0000000 | 1.0000000 | 1.0000000 | World Bank High Income | Ischemic stroke | Deaths | Both | Low physical activity | 2000 to 2004 | 3 |
| 2,007.5 | 0.8314025 | 0.8111149 | 0.8521975 | World Bank High Income | Ischemic stroke | Deaths | Both | Low physical activity | 2005 to 2009 | 4 |
| 2,012.5 | 0.7488386 | 0.7229669 | 0.7756360 | World Bank High Income | Ischemic stroke | Deaths | Both | Low physical activity | 2010 to 2014 | 5 |
| 2,017.5 | 0.7594573 | 0.7248391 | 0.7957289 | World Bank High Income | Ischemic stroke | Deaths | Both | Low physical activity | 2015 to 2019 | 6 |
| 1,992.5 | 1.0931176 | 1.0666922 | 1.1201977 | World Bank Upper Middle Income | Ischemic stroke | Deaths | Both | Low physical activity | 1990 to 1994 | 1 |
| 1,997.5 | 1.0449727 | 1.0255934 | 1.0647183 | World Bank Upper Middle Income | Ischemic stroke | Deaths | Both | Low physical activity | 1995 to 1999 | 2 |
| 2,002.5 | 1.0000000 | 1.0000000 | 1.0000000 | World Bank Upper Middle Income | Ischemic stroke | Deaths | Both | Low physical activity | 2000 to 2004 | 3 |
| 2,007.5 | 0.8787797 | 0.8633794 | 0.8944547 | World Bank Upper Middle Income | Ischemic stroke | Deaths | Both | Low physical activity | 2005 to 2009 | 4 |
| 2,012.5 | 0.7954800 | 0.7783217 | 0.8130165 | World Bank Upper Middle Income | Ischemic stroke | Deaths | Both | Low physical activity | 2010 to 2014 | 5 |
| 2,017.5 | 0.7337794 | 0.7142557 | 0.7538367 | World Bank Upper Middle Income | Ischemic stroke | Deaths | Both | Low physical activity | 2015 to 2019 | 6 |
| 1,992.5 | 0.9079825 | 0.8849480 | 0.9316167 | World Bank Lower Middle Income | Ischemic stroke | Deaths | Both | Low physical activity | 1990 to 1994 | 1 |
| 1,997.5 | 0.9584753 | 0.9387064 | 0.9786606 | World Bank Lower Middle Income | Ischemic stroke | Deaths | Both | Low physical activity | 1995 to 1999 | 2 |
| 2,002.5 | 1.0000000 | 1.0000000 | 1.0000000 | World Bank Lower Middle Income | Ischemic stroke | Deaths | Both | Low physical activity | 2000 to 2004 | 3 |
| 2,007.5 | 1.0134988 | 0.9941382 | 1.0332364 | World Bank Lower Middle Income | Ischemic stroke | Deaths | Both | Low physical activity | 2005 to 2009 | 4 |
| 2,012.5 | 0.9601938 | 0.9395410 | 0.9813006 | World Bank Lower Middle Income | Ischemic stroke | Deaths | Both | Low physical activity | 2010 to 2014 | 5 |
| 2,017.5 | 0.9421408 | 0.9190286 | 0.9658343 | World Bank Lower Middle Income | Ischemic stroke | Deaths | Both | Low physical activity | 2015 to 2019 | 6 |
| 1,992.5 | 0.9550872 | 0.8803536 | 1.0361651 | World Bank Low Income | Ischemic stroke | Deaths | Both | Low physical activity | 1990 to 1994 | 1 |
| 1,997.5 | 0.9704892 | 0.9056304 | 1.0399930 | World Bank Low Income | Ischemic stroke | Deaths | Both | Low physical activity | 1995 to 1999 | 2 |
| 2,002.5 | 1.0000000 | 1.0000000 | 1.0000000 | World Bank Low Income | Ischemic stroke | Deaths | Both | Low physical activity | 2000 to 2004 | 3 |
| 2,007.5 | 1.0250607 | 0.9620530 | 1.0921949 | World Bank Low Income | Ischemic stroke | Deaths | Both | Low physical activity | 2005 to 2009 | 4 |
| 2,012.5 | 1.0199130 | 0.9529464 | 1.0915856 | World Bank Low Income | Ischemic stroke | Deaths | Both | Low physical activity | 2010 to 2014 | 5 |
| 2,017.5 | 1.0108879 | 0.9389369 | 1.0883525 | World Bank Low Income | Ischemic stroke | Deaths | Both | Low physical activity | 2015 to 2019 | 6 |
| 1,992.5 | 1.0551157 | 1.0347879 | 1.0758428 | Global | Ischemic stroke | Deaths | Male | Low physical activity | 1990 to 1994 | 1 |
| 1,997.5 | 1.0359912 | 1.0198861 | 1.0523505 | Global | Ischemic stroke | Deaths | Male | Low physical activity | 1995 to 1999 | 2 |
| 2,002.5 | 1.0000000 | 1.0000000 | 1.0000000 | Global | Ischemic stroke | Deaths | Male | Low physical activity | 2000 to 2004 | 3 |
| 2,007.5 | 0.9387525 | 0.9248498 | 0.9528642 | Global | Ischemic stroke | Deaths | Male | Low physical activity | 2005 to 2009 | 4 |
| 2,012.5 | 0.8898045 | 0.8746195 | 0.9052532 | Global | Ischemic stroke | Deaths | Male | Low physical activity | 2010 to 2014 | 5 |
| 2,017.5 | 0.8670357 | 0.8497872 | 0.8846343 | Global | Ischemic stroke | Deaths | Male | Low physical activity | 2015 to 2019 | 6 |
| 1,992.5 | 1.2993990 | 1.2409041 | 1.3606513 | World Bank High Income | Ischemic stroke | Deaths | Male | Low physical activity | 1990 to 1994 | 1 |
| 1,997.5 | 1.1643942 | 1.1254637 | 1.2046713 | World Bank High Income | Ischemic stroke | Deaths | Male | Low physical activity | 1995 to 1999 | 2 |
| 2,002.5 | 1.0000000 | 1.0000000 | 1.0000000 | World Bank High Income | Ischemic stroke | Deaths | Male | Low physical activity | 2000 to 2004 | 3 |
| 2,007.5 | 0.8399448 | 0.8113497 | 0.8695477 | World Bank High Income | Ischemic stroke | Deaths | Male | Low physical activity | 2005 to 2009 | 4 |
| 2,012.5 | 0.7452918 | 0.7127888 | 0.7792769 | World Bank High Income | Ischemic stroke | Deaths | Male | Low physical activity | 2010 to 2014 | 5 |
| 2,017.5 | 0.7636812 | 0.7224246 | 0.8072940 | World Bank High Income | Ischemic stroke | Deaths | Male | Low physical activity | 2015 to 2019 | 6 |
| 1,992.5 | 1.1163041 | 1.0852455 | 1.1482516 | World Bank Upper Middle Income | Ischemic stroke | Deaths | Male | Low physical activity | 1990 to 1994 | 1 |
| 1,997.5 | 1.0512426 | 1.0277027 | 1.0753217 | World Bank Upper Middle Income | Ischemic stroke | Deaths | Male | Low physical activity | 1995 to 1999 | 2 |
| 2,002.5 | 1.0000000 | 1.0000000 | 1.0000000 | World Bank Upper Middle Income | Ischemic stroke | Deaths | Male | Low physical activity | 2000 to 2004 | 3 |
| 2,007.5 | 0.9120209 | 0.8928741 | 0.9315784 | World Bank Upper Middle Income | Ischemic stroke | Deaths | Male | Low physical activity | 2005 to 2009 | 4 |
| 2,012.5 | 0.8697740 | 0.8485456 | 0.8915334 | World Bank Upper Middle Income | Ischemic stroke | Deaths | Male | Low physical activity | 2010 to 2014 | 5 |
| 2,017.5 | 0.8263176 | 0.8024173 | 0.8509298 | World Bank Upper Middle Income | Ischemic stroke | Deaths | Male | Low physical activity | 2015 to 2019 | 6 |
| 1,992.5 | 0.9262256 | 0.8926191 | 0.9610973 | World Bank Lower Middle Income | Ischemic stroke | Deaths | Male | Low physical activity | 1990 to 1994 | 1 |
| 1,997.5 | 0.9752197 | 0.9458923 | 1.0054564 | World Bank Lower Middle Income | Ischemic stroke | Deaths | Male | Low physical activity | 1995 to 1999 | 2 |
| 2,002.5 | 1.0000000 | 1.0000000 | 1.0000000 | World Bank Lower Middle Income | Ischemic stroke | Deaths | Male | Low physical activity | 2000 to 2004 | 3 |
| 2,007.5 | 1.0299674 | 1.0012418 | 1.0595172 | World Bank Lower Middle Income | Ischemic stroke | Deaths | Male | Low physical activity | 2005 to 2009 | 4 |
| 2,012.5 | 0.9825560 | 0.9522427 | 1.0138343 | World Bank Lower Middle Income | Ischemic stroke | Deaths | Male | Low physical activity | 2010 to 2014 | 5 |
| 2,017.5 | 0.9597856 | 0.9265904 | 0.9941699 | World Bank Lower Middle Income | Ischemic stroke | Deaths | Male | Low physical activity | 2015 to 2019 | 6 |
| 1,992.5 | 0.9419335 | 0.8304905 | 1.0683310 | World Bank Low Income | Ischemic stroke | Deaths | Male | Low physical activity | 1990 to 1994 | 1 |
| 1,997.5 | 0.9686020 | 0.8696669 | 1.0787922 | World Bank Low Income | Ischemic stroke | Deaths | Male | Low physical activity | 1995 to 1999 | 2 |
| 2,002.5 | 1.0000000 | 1.0000000 | 1.0000000 | World Bank Low Income | Ischemic stroke | Deaths | Male | Low physical activity | 2000 to 2004 | 3 |
| 2,007.5 | 1.0291467 | 0.9313276 | 1.1372400 | World Bank Low Income | Ischemic stroke | Deaths | Male | Low physical activity | 2005 to 2009 | 4 |
| 2,012.5 | 1.0281355 | 0.9241664 | 1.1438012 | World Bank Low Income | Ischemic stroke | Deaths | Male | Low physical activity | 2010 to 2014 | 5 |
| 2,017.5 | 1.0223151 | 0.9114430 | 1.1466743 | World Bank Low Income | Ischemic stroke | Deaths | Male | Low physical activity | 2015 to 2019 | 6 |
| 1,992.5 | 1.0535837 | 1.0354995 | 1.0719838 | Global | Ischemic stroke | Deaths | Female | Low physical activity | 1990 to 1994 | 1 |
| 1,997.5 | 1.0337110 | 1.0206519 | 1.0469371 | Global | Ischemic stroke | Deaths | Female | Low physical activity | 1995 to 1999 | 2 |
| 2,002.5 | 1.0000000 | 1.0000000 | 1.0000000 | Global | Ischemic stroke | Deaths | Female | Low physical activity | 2000 to 2004 | 3 |
| 2,007.5 | 0.8963837 | 0.8854230 | 0.9074800 | Global | Ischemic stroke | Deaths | Female | Low physical activity | 2005 to 2009 | 4 |
| 2,012.5 | 0.8162777 | 0.8035123 | 0.8292459 | Global | Ischemic stroke | Deaths | Female | Low physical activity | 2010 to 2014 | 5 |
| 2,017.5 | 0.7793642 | 0.7640488 | 0.7949867 | Global | Ischemic stroke | Deaths | Female | Low physical activity | 2015 to 2019 | 6 |
| 1,992.5 | 1.3796188 | 1.3238590 | 1.4377271 | World Bank High Income | Ischemic stroke | Deaths | Female | Low physical activity | 1990 to 1994 | 1 |
| 1,997.5 | 1.1893859 | 1.1588907 | 1.2206836 | World Bank High Income | Ischemic stroke | Deaths | Female | Low physical activity | 1995 to 1999 | 2 |
| 2,002.5 | 1.0000000 | 1.0000000 | 1.0000000 | World Bank High Income | Ischemic stroke | Deaths | Female | Low physical activity | 2000 to 2004 | 3 |
| 2,007.5 | 0.8241517 | 0.8027086 | 0.8461677 | World Bank High Income | Ischemic stroke | Deaths | Female | Low physical activity | 2005 to 2009 | 4 |
| 2,012.5 | 0.7458931 | 0.7164186 | 0.7765804 | World Bank High Income | Ischemic stroke | Deaths | Female | Low physical activity | 2010 to 2014 | 5 |
| 2,017.5 | 0.7561964 | 0.7154167 | 0.7993006 | World Bank High Income | Ischemic stroke | Deaths | Female | Low physical activity | 2015 to 2019 | 6 |
| 1,992.5 | 1.1091829 | 1.0704888 | 1.1492756 | World Bank Upper Middle Income | Ischemic stroke | Deaths | Female | Low physical activity | 1990 to 1994 | 1 |
| 1,997.5 | 1.0547893 | 1.0277158 | 1.0825760 | World Bank Upper Middle Income | Ischemic stroke | Deaths | Female | Low physical activity | 1995 to 1999 | 2 |
| 2,002.5 | 1.0000000 | 1.0000000 | 1.0000000 | World Bank Upper Middle Income | Ischemic stroke | Deaths | Female | Low physical activity | 2000 to 2004 | 3 |
| 2,007.5 | 0.8479887 | 0.8272916 | 0.8692035 | World Bank Upper Middle Income | Ischemic stroke | Deaths | Female | Low physical activity | 2005 to 2009 | 4 |
| 2,012.5 | 0.7340957 | 0.7108359 | 0.7581166 | World Bank Upper Middle Income | Ischemic stroke | Deaths | Female | Low physical activity | 2010 to 2014 | 5 |
| 2,017.5 | 0.6587832 | 0.6321659 | 0.6865213 | World Bank Upper Middle Income | Ischemic stroke | Deaths | Female | Low physical activity | 2015 to 2019 | 6 |
| 1,992.5 | 0.8918737 | 0.8604098 | 0.9244881 | World Bank Lower Middle Income | Ischemic stroke | Deaths | Female | Low physical activity | 1990 to 1994 | 1 |
| 1,997.5 | 0.9437857 | 0.9171795 | 0.9711638 | World Bank Lower Middle Income | Ischemic stroke | Deaths | Female | Low physical activity | 1995 to 1999 | 2 |
| 2,002.5 | 1.0000000 | 1.0000000 | 1.0000000 | World Bank Lower Middle Income | Ischemic stroke | Deaths | Female | Low physical activity | 2000 to 2004 | 3 |
| 2,007.5 | 1.0005650 | 0.9744537 | 1.0273760 | World Bank Lower Middle Income | Ischemic stroke | Deaths | Female | Low physical activity | 2005 to 2009 | 4 |
| 2,012.5 | 0.9426372 | 0.9144105 | 0.9717353 | World Bank Lower Middle Income | Ischemic stroke | Deaths | Female | Low physical activity | 2010 to 2014 | 5 |
| 2,017.5 | 0.9287237 | 0.8964581 | 0.9621507 | World Bank Lower Middle Income | Ischemic stroke | Deaths | Female | Low physical activity | 2015 to 2019 | 6 |
| 1,992.5 | 0.9742309 | 0.8756644 | 1.0838922 | World Bank Low Income | Ischemic stroke | Deaths | Female | Low physical activity | 1990 to 1994 | 1 |
| 1,997.5 | 0.9767784 | 0.8923387 | 1.0692085 | World Bank Low Income | Ischemic stroke | Deaths | Female | Low physical activity | 1995 to 1999 | 2 |
| 2,002.5 | 1.0000000 | 1.0000000 | 1.0000000 | World Bank Low Income | Ischemic stroke | Deaths | Female | Low physical activity | 2000 to 2004 | 3 |
| 2,007.5 | 1.0239419 | 0.9430816 | 1.1117352 | World Bank Low Income | Ischemic stroke | Deaths | Female | Low physical activity | 2005 to 2009 | 4 |
| 2,012.5 | 1.0150597 | 0.9291493 | 1.1089134 | World Bank Low Income | Ischemic stroke | Deaths | Female | Low physical activity | 2010 to 2014 | 5 |
| 2,017.5 | 1.0034144 | 0.9106786 | 1.1055935 | World Bank Low Income | Ischemic stroke | Deaths | Female | Low physical activity | 2015 to 2019 | 6 |

**Supplementary Table 8. Cohort RR results of Age-Period-Cohort analysis**

| Cohort | Rate Ratio | CI Lo | CI Hi | location | cause | measure | sex | rei | label | x |
| --- | --- | --- | --- | --- | --- | --- | --- | --- | --- | --- |
| 1,900 | 2.2981764 | 2.2312034 | 2.3671596 | Global | Ischemic stroke | Deaths | Both | Low physical activity | 1895 to 1904 | 1 |
| 1,905 | 2.1198858 | 2.0720240 | 2.1688532 | Global | Ischemic stroke | Deaths | Both | Low physical activity | 1900 to 1909 | 2 |
| 1,910 | 1.9703687 | 1.9290692 | 2.0125525 | Global | Ischemic stroke | Deaths | Both | Low physical activity | 1905 to 1914 | 3 |
| 1,915 | 1.7991835 | 1.7615144 | 1.8376581 | Global | Ischemic stroke | Deaths | Both | Low physical activity | 1910 to 1919 | 4 |
| 1,920 | 1.6223164 | 1.5887541 | 1.6565878 | Global | Ischemic stroke | Deaths | Both | Low physical activity | 1915 to 1924 | 5 |
| 1,925 | 1.5234375 | 1.4928547 | 1.5546468 | Global | Ischemic stroke | Deaths | Both | Low physical activity | 1920 to 1929 | 6 |
| 1,930 | 1.4094590 | 1.3817250 | 1.4377497 | Global | Ischemic stroke | Deaths | Both | Low physical activity | 1925 to 1934 | 7 |
| 1,935 | 1.2864024 | 1.2611845 | 1.3121246 | Global | Ischemic stroke | Deaths | Both | Low physical activity | 1930 to 1939 | 8 |
| 1,940 | 1.1709607 | 1.1473269 | 1.1950814 | Global | Ischemic stroke | Deaths | Both | Low physical activity | 1935 to 1944 | 9 |
| 1,945 | 1.0552800 | 1.0331193 | 1.0779161 | Global | Ischemic stroke | Deaths | Both | Low physical activity | 1940 to 1949 | 10 |
| 1,950 | 1.0000000 | 1.0000000 | 1.0000000 | Global | Ischemic stroke | Deaths | Both | Low physical activity | 1945 to 1954 | 11 |
| 1,955 | 0.9663664 | 0.9406210 | 0.9928165 | Global | Ischemic stroke | Deaths | Both | Low physical activity | 1950 to 1959 | 12 |
| 1,960 | 0.9392668 | 0.9060929 | 0.9736553 | Global | Ischemic stroke | Deaths | Both | Low physical activity | 1955 to 1964 | 13 |
| 1,965 | 0.9237659 | 0.8832867 | 0.9661002 | Global | Ischemic stroke | Deaths | Both | Low physical activity | 1960 to 1969 | 14 |
| 1,970 | 0.9145867 | 0.8623853 | 0.9699480 | Global | Ischemic stroke | Deaths | Both | Low physical activity | 1965 to 1974 | 15 |
| 1,975 | 0.9525334 | 0.8816659 | 1.0290972 | Global | Ischemic stroke | Deaths | Both | Low physical activity | 1970 to 1979 | 16 |
| 1,980 | 0.9905530 | 0.8961896 | 1.0948523 | Global | Ischemic stroke | Deaths | Both | Low physical activity | 1975 to 1984 | 17 |
| 1,985 | 0.9884909 | 0.8638880 | 1.1310659 | Global | Ischemic stroke | Deaths | Both | Low physical activity | 1980 to 1989 | 18 |
| 1,990 | 0.9527447 | 0.7758000 | 1.1700470 | Global | Ischemic stroke | Deaths | Both | Low physical activity | 1985 to 1994 | 19 |
| 1,900 | 6.2329747 | 5.7310084 | 6.7789070 | World Bank High Income | Ischemic stroke | Deaths | Both | Low physical activity | 1895 to 1904 | 1 |
| 1,905 | 5.8678958 | 5.4256678 | 6.3461684 | World Bank High Income | Ischemic stroke | Deaths | Both | Low physical activity | 1900 to 1909 | 2 |
| 1,910 | 5.3903062 | 4.9904362 | 5.8222166 | World Bank High Income | Ischemic stroke | Deaths | Both | Low physical activity | 1905 to 1914 | 3 |
| 1,915 | 4.7004119 | 4.3506569 | 5.0782842 | World Bank High Income | Ischemic stroke | Deaths | Both | Low physical activity | 1910 to 1919 | 4 |
| 1,920 | 3.8219339 | 3.5376836 | 4.1290235 | World Bank High Income | Ischemic stroke | Deaths | Both | Low physical activity | 1915 to 1924 | 5 |
| 1,925 | 3.1225002 | 2.8926271 | 3.3706410 | World Bank High Income | Ischemic stroke | Deaths | Both | Low physical activity | 1920 to 1929 | 6 |
| 1,930 | 2.4138371 | 2.2366968 | 2.6050064 | World Bank High Income | Ischemic stroke | Deaths | Both | Low physical activity | 1925 to 1934 | 7 |
| 1,935 | 1.8325904 | 1.6971019 | 1.9788957 | World Bank High Income | Ischemic stroke | Deaths | Both | Low physical activity | 1930 to 1939 | 8 |
| 1,940 | 1.4015478 | 1.2935781 | 1.5185292 | World Bank High Income | Ischemic stroke | Deaths | Both | Low physical activity | 1935 to 1944 | 9 |
| 1,945 | 1.1503732 | 1.0571277 | 1.2518435 | World Bank High Income | Ischemic stroke | Deaths | Both | Low physical activity | 1940 to 1949 | 10 |
| 1,950 | 1.0000000 | 1.0000000 | 1.0000000 | World Bank High Income | Ischemic stroke | Deaths | Both | Low physical activity | 1945 to 1954 | 11 |
| 1,955 | 0.9423486 | 0.8455562 | 1.0502209 | World Bank High Income | Ischemic stroke | Deaths | Both | Low physical activity | 1950 to 1959 | 12 |
| 1,960 | 0.8759859 | 0.7632930 | 1.0053169 | World Bank High Income | Ischemic stroke | Deaths | Both | Low physical activity | 1955 to 1964 | 13 |
| 1,965 | 0.8826220 | 0.7459712 | 1.0443053 | World Bank High Income | Ischemic stroke | Deaths | Both | Low physical activity | 1960 to 1969 | 14 |
| 1,970 | 0.9365284 | 0.7585418 | 1.1562783 | World Bank High Income | Ischemic stroke | Deaths | Both | Low physical activity | 1965 to 1974 | 15 |
| 1,975 | 1.1136854 | 0.8537463 | 1.4527678 | World Bank High Income | Ischemic stroke | Deaths | Both | Low physical activity | 1970 to 1979 | 16 |
| 1,980 | 1.2123981 | 0.8588283 | 1.7115285 | World Bank High Income | Ischemic stroke | Deaths | Both | Low physical activity | 1975 to 1984 | 17 |
| 1,985 | 1.1915217 | 0.7235655 | 1.9621222 | World Bank High Income | Ischemic stroke | Deaths | Both | Low physical activity | 1980 to 1989 | 18 |
| 1,990 | 0.9990836 | 0.4268729 | 2.3383262 | World Bank High Income | Ischemic stroke | Deaths | Both | Low physical activity | 1985 to 1994 | 19 |
| 1,900 | 2.0652085 | 1.9384887 | 2.2002120 | World Bank Upper Middle Income | Ischemic stroke | Deaths | Both | Low physical activity | 1895 to 1904 | 1 |
| 1,905 | 1.9594690 | 1.8756625 | 2.0470201 | World Bank Upper Middle Income | Ischemic stroke | Deaths | Both | Low physical activity | 1900 to 1909 | 2 |
| 1,910 | 1.9401875 | 1.8657434 | 2.0176019 | World Bank Upper Middle Income | Ischemic stroke | Deaths | Both | Low physical activity | 1905 to 1914 | 3 |
| 1,915 | 1.8386323 | 1.7688980 | 1.9111158 | World Bank Upper Middle Income | Ischemic stroke | Deaths | Both | Low physical activity | 1910 to 1919 | 4 |
| 1,920 | 1.7566231 | 1.6910982 | 1.8246869 | World Bank Upper Middle Income | Ischemic stroke | Deaths | Both | Low physical activity | 1915 to 1924 | 5 |
| 1,925 | 1.7144062 | 1.6525448 | 1.7785833 | World Bank Upper Middle Income | Ischemic stroke | Deaths | Both | Low physical activity | 1920 to 1929 | 6 |
| 1,930 | 1.5897658 | 1.5337166 | 1.6478632 | World Bank Upper Middle Income | Ischemic stroke | Deaths | Both | Low physical activity | 1925 to 1934 | 7 |
| 1,935 | 1.4197467 | 1.3699582 | 1.4713446 | World Bank Upper Middle Income | Ischemic stroke | Deaths | Both | Low physical activity | 1930 to 1939 | 8 |
| 1,940 | 1.2763614 | 1.2301887 | 1.3242672 | World Bank Upper Middle Income | Ischemic stroke | Deaths | Both | Low physical activity | 1935 to 1944 | 9 |
| 1,945 | 1.0985960 | 1.0569157 | 1.1419201 | World Bank Upper Middle Income | Ischemic stroke | Deaths | Both | Low physical activity | 1940 to 1949 | 10 |
| 1,950 | 1.0000000 | 1.0000000 | 1.0000000 | World Bank Upper Middle Income | Ischemic stroke | Deaths | Both | Low physical activity | 1945 to 1954 | 11 |
| 1,955 | 0.8952183 | 0.8521168 | 0.9404999 | World Bank Upper Middle Income | Ischemic stroke | Deaths | Both | Low physical activity | 1950 to 1959 | 12 |
| 1,960 | 0.8322222 | 0.7788582 | 0.8892426 | World Bank Upper Middle Income | Ischemic stroke | Deaths | Both | Low physical activity | 1955 to 1964 | 13 |
| 1,965 | 0.7496180 | 0.6901440 | 0.8142172 | World Bank Upper Middle Income | Ischemic stroke | Deaths | Both | Low physical activity | 1960 to 1969 | 14 |
| 1,970 | 0.6981128 | 0.6249057 | 0.7798960 | World Bank Upper Middle Income | Ischemic stroke | Deaths | Both | Low physical activity | 1965 to 1974 | 15 |
| 1,975 | 0.6958706 | 0.5975500 | 0.8103688 | World Bank Upper Middle Income | Ischemic stroke | Deaths | Both | Low physical activity | 1970 to 1979 | 16 |
| 1,980 | 0.7024105 | 0.5715170 | 0.8632823 | World Bank Upper Middle Income | Ischemic stroke | Deaths | Both | Low physical activity | 1975 to 1984 | 17 |
| 1,985 | 0.6476397 | 0.4864023 | 0.8623256 | World Bank Upper Middle Income | Ischemic stroke | Deaths | Both | Low physical activity | 1980 to 1989 | 18 |
| 1,990 | 0.5868701 | 0.3698397 | 0.9312591 | World Bank Upper Middle Income | Ischemic stroke | Deaths | Both | Low physical activity | 1985 to 1994 | 19 |
| 1,900 | 1.2884513 | 1.1986671 | 1.3849606 | World Bank Lower Middle Income | Ischemic stroke | Deaths | Both | Low physical activity | 1895 to 1904 | 1 |
| 1,905 | 1.2507814 | 1.1937790 | 1.3105056 | World Bank Lower Middle Income | Ischemic stroke | Deaths | Both | Low physical activity | 1900 to 1909 | 2 |
| 1,910 | 1.2367362 | 1.1885793 | 1.2868441 | World Bank Lower Middle Income | Ischemic stroke | Deaths | Both | Low physical activity | 1905 to 1914 | 3 |
| 1,915 | 1.2153071 | 1.1689213 | 1.2635337 | World Bank Lower Middle Income | Ischemic stroke | Deaths | Both | Low physical activity | 1910 to 1919 | 4 |
| 1,920 | 1.1592485 | 1.1163543 | 1.2037908 | World Bank Lower Middle Income | Ischemic stroke | Deaths | Both | Low physical activity | 1915 to 1924 | 5 |
| 1,925 | 1.1083898 | 1.0691719 | 1.1490462 | World Bank Lower Middle Income | Ischemic stroke | Deaths | Both | Low physical activity | 1920 to 1929 | 6 |
| 1,930 | 1.0670033 | 1.0306111 | 1.1046807 | World Bank Lower Middle Income | Ischemic stroke | Deaths | Both | Low physical activity | 1925 to 1934 | 7 |
| 1,935 | 1.0499993 | 1.0147300 | 1.0864945 | World Bank Lower Middle Income | Ischemic stroke | Deaths | Both | Low physical activity | 1930 to 1939 | 8 |
| 1,940 | 1.0147916 | 0.9801900 | 1.0506147 | World Bank Lower Middle Income | Ischemic stroke | Deaths | Both | Low physical activity | 1935 to 1944 | 9 |
| 1,945 | 0.9985603 | 0.9637841 | 1.0345913 | World Bank Lower Middle Income | Ischemic stroke | Deaths | Both | Low physical activity | 1940 to 1949 | 10 |
| 1,950 | 1.0000000 | 1.0000000 | 1.0000000 | World Bank Lower Middle Income | Ischemic stroke | Deaths | Both | Low physical activity | 1945 to 1954 | 11 |
| 1,955 | 1.0446717 | 0.9988357 | 1.0926111 | World Bank Lower Middle Income | Ischemic stroke | Deaths | Both | Low physical activity | 1950 to 1959 | 12 |
| 1,960 | 1.1114498 | 1.0463073 | 1.1806480 | World Bank Lower Middle Income | Ischemic stroke | Deaths | Both | Low physical activity | 1955 to 1964 | 13 |
| 1,965 | 1.1684411 | 1.0825972 | 1.2610920 | World Bank Lower Middle Income | Ischemic stroke | Deaths | Both | Low physical activity | 1960 to 1969 | 14 |
| 1,970 | 1.1977359 | 1.0834862 | 1.3240329 | World Bank Lower Middle Income | Ischemic stroke | Deaths | Both | Low physical activity | 1965 to 1974 | 15 |
| 1,975 | 1.2781227 | 1.1249689 | 1.4521270 | World Bank Lower Middle Income | Ischemic stroke | Deaths | Both | Low physical activity | 1970 to 1979 | 16 |
| 1,980 | 1.3633444 | 1.1638123 | 1.5970858 | World Bank Lower Middle Income | Ischemic stroke | Deaths | Both | Low physical activity | 1975 to 1984 | 17 |
| 1,985 | 1.4426379 | 1.1757696 | 1.7700780 | World Bank Lower Middle Income | Ischemic stroke | Deaths | Both | Low physical activity | 1980 to 1989 | 18 |
| 1,990 | 1.4640666 | 1.0896689 | 1.9671030 | World Bank Lower Middle Income | Ischemic stroke | Deaths | Both | Low physical activity | 1985 to 1994 | 19 |
| 1,900 | 1.0521034 | 0.8114954 | 1.3640515 | World Bank Low Income | Ischemic stroke | Deaths | Both | Low physical activity | 1895 to 1904 | 1 |
| 1,905 | 1.0275218 | 0.8710814 | 1.2120578 | World Bank Low Income | Ischemic stroke | Deaths | Both | Low physical activity | 1900 to 1909 | 2 |
| 1,910 | 1.0102882 | 0.8842124 | 1.1543405 | World Bank Low Income | Ischemic stroke | Deaths | Both | Low physical activity | 1905 to 1914 | 3 |
| 1,915 | 1.0060368 | 0.8859301 | 1.1424265 | World Bank Low Income | Ischemic stroke | Deaths | Both | Low physical activity | 1910 to 1919 | 4 |
| 1,920 | 1.0301212 | 0.9120315 | 1.1635011 | World Bank Low Income | Ischemic stroke | Deaths | Both | Low physical activity | 1915 to 1924 | 5 |
| 1,925 | 1.0536108 | 0.9378718 | 1.1836327 | World Bank Low Income | Ischemic stroke | Deaths | Both | Low physical activity | 1920 to 1929 | 6 |
| 1,930 | 1.0608242 | 0.9483506 | 1.1866372 | World Bank Low Income | Ischemic stroke | Deaths | Both | Low physical activity | 1925 to 1934 | 7 |
| 1,935 | 1.0677482 | 0.9567436 | 1.1916320 | World Bank Low Income | Ischemic stroke | Deaths | Both | Low physical activity | 1930 to 1939 | 8 |
| 1,940 | 1.0666529 | 0.9548261 | 1.1915765 | World Bank Low Income | Ischemic stroke | Deaths | Both | Low physical activity | 1935 to 1944 | 9 |
| 1,945 | 1.0431906 | 0.9321108 | 1.1675078 | World Bank Low Income | Ischemic stroke | Deaths | Both | Low physical activity | 1940 to 1949 | 10 |
| 1,950 | 1.0000000 | 1.0000000 | 1.0000000 | World Bank Low Income | Ischemic stroke | Deaths | Both | Low physical activity | 1945 to 1954 | 11 |
| 1,955 | 0.9786363 | 0.8480867 | 1.1292820 | World Bank Low Income | Ischemic stroke | Deaths | Both | Low physical activity | 1950 to 1959 | 12 |
| 1,960 | 0.9995993 | 0.8299511 | 1.2039249 | World Bank Low Income | Ischemic stroke | Deaths | Both | Low physical activity | 1955 to 1964 | 13 |
| 1,965 | 1.1816609 | 0.9513167 | 1.4677787 | World Bank Low Income | Ischemic stroke | Deaths | Both | Low physical activity | 1960 to 1969 | 14 |
| 1,970 | 1.3875870 | 1.0682948 | 1.8023094 | World Bank Low Income | Ischemic stroke | Deaths | Both | Low physical activity | 1965 to 1974 | 15 |
| 1,975 | 1.2279915 | 0.8797000 | 1.7141787 | World Bank Low Income | Ischemic stroke | Deaths | Both | Low physical activity | 1970 to 1979 | 16 |
| 1,980 | 1.1734232 | 0.7641094 | 1.8019960 | World Bank Low Income | Ischemic stroke | Deaths | Both | Low physical activity | 1975 to 1984 | 17 |
| 1,985 | 1.2436135 | 0.7225840 | 2.1403387 | World Bank Low Income | Ischemic stroke | Deaths | Both | Low physical activity | 1980 to 1989 | 18 |
| 1,990 | 1.3696720 | 0.6376685 | 2.9419697 | World Bank Low Income | Ischemic stroke | Deaths | Both | Low physical activity | 1985 to 1994 | 19 |
| 1,900 | 2.0371190 | 1.9306168 | 2.1494963 | Global | Ischemic stroke | Deaths | Male | Low physical activity | 1895 to 1904 | 1 |
| 1,905 | 1.8448582 | 1.7812614 | 1.9107256 | Global | Ischemic stroke | Deaths | Male | Low physical activity | 1900 to 1909 | 2 |
| 1,910 | 1.7629970 | 1.7104592 | 1.8171485 | Global | Ischemic stroke | Deaths | Male | Low physical activity | 1905 to 1914 | 3 |
| 1,915 | 1.6574824 | 1.6088656 | 1.7075682 | Global | Ischemic stroke | Deaths | Male | Low physical activity | 1910 to 1919 | 4 |
| 1,920 | 1.5277863 | 1.4840481 | 1.5728135 | Global | Ischemic stroke | Deaths | Male | Low physical activity | 1915 to 1924 | 5 |
| 1,925 | 1.4474170 | 1.4076844 | 1.4882710 | Global | Ischemic stroke | Deaths | Male | Low physical activity | 1920 to 1929 | 6 |
| 1,930 | 1.3667951 | 1.3304450 | 1.4041384 | Global | Ischemic stroke | Deaths | Male | Low physical activity | 1925 to 1934 | 7 |
| 1,935 | 1.2594988 | 1.2262458 | 1.2936534 | Global | Ischemic stroke | Deaths | Male | Low physical activity | 1930 to 1939 | 8 |
| 1,940 | 1.1595873 | 1.1281612 | 1.1918888 | Global | Ischemic stroke | Deaths | Male | Low physical activity | 1935 to 1944 | 9 |
| 1,945 | 1.0466906 | 1.0173508 | 1.0768765 | Global | Ischemic stroke | Deaths | Male | Low physical activity | 1940 to 1949 | 10 |
| 1,950 | 1.0000000 | 1.0000000 | 1.0000000 | Global | Ischemic stroke | Deaths | Male | Low physical activity | 1945 to 1954 | 11 |
| 1,955 | 0.9829693 | 0.9486387 | 1.0185422 | Global | Ischemic stroke | Deaths | Male | Low physical activity | 1950 to 1959 | 12 |
| 1,960 | 0.9670718 | 0.9224617 | 1.0138392 | Global | Ischemic stroke | Deaths | Male | Low physical activity | 1955 to 1964 | 13 |
| 1,965 | 0.9468827 | 0.8921820 | 1.0049371 | Global | Ischemic stroke | Deaths | Male | Low physical activity | 1960 to 1969 | 14 |
| 1,970 | 0.9396220 | 0.8676981 | 1.0175077 | Global | Ischemic stroke | Deaths | Male | Low physical activity | 1965 to 1974 | 15 |
| 1,975 | 0.9928749 | 0.8909118 | 1.1065074 | Global | Ischemic stroke | Deaths | Male | Low physical activity | 1970 to 1979 | 16 |
| 1,980 | 1.0645865 | 0.9235231 | 1.2271967 | Global | Ischemic stroke | Deaths | Male | Low physical activity | 1975 to 1984 | 17 |
| 1,985 | 1.1013617 | 0.9086924 | 1.3348826 | Global | Ischemic stroke | Deaths | Male | Low physical activity | 1980 to 1989 | 18 |
| 1,990 | 1.1079881 | 0.8277967 | 1.4830184 | Global | Ischemic stroke | Deaths | Male | Low physical activity | 1985 to 1994 | 19 |
| 1,900 | 6.1300977 | 5.5066843 | 6.8240878 | World Bank High Income | Ischemic stroke | Deaths | Male | Low physical activity | 1895 to 1904 | 1 |
| 1,905 | 5.6438030 | 5.1617387 | 6.1708883 | World Bank High Income | Ischemic stroke | Deaths | Male | Low physical activity | 1900 to 1909 | 2 |
| 1,910 | 5.1301927 | 4.7124254 | 5.5849961 | World Bank High Income | Ischemic stroke | Deaths | Male | Low physical activity | 1905 to 1914 | 3 |
| 1,915 | 4.4272518 | 4.0667525 | 4.8197077 | World Bank High Income | Ischemic stroke | Deaths | Male | Low physical activity | 1910 to 1919 | 4 |
| 1,920 | 3.5781775 | 3.2878685 | 3.8941199 | World Bank High Income | Ischemic stroke | Deaths | Male | Low physical activity | 1915 to 1924 | 5 |
| 1,925 | 2.9219227 | 2.6888638 | 3.1751820 | World Bank High Income | Ischemic stroke | Deaths | Male | Low physical activity | 1920 to 1929 | 6 |
| 1,930 | 2.3158279 | 2.1326821 | 2.5147015 | World Bank High Income | Ischemic stroke | Deaths | Male | Low physical activity | 1925 to 1934 | 7 |
| 1,935 | 1.7850690 | 1.6429830 | 1.9394426 | World Bank High Income | Ischemic stroke | Deaths | Male | Low physical activity | 1930 to 1939 | 8 |
| 1,940 | 1.3916368 | 1.2764399 | 1.5172300 | World Bank High Income | Ischemic stroke | Deaths | Male | Low physical activity | 1935 to 1944 | 9 |
| 1,945 | 1.1409603 | 1.0421363 | 1.2491556 | World Bank High Income | Ischemic stroke | Deaths | Male | Low physical activity | 1940 to 1949 | 10 |
| 1,950 | 1.0000000 | 1.0000000 | 1.0000000 | World Bank High Income | Ischemic stroke | Deaths | Male | Low physical activity | 1945 to 1954 | 11 |
| 1,955 | 0.9437433 | 0.8419341 | 1.0578635 | World Bank High Income | Ischemic stroke | Deaths | Male | Low physical activity | 1950 to 1959 | 12 |
| 1,960 | 0.8673387 | 0.7499919 | 1.0030459 | World Bank High Income | Ischemic stroke | Deaths | Male | Low physical activity | 1955 to 1964 | 13 |
| 1,965 | 0.8783178 | 0.7338163 | 1.0512743 | World Bank High Income | Ischemic stroke | Deaths | Male | Low physical activity | 1960 to 1969 | 14 |
| 1,970 | 0.9338253 | 0.7412059 | 1.1765013 | World Bank High Income | Ischemic stroke | Deaths | Male | Low physical activity | 1965 to 1974 | 15 |
| 1,975 | 1.1259793 | 0.8308266 | 1.5259855 | World Bank High Income | Ischemic stroke | Deaths | Male | Low physical activity | 1970 to 1979 | 16 |
| 1,980 | 1.2379842 | 0.8258222 | 1.8558533 | World Bank High Income | Ischemic stroke | Deaths | Male | Low physical activity | 1975 to 1984 | 17 |
| 1,985 | 1.3703442 | 0.7603949 | 2.4695629 | World Bank High Income | Ischemic stroke | Deaths | Male | Low physical activity | 1980 to 1989 | 18 |
| 1,990 | 0.9541456 | 0.3181499 | 2.8615246 | World Bank High Income | Ischemic stroke | Deaths | Male | Low physical activity | 1985 to 1994 | 19 |
| 1,900 | 1.7105073 | 1.5632410 | 1.8716471 | World Bank Upper Middle Income | Ischemic stroke | Deaths | Male | Low physical activity | 1895 to 1904 | 1 |
| 1,905 | 1.5781502 | 1.4974025 | 1.6632522 | World Bank Upper Middle Income | Ischemic stroke | Deaths | Male | Low physical activity | 1900 to 1909 | 2 |
| 1,910 | 1.6480237 | 1.5781881 | 1.7209495 | World Bank Upper Middle Income | Ischemic stroke | Deaths | Male | Low physical activity | 1905 to 1914 | 3 |
| 1,915 | 1.6223954 | 1.5557338 | 1.6919134 | World Bank Upper Middle Income | Ischemic stroke | Deaths | Male | Low physical activity | 1910 to 1919 | 4 |
| 1,920 | 1.5857364 | 1.5225813 | 1.6515111 | World Bank Upper Middle Income | Ischemic stroke | Deaths | Male | Low physical activity | 1915 to 1924 | 5 |
| 1,925 | 1.5680537 | 1.5084099 | 1.6300558 | World Bank Upper Middle Income | Ischemic stroke | Deaths | Male | Low physical activity | 1920 to 1929 | 6 |
| 1,930 | 1.4794787 | 1.4251673 | 1.5358598 | World Bank Upper Middle Income | Ischemic stroke | Deaths | Male | Low physical activity | 1925 to 1934 | 7 |
| 1,935 | 1.3434412 | 1.2945983 | 1.3941269 | World Bank Upper Middle Income | Ischemic stroke | Deaths | Male | Low physical activity | 1930 to 1939 | 8 |
| 1,940 | 1.2392297 | 1.1929293 | 1.2873271 | World Bank Upper Middle Income | Ischemic stroke | Deaths | Male | Low physical activity | 1935 to 1944 | 9 |
| 1,945 | 1.0819499 | 1.0398423 | 1.1257627 | World Bank Upper Middle Income | Ischemic stroke | Deaths | Male | Low physical activity | 1940 to 1949 | 10 |
| 1,950 | 1.0000000 | 1.0000000 | 1.0000000 | World Bank Upper Middle Income | Ischemic stroke | Deaths | Male | Low physical activity | 1945 to 1954 | 11 |
| 1,955 | 0.9239732 | 0.8792109 | 0.9710145 | World Bank Upper Middle Income | Ischemic stroke | Deaths | Male | Low physical activity | 1950 to 1959 | 12 |
| 1,960 | 0.8846598 | 0.8279287 | 0.9452783 | World Bank Upper Middle Income | Ischemic stroke | Deaths | Male | Low physical activity | 1955 to 1964 | 13 |
| 1,965 | 0.8068821 | 0.7425806 | 0.8767516 | World Bank Upper Middle Income | Ischemic stroke | Deaths | Male | Low physical activity | 1960 to 1969 | 14 |
| 1,970 | 0.7616425 | 0.6805608 | 0.8523843 | World Bank Upper Middle Income | Ischemic stroke | Deaths | Male | Low physical activity | 1965 to 1974 | 15 |
| 1,975 | 0.7852651 | 0.6699337 | 0.9204512 | World Bank Upper Middle Income | Ischemic stroke | Deaths | Male | Low physical activity | 1970 to 1979 | 16 |
| 1,980 | 0.8221207 | 0.6601178 | 1.0238814 | World Bank Upper Middle Income | Ischemic stroke | Deaths | Male | Low physical activity | 1975 to 1984 | 17 |
| 1,985 | 0.7961830 | 0.5844230 | 1.0846721 | World Bank Upper Middle Income | Ischemic stroke | Deaths | Male | Low physical activity | 1980 to 1989 | 18 |
| 1,990 | 0.7609493 | 0.4580451 | 1.2641635 | World Bank Upper Middle Income | Ischemic stroke | Deaths | Male | Low physical activity | 1985 to 1994 | 19 |
| 1,900 | 1.2975175 | 1.1585130 | 1.4532006 | World Bank Lower Middle Income | Ischemic stroke | Deaths | Male | Low physical activity | 1895 to 1904 | 1 |
| 1,905 | 1.2365642 | 1.1546131 | 1.3243319 | World Bank Lower Middle Income | Ischemic stroke | Deaths | Male | Low physical activity | 1900 to 1909 | 2 |
| 1,910 | 1.2154214 | 1.1492300 | 1.2854251 | World Bank Lower Middle Income | Ischemic stroke | Deaths | Male | Low physical activity | 1905 to 1914 | 3 |
| 1,915 | 1.1999206 | 1.1366074 | 1.2667606 | World Bank Lower Middle Income | Ischemic stroke | Deaths | Male | Low physical activity | 1910 to 1919 | 4 |
| 1,920 | 1.1564307 | 1.0979505 | 1.2180258 | World Bank Lower Middle Income | Ischemic stroke | Deaths | Male | Low physical activity | 1915 to 1924 | 5 |
| 1,925 | 1.1132917 | 1.0600153 | 1.1692458 | World Bank Lower Middle Income | Ischemic stroke | Deaths | Male | Low physical activity | 1920 to 1929 | 6 |
| 1,930 | 1.0793915 | 1.0299615 | 1.1311936 | World Bank Lower Middle Income | Ischemic stroke | Deaths | Male | Low physical activity | 1925 to 1934 | 7 |
| 1,935 | 1.0615194 | 1.0136062 | 1.1116975 | World Bank Lower Middle Income | Ischemic stroke | Deaths | Male | Low physical activity | 1930 to 1939 | 8 |
| 1,940 | 1.0290960 | 0.9819530 | 1.0785024 | World Bank Lower Middle Income | Ischemic stroke | Deaths | Male | Low physical activity | 1935 to 1944 | 9 |
| 1,945 | 0.9967418 | 0.9502995 | 1.0454537 | World Bank Lower Middle Income | Ischemic stroke | Deaths | Male | Low physical activity | 1940 to 1949 | 10 |
| 1,950 | 1.0000000 | 1.0000000 | 1.0000000 | World Bank Lower Middle Income | Ischemic stroke | Deaths | Male | Low physical activity | 1945 to 1954 | 11 |
| 1,955 | 1.0522891 | 0.9915475 | 1.1167518 | World Bank Lower Middle Income | Ischemic stroke | Deaths | Male | Low physical activity | 1950 to 1959 | 12 |
| 1,960 | 1.1239264 | 1.0376384 | 1.2173899 | World Bank Lower Middle Income | Ischemic stroke | Deaths | Male | Low physical activity | 1955 to 1964 | 13 |
| 1,965 | 1.1569061 | 1.0440408 | 1.2819725 | World Bank Lower Middle Income | Ischemic stroke | Deaths | Male | Low physical activity | 1960 to 1969 | 14 |
| 1,970 | 1.1895731 | 1.0360672 | 1.3658228 | World Bank Lower Middle Income | Ischemic stroke | Deaths | Male | Low physical activity | 1965 to 1974 | 15 |
| 1,975 | 1.2709863 | 1.0595628 | 1.5245970 | World Bank Lower Middle Income | Ischemic stroke | Deaths | Male | Low physical activity | 1970 to 1979 | 16 |
| 1,980 | 1.3686650 | 1.0924127 | 1.7147768 | World Bank Lower Middle Income | Ischemic stroke | Deaths | Male | Low physical activity | 1975 to 1984 | 17 |
| 1,985 | 1.4942527 | 1.1195927 | 1.9942889 | World Bank Lower Middle Income | Ischemic stroke | Deaths | Male | Low physical activity | 1980 to 1989 | 18 |
| 1,990 | 1.5492638 | 1.0286869 | 2.3332835 | World Bank Lower Middle Income | Ischemic stroke | Deaths | Male | Low physical activity | 1985 to 1994 | 19 |
| 1,900 | 1.0158793 | 0.6413734 | 1.6090638 | World Bank Low Income | Ischemic stroke | Deaths | Male | Low physical activity | 1895 to 1904 | 1 |
| 1,905 | 1.0481820 | 0.8069002 | 1.3616125 | World Bank Low Income | Ischemic stroke | Deaths | Male | Low physical activity | 1900 to 1909 | 2 |
| 1,910 | 1.0315803 | 0.8436986 | 1.2613009 | World Bank Low Income | Ischemic stroke | Deaths | Male | Low physical activity | 1905 to 1914 | 3 |
| 1,915 | 1.0108981 | 0.8365164 | 1.2216319 | World Bank Low Income | Ischemic stroke | Deaths | Male | Low physical activity | 1910 to 1919 | 4 |
| 1,920 | 1.0266100 | 0.8578006 | 1.2286400 | World Bank Low Income | Ischemic stroke | Deaths | Male | Low physical activity | 1915 to 1924 | 5 |
| 1,925 | 1.0481363 | 0.8841139 | 1.2425884 | World Bank Low Income | Ischemic stroke | Deaths | Male | Low physical activity | 1920 to 1929 | 6 |
| 1,930 | 1.0458366 | 0.8886905 | 1.2307707 | World Bank Low Income | Ischemic stroke | Deaths | Male | Low physical activity | 1925 to 1934 | 7 |
| 1,935 | 1.0656079 | 0.9088354 | 1.2494236 | World Bank Low Income | Ischemic stroke | Deaths | Male | Low physical activity | 1930 to 1939 | 8 |
| 1,940 | 1.0830218 | 0.9227193 | 1.2711735 | World Bank Low Income | Ischemic stroke | Deaths | Male | Low physical activity | 1935 to 1944 | 9 |
| 1,945 | 1.0584440 | 0.9000480 | 1.2447155 | World Bank Low Income | Ischemic stroke | Deaths | Male | Low physical activity | 1940 to 1949 | 10 |
| 1,950 | 1.0000000 | 1.0000000 | 1.0000000 | World Bank Low Income | Ischemic stroke | Deaths | Male | Low physical activity | 1945 to 1954 | 11 |
| 1,955 | 0.9845861 | 0.8022063 | 1.2084296 | World Bank Low Income | Ischemic stroke | Deaths | Male | Low physical activity | 1950 to 1959 | 12 |
| 1,960 | 1.0286446 | 0.7845807 | 1.3486309 | World Bank Low Income | Ischemic stroke | Deaths | Male | Low physical activity | 1955 to 1964 | 13 |
| 1,965 | 1.1809891 | 0.8538317 | 1.6335013 | World Bank Low Income | Ischemic stroke | Deaths | Male | Low physical activity | 1960 to 1969 | 14 |
| 1,970 | 1.3622979 | 0.9088743 | 2.0419277 | World Bank Low Income | Ischemic stroke | Deaths | Male | Low physical activity | 1965 to 1974 | 15 |
| 1,975 | 1.3629440 | 0.8043982 | 2.3093244 | World Bank Low Income | Ischemic stroke | Deaths | Male | Low physical activity | 1970 to 1979 | 16 |
| 1,980 | 1.2325547 | 0.6126684 | 2.4796301 | World Bank Low Income | Ischemic stroke | Deaths | Male | Low physical activity | 1975 to 1984 | 17 |
| 1,985 | 1.4449119 | 0.6197707 | 3.3686177 | World Bank Low Income | Ischemic stroke | Deaths | Male | Low physical activity | 1980 to 1989 | 18 |
| 1,990 | 1.6077270 | 0.5278406 | 4.8969070 | World Bank Low Income | Ischemic stroke | Deaths | Male | Low physical activity | 1985 to 1994 | 19 |
| 1,900 | 2.4808969 | 2.3868128 | 2.5786897 | Global | Ischemic stroke | Deaths | Female | Low physical activity | 1895 to 1904 | 1 |
| 1,905 | 2.3101903 | 2.2366583 | 2.3861398 | Global | Ischemic stroke | Deaths | Female | Low physical activity | 1900 to 1909 | 2 |
| 1,910 | 2.1301152 | 2.0653033 | 2.1969611 | Global | Ischemic stroke | Deaths | Female | Low physical activity | 1905 to 1914 | 3 |
| 1,915 | 1.9282975 | 1.8694619 | 1.9889847 | Global | Ischemic stroke | Deaths | Female | Low physical activity | 1910 to 1919 | 4 |
| 1,920 | 1.7262111 | 1.6738878 | 1.7801700 | Global | Ischemic stroke | Deaths | Female | Low physical activity | 1915 to 1924 | 5 |
| 1,925 | 1.6170724 | 1.5691524 | 1.6664559 | Global | Ischemic stroke | Deaths | Female | Low physical activity | 1920 to 1929 | 6 |
| 1,930 | 1.4757688 | 1.4326149 | 1.5202225 | Global | Ischemic stroke | Deaths | Female | Low physical activity | 1925 to 1934 | 7 |
| 1,935 | 1.3327565 | 1.2938553 | 1.3728274 | Global | Ischemic stroke | Deaths | Female | Low physical activity | 1930 to 1939 | 8 |
| 1,940 | 1.1950143 | 1.1590740 | 1.2320690 | Global | Ischemic stroke | Deaths | Female | Low physical activity | 1935 to 1944 | 9 |
| 1,945 | 1.0683483 | 1.0347692 | 1.1030172 | Global | Ischemic stroke | Deaths | Female | Low physical activity | 1940 to 1949 | 10 |
| 1,950 | 1.0000000 | 1.0000000 | 1.0000000 | Global | Ischemic stroke | Deaths | Female | Low physical activity | 1945 to 1954 | 11 |
| 1,955 | 0.9445184 | 0.9060866 | 0.9845802 | Global | Ischemic stroke | Deaths | Female | Low physical activity | 1950 to 1959 | 12 |
| 1,960 | 0.9006370 | 0.8520018 | 0.9520484 | Global | Ischemic stroke | Deaths | Female | Low physical activity | 1955 to 1964 | 13 |
| 1,965 | 0.8916342 | 0.8329111 | 0.9544975 | Global | Ischemic stroke | Deaths | Female | Low physical activity | 1960 to 1969 | 14 |
| 1,970 | 0.8788120 | 0.8054132 | 0.9588998 | Global | Ischemic stroke | Deaths | Female | Low physical activity | 1965 to 1974 | 15 |
| 1,975 | 0.9006089 | 0.8062826 | 1.0059704 | Global | Ischemic stroke | Deaths | Female | Low physical activity | 1970 to 1979 | 16 |
| 1,980 | 0.9123072 | 0.7920596 | 1.0508104 | Global | Ischemic stroke | Deaths | Female | Low physical activity | 1975 to 1984 | 17 |
| 1,985 | 0.8763461 | 0.7251162 | 1.0591164 | Global | Ischemic stroke | Deaths | Female | Low physical activity | 1980 to 1989 | 18 |
| 1,990 | 0.8061358 | 0.6030268 | 1.0776551 | Global | Ischemic stroke | Deaths | Female | Low physical activity | 1985 to 1994 | 19 |
| 1,900 | 6.4616645 | 5.8266538 | 7.1658811 | World Bank High Income | Ischemic stroke | Deaths | Female | Low physical activity | 1895 to 1904 | 1 |
| 1,905 | 6.1477250 | 5.5640455 | 6.7926335 | World Bank High Income | Ischemic stroke | Deaths | Female | Low physical activity | 1900 to 1909 | 2 |
| 1,910 | 5.6984089 | 5.1614250 | 6.2912594 | World Bank High Income | Ischemic stroke | Deaths | Female | Low physical activity | 1905 to 1914 | 3 |
| 1,915 | 5.0226000 | 4.5480750 | 5.5466347 | World Bank High Income | Ischemic stroke | Deaths | Female | Low physical activity | 1910 to 1919 | 4 |
| 1,920 | 4.1104809 | 3.7220770 | 4.5394153 | World Bank High Income | Ischemic stroke | Deaths | Female | Low physical activity | 1915 to 1924 | 5 |
| 1,925 | 3.3743115 | 3.0575597 | 3.7238776 | World Bank High Income | Ischemic stroke | Deaths | Female | Low physical activity | 1920 to 1929 | 6 |
| 1,930 | 2.5917976 | 2.3488525 | 2.8598709 | World Bank High Income | Ischemic stroke | Deaths | Female | Low physical activity | 1925 to 1934 | 7 |
| 1,935 | 1.9426346 | 1.7593553 | 2.1450068 | World Bank High Income | Ischemic stroke | Deaths | Female | Low physical activity | 1930 to 1939 | 8 |
| 1,940 | 1.4400894 | 1.2988681 | 1.5966651 | World Bank High Income | Ischemic stroke | Deaths | Female | Low physical activity | 1935 to 1944 | 9 |
| 1,945 | 1.1670217 | 1.0461697 | 1.3018343 | World Bank High Income | Ischemic stroke | Deaths | Female | Low physical activity | 1940 to 1949 | 10 |
| 1,950 | 1.0000000 | 1.0000000 | 1.0000000 | World Bank High Income | Ischemic stroke | Deaths | Female | Low physical activity | 1945 to 1954 | 11 |
| 1,955 | 0.9475704 | 0.8210515 | 1.0935851 | World Bank High Income | Ischemic stroke | Deaths | Female | Low physical activity | 1950 to 1959 | 12 |
| 1,960 | 0.9033549 | 0.7536677 | 1.0827718 | World Bank High Income | Ischemic stroke | Deaths | Female | Low physical activity | 1955 to 1964 | 13 |
| 1,965 | 0.9061081 | 0.7290676 | 1.1261396 | World Bank High Income | Ischemic stroke | Deaths | Female | Low physical activity | 1960 to 1969 | 14 |
| 1,970 | 0.9491892 | 0.7298049 | 1.2345218 | World Bank High Income | Ischemic stroke | Deaths | Female | Low physical activity | 1965 to 1974 | 15 |
| 1,975 | 1.0962168 | 0.7982645 | 1.5053800 | World Bank High Income | Ischemic stroke | Deaths | Female | Low physical activity | 1970 to 1979 | 16 |
| 1,980 | 1.1746391 | 0.7874102 | 1.7522975 | World Bank High Income | Ischemic stroke | Deaths | Female | Low physical activity | 1975 to 1984 | 17 |
| 1,985 | 1.1116669 | 0.6323551 | 1.9542869 | World Bank High Income | Ischemic stroke | Deaths | Female | Low physical activity | 1980 to 1989 | 18 |
| 1,990 | 1.0274231 | 0.4126370 | 2.5581763 | World Bank High Income | Ischemic stroke | Deaths | Female | Low physical activity | 1985 to 1994 | 19 |
| 1,900 | 2.3983616 | 2.2030839 | 2.6109484 | World Bank Upper Middle Income | Ischemic stroke | Deaths | Female | Low physical activity | 1895 to 1904 | 1 |
| 1,905 | 2.2989333 | 2.1553020 | 2.4521362 | World Bank Upper Middle Income | Ischemic stroke | Deaths | Female | Low physical activity | 1900 to 1909 | 2 |
| 1,910 | 2.2234234 | 2.0939101 | 2.3609474 | World Bank Upper Middle Income | Ischemic stroke | Deaths | Female | Low physical activity | 1905 to 1914 | 3 |
| 1,915 | 2.0705481 | 1.9504910 | 2.1979949 | World Bank Upper Middle Income | Ischemic stroke | Deaths | Female | Low physical activity | 1910 to 1919 | 4 |
| 1,920 | 1.9617503 | 1.8491095 | 2.0812526 | World Bank Upper Middle Income | Ischemic stroke | Deaths | Female | Low physical activity | 1915 to 1924 | 5 |
| 1,925 | 1.9065048 | 1.7997111 | 2.0196356 | World Bank Upper Middle Income | Ischemic stroke | Deaths | Female | Low physical activity | 1920 to 1929 | 6 |
| 1,930 | 1.7477174 | 1.6513372 | 1.8497229 | World Bank Upper Middle Income | Ischemic stroke | Deaths | Female | Low physical activity | 1925 to 1934 | 7 |
| 1,935 | 1.5374796 | 1.4529279 | 1.6269516 | World Bank Upper Middle Income | Ischemic stroke | Deaths | Female | Low physical activity | 1930 to 1939 | 8 |
| 1,940 | 1.3397749 | 1.2636699 | 1.4204633 | World Bank Upper Middle Income | Ischemic stroke | Deaths | Female | Low physical activity | 1935 to 1944 | 9 |
| 1,945 | 1.1248641 | 1.0574873 | 1.1965337 | World Bank Upper Middle Income | Ischemic stroke | Deaths | Female | Low physical activity | 1940 to 1949 | 10 |
| 1,950 | 1.0000000 | 1.0000000 | 1.0000000 | World Bank Upper Middle Income | Ischemic stroke | Deaths | Female | Low physical activity | 1945 to 1954 | 11 |
| 1,955 | 0.8548392 | 0.7884293 | 0.9268430 | World Bank Upper Middle Income | Ischemic stroke | Deaths | Female | Low physical activity | 1950 to 1959 | 12 |
| 1,960 | 0.7578549 | 0.6791220 | 0.8457157 | World Bank Upper Middle Income | Ischemic stroke | Deaths | Female | Low physical activity | 1955 to 1964 | 13 |
| 1,965 | 0.6683740 | 0.5834406 | 0.7656714 | World Bank Upper Middle Income | Ischemic stroke | Deaths | Female | Low physical activity | 1960 to 1969 | 14 |
| 1,970 | 0.6081388 | 0.5082932 | 0.7275974 | World Bank Upper Middle Income | Ischemic stroke | Deaths | Female | Low physical activity | 1965 to 1974 | 15 |
| 1,975 | 0.5843249 | 0.4602188 | 0.7418984 | World Bank Upper Middle Income | Ischemic stroke | Deaths | Female | Low physical activity | 1970 to 1979 | 16 |
| 1,980 | 0.5618791 | 0.4091763 | 0.7715698 | World Bank Upper Middle Income | Ischemic stroke | Deaths | Female | Low physical activity | 1975 to 1984 | 17 |
| 1,985 | 0.4990117 | 0.3239162 | 0.7687565 | World Bank Upper Middle Income | Ischemic stroke | Deaths | Female | Low physical activity | 1980 to 1989 | 18 |
| 1,990 | 0.4301618 | 0.2169176 | 0.8530390 | World Bank Upper Middle Income | Ischemic stroke | Deaths | Female | Low physical activity | 1985 to 1994 | 19 |
| 1,900 | 1.2719806 | 1.1560947 | 1.3994828 | World Bank Lower Middle Income | Ischemic stroke | Deaths | Female | Low physical activity | 1895 to 1904 | 1 |
| 1,905 | 1.2526125 | 1.1734387 | 1.3371281 | World Bank Lower Middle Income | Ischemic stroke | Deaths | Female | Low physical activity | 1900 to 1909 | 2 |
| 1,910 | 1.2428681 | 1.1734554 | 1.3163867 | World Bank Lower Middle Income | Ischemic stroke | Deaths | Female | Low physical activity | 1905 to 1914 | 3 |
| 1,915 | 1.2176234 | 1.1504126 | 1.2887608 | World Bank Lower Middle Income | Ischemic stroke | Deaths | Female | Low physical activity | 1910 to 1919 | 4 |
| 1,920 | 1.1531390 | 1.0908903 | 1.2189399 | World Bank Lower Middle Income | Ischemic stroke | Deaths | Female | Low physical activity | 1915 to 1924 | 5 |
| 1,925 | 1.0974875 | 1.0403384 | 1.1577760 | World Bank Lower Middle Income | Ischemic stroke | Deaths | Female | Low physical activity | 1920 to 1929 | 6 |
| 1,930 | 1.0501169 | 0.9970538 | 1.1060040 | World Bank Lower Middle Income | Ischemic stroke | Deaths | Female | Low physical activity | 1925 to 1934 | 7 |
| 1,935 | 1.0342625 | 0.9828119 | 1.0884066 | World Bank Lower Middle Income | Ischemic stroke | Deaths | Female | Low physical activity | 1930 to 1939 | 8 |
| 1,940 | 0.9980420 | 0.9477017 | 1.0510564 | World Bank Lower Middle Income | Ischemic stroke | Deaths | Female | Low physical activity | 1935 to 1944 | 9 |
| 1,945 | 0.9969446 | 0.9454539 | 1.0512397 | World Bank Lower Middle Income | Ischemic stroke | Deaths | Female | Low physical activity | 1940 to 1949 | 10 |
| 1,950 | 1.0000000 | 1.0000000 | 1.0000000 | World Bank Lower Middle Income | Ischemic stroke | Deaths | Female | Low physical activity | 1945 to 1954 | 11 |
| 1,955 | 1.0383987 | 0.9697433 | 1.1119147 | World Bank Lower Middle Income | Ischemic stroke | Deaths | Female | Low physical activity | 1950 to 1959 | 12 |
| 1,960 | 1.0995655 | 1.0025758 | 1.2059381 | World Bank Lower Middle Income | Ischemic stroke | Deaths | Female | Low physical activity | 1955 to 1964 | 13 |
| 1,965 | 1.1879941 | 1.0597824 | 1.3317168 | World Bank Lower Middle Income | Ischemic stroke | Deaths | Female | Low physical activity | 1960 to 1969 | 14 |
| 1,970 | 1.2067332 | 1.0428040 | 1.3964320 | World Bank Lower Middle Income | Ischemic stroke | Deaths | Female | Low physical activity | 1965 to 1974 | 15 |
| 1,975 | 1.2876396 | 1.0758851 | 1.5410713 | World Bank Lower Middle Income | Ischemic stroke | Deaths | Female | Low physical activity | 1970 to 1979 | 16 |
| 1,980 | 1.3388893 | 1.0705783 | 1.6744451 | World Bank Lower Middle Income | Ischemic stroke | Deaths | Female | Low physical activity | 1975 to 1984 | 17 |
| 1,985 | 1.3944593 | 1.0430955 | 1.8641792 | World Bank Lower Middle Income | Ischemic stroke | Deaths | Female | Low physical activity | 1980 to 1989 | 18 |
| 1,990 | 1.3632129 | 0.8898750 | 2.0883265 | World Bank Lower Middle Income | Ischemic stroke | Deaths | Female | Low physical activity | 1985 to 1994 | 19 |
| 1,900 | 1.0551216 | 0.7650383 | 1.4551971 | World Bank Low Income | Ischemic stroke | Deaths | Female | Low physical activity | 1895 to 1904 | 1 |
| 1,905 | 1.0278540 | 0.8280043 | 1.2759400 | World Bank Low Income | Ischemic stroke | Deaths | Female | Low physical activity | 1900 to 1909 | 2 |
| 1,910 | 1.0111602 | 0.8445745 | 1.2106036 | World Bank Low Income | Ischemic stroke | Deaths | Female | Low physical activity | 1905 to 1914 | 3 |
| 1,915 | 1.0175865 | 0.8558288 | 1.2099177 | World Bank Low Income | Ischemic stroke | Deaths | Female | Low physical activity | 1910 to 1919 | 4 |
| 1,920 | 1.0444193 | 0.8839637 | 1.2340004 | World Bank Low Income | Ischemic stroke | Deaths | Female | Low physical activity | 1915 to 1924 | 5 |
| 1,925 | 1.0638271 | 0.9061912 | 1.2488844 | World Bank Low Income | Ischemic stroke | Deaths | Female | Low physical activity | 1920 to 1929 | 6 |
| 1,930 | 1.0721789 | 0.9180569 | 1.2521746 | World Bank Low Income | Ischemic stroke | Deaths | Female | Low physical activity | 1925 to 1934 | 7 |
| 1,935 | 1.0692072 | 0.9182383 | 1.2449971 | World Bank Low Income | Ischemic stroke | Deaths | Female | Low physical activity | 1930 to 1939 | 8 |
| 1,940 | 1.0516340 | 0.9017343 | 1.2264522 | World Bank Low Income | Ischemic stroke | Deaths | Female | Low physical activity | 1935 to 1944 | 9 |
| 1,945 | 1.0303888 | 0.8810785 | 1.2050016 | World Bank Low Income | Ischemic stroke | Deaths | Female | Low physical activity | 1940 to 1949 | 10 |
| 1,950 | 1.0000000 | 1.0000000 | 1.0000000 | World Bank Low Income | Ischemic stroke | Deaths | Female | Low physical activity | 1945 to 1954 | 11 |
| 1,955 | 0.9726104 | 0.7961039 | 1.1882507 | World Bank Low Income | Ischemic stroke | Deaths | Female | Low physical activity | 1950 to 1959 | 12 |
| 1,960 | 0.9767288 | 0.7560003 | 1.2619032 | World Bank Low Income | Ischemic stroke | Deaths | Female | Low physical activity | 1955 to 1964 | 13 |
| 1,965 | 1.1621393 | 0.8674675 | 1.5569088 | World Bank Low Income | Ischemic stroke | Deaths | Female | Low physical activity | 1960 to 1969 | 14 |
| 1,970 | 1.3554183 | 0.9606210 | 1.9124698 | World Bank Low Income | Ischemic stroke | Deaths | Female | Low physical activity | 1965 to 1974 | 15 |
| 1,975 | 1.2180029 | 0.7943628 | 1.8675737 | World Bank Low Income | Ischemic stroke | Deaths | Female | Low physical activity | 1970 to 1979 | 16 |
| 1,980 | 1.1124879 | 0.6459255 | 1.9160558 | World Bank Low Income | Ischemic stroke | Deaths | Female | Low physical activity | 1975 to 1984 | 17 |
| 1,985 | 1.0829177 | 0.5337756 | 2.1970104 | World Bank Low Income | Ischemic stroke | Deaths | Female | Low physical activity | 1980 to 1989 | 18 |
| 1,990 | 1.1161304 | 0.3907790 | 3.1878553 | World Bank Low Income | Ischemic stroke | Deaths | Female | Low physical activity | 1985 to 1994 | 19 |
